# KAE ameliorates LPS-mediated acute lung injury by inhibiting PANoptosis through the intracellular DNA-cGAS-STING axis

**DOI:** 10.3389/fphar.2024.1461931

**Published:** 2025-01-07

**Authors:** Yonghu Chen, Xilin Wu, Zhe Jiang, Xuezheng Li

**Affiliations:** College of Pharmacy, Yanbian University Hospital, Yanbian University, Yanji, China

**Keywords:** KAE, cGAS-STING, PANoptosis, acute lung injury, intracellular DNA

## Abstract

**Background:**

Acute lung injury (ALI) is a severe condition characterized by inflammation, tissue damage, and persistent activation of the cyclic GMP-AMP (cGAS)-stimulator of interferon genes (STING) pathway, which exacerbates the production of pro-inflammatory mediators and promotes the progression of ALI. Specific inhibition of this pathway has been shown to alleviate ALI symptoms. Kaempferol-3-*O*-*α*-L-(4″-*E*-p-coumaroyl)-rhamnoside (KAE), an active compound found in the flowers of *Angelica acutiloba* Kitagawa, exhibits anti-inflammatory and antioxidant properties. This study aimed to investigate the molecular mechanisms through which KAE regulates the cGAS-STING pathway in the context of ALI.

**Methods:**

ALI was induced using LPS. Lung damage and anti-inflammatory/antioxidant effects were assessed by H&E staining, lung edema index, and SOD, MDA, and ELISA assays. NO release and mitochondrial membrane potential (MMP) were measured by JC-1 and Griess methods. The impact of KAE on the cGAS-STING pathway and PANoptosis was analyzed using flow cytometry, Western blot, and immunofluorescence.

**Results:**

KAE significantly alleviated lipopolysaccharide-induced pulmonary injury by reducing inflammatory cell infiltration, alleviating pulmonary edema, enhancing antioxidant capacity, and decreasing levels of inflammatory cytokines in mouse lung tissues. In both *in vitro* and *in vivo* analyses, KAE downregulated the expression of key components of the cGAS-STING pathway, including cGAS, STING, p-TBK1, and nuclear factor-κB. KAE also reduced the assembly and activation of the PANoptosome, thereby attenuating apoptosis, necroptosis, and pyroptosis. Additionally, KAE inhibited cGAS activation by restoring the MMP, which reduced the release of cytosolic DNA.

**Conclusion:**

KAE improve ALI by inhibiting the release of cytosolic DNA and suppressing cGAS-STING pathway activation, thereby protecting cells from PANoptosis. Our findings provide valuable insights for the development and application of novel therapeutic strategies for ALI.

## Graphical Abstract

**Figure:**
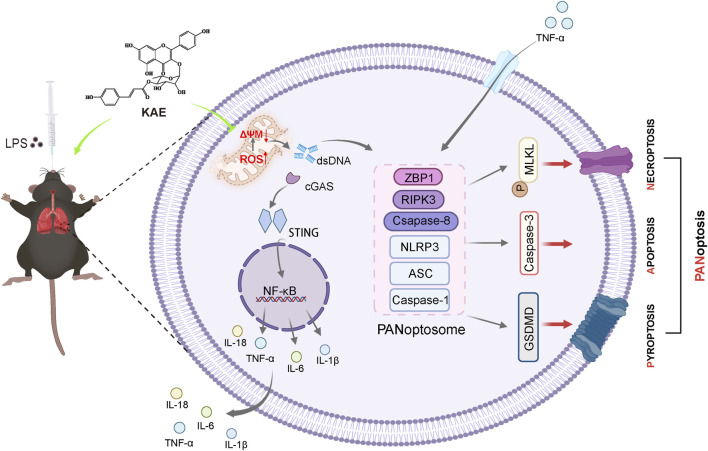
LPS-mediated ALI led to macrophages with elevated ROS and decreased MMP, which increased intracytoplasmic dsDNA content. Additionally, the production of cGAS-activated STING dimers in response to intracytoplasmic dsDNA mediated the release of inflammatory factors (e.g., TNF-α and IL-6). TNF-α further led to the activation of ZBP1, RIPK3, ASC, Caspase-8, and other proteins. ZBP1 senses intracytoplasmic dsDNA, which enhances the formation of the PANoptosome complex, thereby promoting the phosphorylation of MLKL and leading to cell death through the cleavage of Caspase-3 and GSDMD.

## Introduction

Acute lung injury (ALI) is a serious respiratory condition caused by a combination of internal and external factors. It is primarily characterized by uncontrolled oxidative stress, pulmonary edema, and infiltration of inflammatory cells, which can lead to acute respiratory distress syndrome (ARDS) in severe cases (Muhammad et al., 2022; Zhao et al., 2021). While ALI is usually regarded as an early, mild stage of ARDS and features an oxygenation index between 200 and 300 mmHg, the oxygenation index in ARDS is below 200 mmHg. Global epidemiological data show that the incidence of ALI is 10.4%, whereas that of ARDS is higher, with a lethality rate of about 40% in severe cases (Hao Q. et al., 2022; McNicholas et al., 2019). Therefore, reducing the exacerbation of ALI through early treatment may effectively reduce the incidence of ARDS. The development of ALI involves the coordinated actions of multiple cells and factors at different stages. During the inflammatory phase, the release of inflammatory mediators results in the recruitment of neutrophils to damaged lung tissues, where they are actively involved in the clearance of pathogens and dead cells (Cen et al., 2021; Chen et al., 2023b). Subsequently, damaged epithelial cells may undergo epithelial-to-mesenchymal transition, while apoptosis becomes significant in the mid to late stages, contributing to the destruction of the alveolar wall (Xiao K. et al., 2020). Oxidative stress and cellular damage drive persistent inflammation and immune responses (Zhu et al., 2023). This ongoing inflammation exacerbates the lung injury, making the condition more severe. In the final stage, stem cells and fibroblasts are involved in the repair and regeneration of injured lung tissue, creating a complex network of multicellular interactions. The entire process from injury to repair illustrates the intricate nature of ALI. Current treatments for ALI focus on suppressing the inflammatory response and using protective mechanical ventilation. The lack of preventive measures or specific treatments for ALI makes it a critical scientific problem in respiratory care (Cai et al., 2022; Wang J. et al., 2022). Therefore, we should elucidate the pathophysiological mechanisms of ALI and develop effective therapeutic agents.

PANoptosis is a comprehensive mechanism of inflammatory programmed cell death that encompasses apoptosis, pyroptosis, and necroptosis (Chen S. et al., 2023; Place et al., 2022; Wang et al., 2023). The occurrence of PANoptosis involves the phosphorylation of mixed lineage kinase domain-like protein (MLKL) and the activation of Caspase-3 and Gasdermin D (GSDMD) (Lee et al., 2021). PANoptosis is regulated by the upstream receptor Z-DNA binding protein 1 (ZBP1), which serves as the key sensor for the initiation of PANoptosis, triggering the assembly of proteins (e.g., Caspase-1, Caspase-8, ASC) to form the multiprotein PANoptosome complex. This complex induces cells to undergo various forms of cell death (Hao Y. et al., 2022; Karki et al., 2021; Ren et al., 2022). Therefore, dissecting the assembly and activation of the PANoptosome could be invaluable for research into the occurrence of PANoptosis in a variety of disease systems. Studies in this emerging field have shown that PANoptosis activation enhances the immunogenicity of tumor cells and induces the immune system to perform recognition and clearance (Gao et al., 2024), whereas PANoptosis inhibition systematically regulates inflammatory responses, oxidative stress, and tissue damage repair, significantly reducing the pathological symptoms of ALI (Cui et al., 2022; Shi et al., 2024). However, the complex regulatory network of PANoptosis remains unclear, hindering efforts to effectively regulate PANoptosis for the treatment of disease. In-depth study of the specific mechanisms of PANoptosis in ALI could reveal new therapeutic targets and intervention strategies, leading to reduced lung injury and improved patient outcomes.

Activation of intracytoplasmic DNA plays a crucial role in PANoptosis, which is also associated with mitochondria-related intrinsic pathways and cell membrane receptor-mediated extrinsic pathways (Bi et al., 2022; She et al., 2023). The presence of intracytoplasmic DNA serves as a marker for oxidative stress and inflammatory responses within cells, directly affecting mitochondrial function (Xian et al., 2022). Research has shown that reactive oxygen species (ROS) in the cytoplasm can impair mitochondrial function, damaging the mitochondrial membrane and releasing mitochondrial DNA (Wong et al., 2022). Intracytoplasmic DNA can also be released into the cytoplasm in response to external stimuli or endogenous factors, such as viral infection, oxidative stress, or DNA damage. These DNA fragments act as endogenous damage-associated molecular patterns (DAMPs), inducing the activation of immune cells and triggering inflammatory responses (De Gaetano et al., 2021; Miller et al., 2021). Additionally, intracytoplasmic DNA activates cellular DNA-sensing mechanisms, such as the cyclic GMP-AMP synthase (cGAS)- stimulator of interferon genes (STING) pathway, which further triggers apoptosis. In this process, intracytoplasmic DNA is recognized by cGAS, activating STING and leading to the production of interferon and other inflammatory mediators (Hou et al., 2023). These mediators promote cell death through various pathways, including downstream inflammatory signaling, which exacerbates mitochondrial damage and intracellular death pathways. Studies have demonstrated that STING pathways activated by airway silica or smoke exposure lead to cell death, self-double-stranded DNA (dsDNA) release, and ALI (Benmerzoug et al., 2018; Nascimento et al., 2019). The STING activator dimeric amidobenzimidazole (diABZI) has been found to induce Caspase-3 activation and GSDMD protein cleavage, as well as MLKL phosphorylation in lung tissues, promoting apoptosis, pyroptosis, and necroptosis. Furthermore, the upregulation of ZBP1 and increased protein expression of Caspase-8, ASC, and receptor-interacting protein kinase 3 (RIPK3) in lung tissues facilitate the assembly of the PANoptosome, inducing PANoptosis in ALI (Messaoud-Nacer et al., 2022). Interestingly, ursodeoxycholic acid has been shown to reduce PANoptosis in ALI through the cGAS-STING pathway, significantly alleviating symptoms of ALI (He et al., 2023). Therefore, further investigation into the role and mechanisms of the intracytoplasmic DNA-cGAS-STING axis in ALI will enhance our understanding of PANoptosis in ALI and provide a theoretical basis for the development of new therapeutic strategies.

**FIGURE 1:**
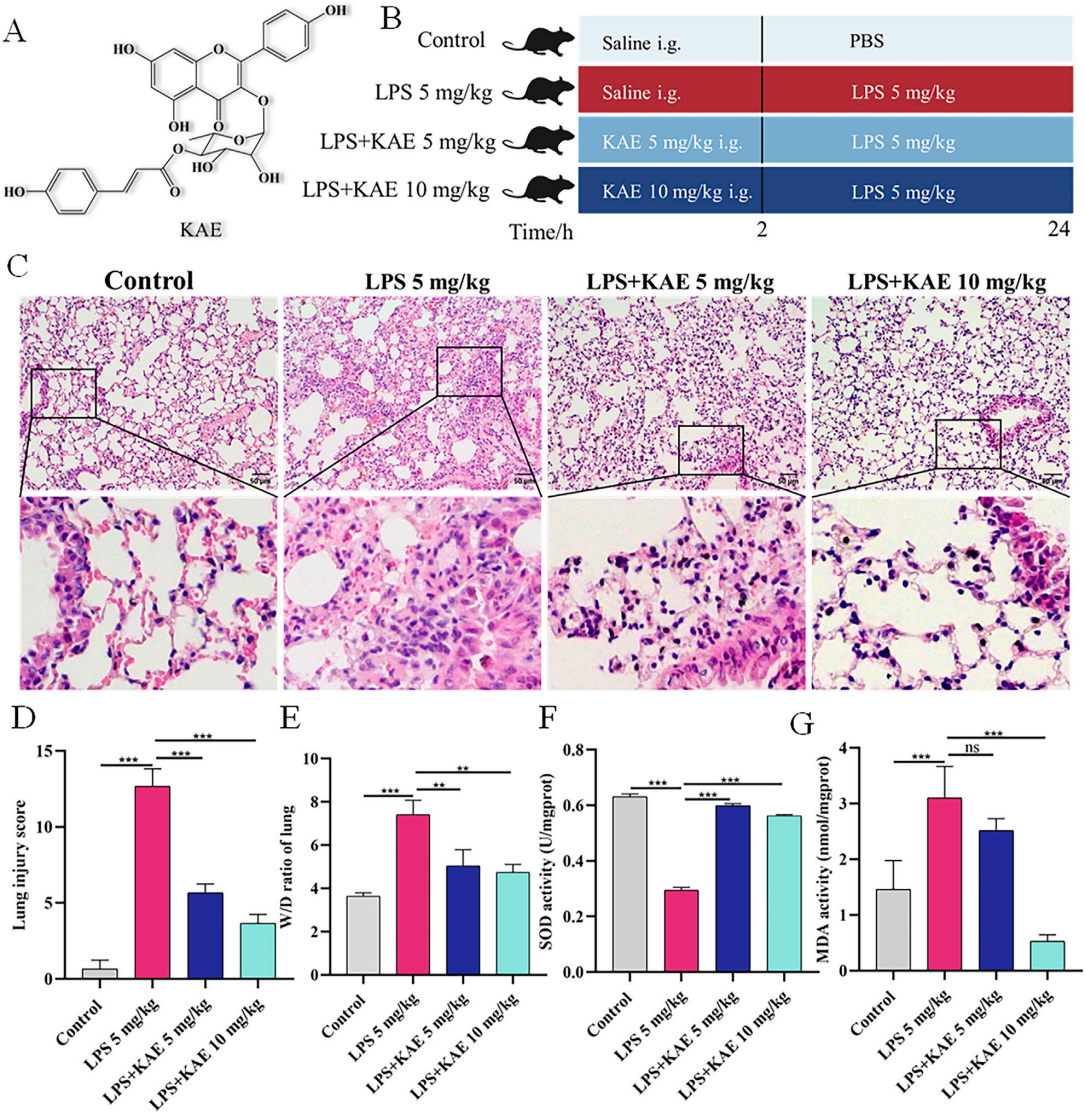
KAE protects against LPS-mediated ALI and increases lung tissue antioxidant levels. **(A)** Structural formula of KAE. **(B)** Timeline of KAE treatment and ALI induction in mice. **(C, D)** H&E staining **(C)** and tissue damage scores **(D)** of lung tissues (means ± SEM; n = 8). **(E)** Lung tissue pulmonary oedema index (means ± SEM; n = 8). **(F, G)** Lung tissue SOD and MDA activity levels (means ± SEM; n = 5). **P < 0.01, ***P < 0.001, calculated by one-way ANOVA followed by Tukey’s test.

Kaempferol-3-*O*-*α*-L-(4″-*E*-p-coumaroyl)-rhamnoside (KAE; Figure 1A) extracted from *Angelica acutiloba* Kitagawa flowers has a wide range of pharmaceutical uses (Chen et al., 2023c; Yang et al., 2019). Previous studies found that KAE could ameliorate nicotinic nitrosyl derivative-induced injury and reduce the occurrence of pyroptosis in MLE-12 epithelial cells, while also treating lipopolysaccharide (LPS)-induced lung injury and reducing inflammation in mouse lungs; however, the specific regulatory mechanism is unclear. Therefore, the aims of this study were to investigate the therapeutic mechanism of KAE in ALI, provide new insights for its treatment, and establish a basis for the rational clinical application of *A. acutiloba*.

## Materials and methods

### Cell culture and source of KAE

DMEM with 10% fetal bovine serum and 1% streptomycin and penicillin (Gibco, United States) was used to culture RAW264.7 cells (Kunming Cell Bank, China). Following stimulation with 1 μg/mL LPS (Sigma-Aldrich, United States) and treatment with different concentrations of KAE, cells were cultured for 24 h at 37°C in 5% CO_2_ in an incubator (Thermo, United States) and collected. KAE was isolated from the flowers of *A. acutiloba* and identified following the methods described by Chen et al. (2023c).

### Experimental animals

A total of 32 male C57BL/6 mice (specific pathogen-free grade; body weight, 20–22 g; age, 6–8 weeks) were purchased from and housed in the Yanbian University Animal Center at 24°C, with free diet and water intake for 1 week after acclimatization. All animal experiments were approved by the Animal Ethics Committee of Yanbian University Hospital (2024200).

### Grouping and drug administration

After 1 week of acclimatization, the mice were randomly divided into four treatment groups: Control, model (LPS 5 mg/kg), LPS + KAE 5 mg/kg, and LPS + KAE 10 mg/kg. Following anesthetization, the ALI model was established by intratracheal drip of LPS (100 μg per mouse) or saline (Control group) for 24 h. Two hours before LPS induction, the mice were administered the appropriate concentration of KAE (dissolved in PBS) or an equal volume of PBS (Control group) by gavage; 24 h later, bronchoalveolar lavage fluid (BALF) and lung tissues were collected by lavage using pre-cooled PBS.

### Dihydroethidium (DHE) staining and MMP detection

1.8 × 10^5^ cells/mL per well in 6-well plates were spread, and after 24 h of drug action, each well was washed once using PBS, 1 mL of serum-free medium and 1 mL of JC-1 working solution were added to each well and mixed respectively (Beyotime, China), and placed in the incubator to incubate for 20 min, and the washing solution was washed twice, and the images were acquired using a fluorescence microscope (Echo Laboratories, United States) to capture images.

After the cells were spread and administered, the supernatant was aspirated and discarded, and washed once using PBS, 2 mL of serum-free medium containing 10 μM of DHE (APExBIO, United States) was added to each well, respectively, and the cells were returned to the incubator for 30 min, washed twice with PBS, and then incubated for 10 min with DAPI protected from light to acquire images.

### Hematoxylin and eosin (H&E) staining

Lung tissue specimens for pathological examination were fixed in 4% paraformaldehyde. After sequential dehydration with graded ethanol, the tissues were embedded in paraffin using an embedding machine (Thermo, United States). The blocks were cut into 5-μm sections, baked at 60°C for 6 h, deparaffinized with xylene, washed with graded ethanol, and rinsed with PBS. The sections were stained with H&E (APExBIO, United States) in strict accordance with the manufacturer’s instructions, sealed with neutral resin (Solarbio, China), and observed under a light microscope (Olympus Corporation, Japan). Evaluator-blinded scoring was used to assess alveolar septal thickening, inflammation, hemorrhage, and edema, using the following scale: none (score = 0), mild (score = 1), moderate (score = 2), severe (score = 3), and very severe (score = 4). The lung injury score was the sum of the scores for each category (He et al., 2022).

### Detection of lung oedema and oxidative stress indicators

Mouse lung specimens were collected and weighed as wet weight immediately after removal, and then weighed again as dry weight after drying in a 75°C drying oven for 48 h. Mouse lung tissue oedema was assessed by measuring the wet-to-dry weight (W/D) ratio. Another appropriate amount of lung tissue was taken and fully ground and then quantified by the bicinchoninic acid assay (BCA) method (Solarbio, China), and the levels of malondialdehyde (MDA) and superoxide dismutase (SOD) (NanJing JianCheng Bioengineering Institute, China) in the lung tissues were measured according to the manufacturer’s instructions, which were used to assess the level of oxidative stress in the lung tissues.

### Cell viability and nitric oxide (NO) experiments

Except for the Control group, LPS was given to construct an *in vitro* RAW264.7 injury model and treated with different concentrations of KAE or dexamethasone 1 μM (DEX) (MedChemExpress, United States). After 24 h of action, 10 μL of cell counting kit-8 (CCK8) (APExBIO, United States) was added to each well or the supernatant NO (APExBIO, United States) was detected using the Griess Reagent method, with optical density readouts obtained at 450 nm or 540 nm via a microplate reader.

### Immunofluorescence experiments

Paraffin containing lung tissue was cut into 4 μm sections (Leica Biosystems, Germany) and subjected to a dewaxing and hydration process, microwave heating to repair antigen and remove non-specific binding. Or cell crawling sheets were fixed with 4% paraformaldehyde (Beyotime, China), closed with 5% BSA for 1 h at room temperature (Sigma-Aldrich, United States), and titrated with diluted GSDMD (Cell Signaling Technology, United States), STING, RIPK3, Caspase-3, Caspase-8, dsDNA (Proteintech, China) overnight at 4°C, incubation with secondary antibody (coupled fluorescent FITC, CY3) (APExBIO, United States) for 1 h. DAPI was re-stained for 10 min, and anti-fluorescence quenching solution was added dropwise, and images were acquired.

### ELISA for the detection of inflammatory factors

Lung tissues were lavaged using 3 mL of PBS, and approximately 2.4 mL of BALF was recovered and supernatants were collected. IL-6, IL-1β, TNF-α and IL-18 levels were detected according to the manufacturer’s (Elabscience, China) instructions.

### Cell death was detected by flow cytometry

The drug-treated cells were collected by centrifugation and resuspended with PBS, the cells were washed by centrifugation again, 500 μL of buffer was added to resuspend the cells, and then Annexin V-FITC 5 μL (MEC COMPANY LTD., Japan) was added, and incubated for 20 min away from light, 2 μL of PI dye was added to mix well, and the changes in cell death were analysed using flow cytometry (Becton, Dickinson and Company, United States) to analyse changes in cell death.

### Western blot

Mouse lung tissue was taken on ice for grinding or cells were collected on ice and then centrifuged and quantified by BCA method. Polyacrylamide gel was electrophoresed and transferred to ntrocellulose membrane (NC) membrane. Closure was performed using 5% skim milk powder or 5% BSA. Dropwise addition of primary antibody overnight at 4°C, these include GSDMD (39754S), p-TBK1 (5,483, Cell Signaling Technology), p- MLKL (ab196436, Abcam), cGAS (29958-1-AP), ZBP1 (13285- 1-AP), NF-κB (10745-1-AP), NLRP3 (19771-1-AP), MLKL (66675-1-Ig), Caspase-1 (22915-1-AP), Caspase-3 (19677-1-AP), Caspase-8 (66093-1-Ig), GAPDH (10494-1-AP), ASC (10500-1-AP), STING (19851-1-AP), β-Actin (20536-1-AP) from Proteintech. After washing, they were immersed in secondary antibody working solution (1:20,000, LI-COR Biosciences). Processing was visualised using a dual-colour infrared laser imaging system (LI-COR Odyssey Clx, United States) and analysed using ImageJ 8.0.

### Statistical analysis

All data in this study means ± SEM and were analysed and plotted using GraphPad Prism 7.0, and differences between groups were analysed using one-way anova.

## Results

### KAE improves LPS-mediated ALI symptoms

To investigate the effect of KAE pre-treatment on the symptoms of ALI, we used tracheal drip LPS (5 mg/kg) to establish a mouse ALI model (Figure 1B). Mouse lung tissues collected at 24 h post induction were observed by histopathology (Figure 1C). In the Control group, the lung tissue structure was clear, the alveolar walls were slim, the alveoli were intact and homogeneous, and there were no hemorrhagic spots or obvious inflammatory cell infiltration. By contrast, the LPS 5 mg/kg group showed alveolar walls significantly thickened by inflammatory cell infiltration, indicating successful ALI modeling. Compared with the LPS 5 mg/kg group, the two groups pre-treated with KAE exhibited reduced lung tissue damage. Specifically, both doses of KAE significantly reduced the lung tissue injury score and the pulmonary edema index (Figures 1D, E), indicating that KAE had a therapeutic effect of improving the symptoms of ALI in mice.

Oxidative stress-induced lung tissue injury is an important form of ALI, and studies have shown that oxidative stress imbalance is one of the pathogenic mechanisms of ALI (Lin et al., 2021). Therefore, we measured lung activity of SOD and oxidative end-product MDA in the four groups (Figures 1F, G). The LPS 5 mg/kg group showed significantly lower SOD activity and significantly higher MDA activity compared with the Control group. KAE intervention significantly reversed these changes in SOD and MDA activity in lung tissues, suggesting that KAE may increase systemic antioxidant levels to reduce oxidative stress in ALI lung tissues.

### KAE inhibits cGAS-STING pathway activation to attenuate the inflammatory response in ALI

**FIGURE 2:**
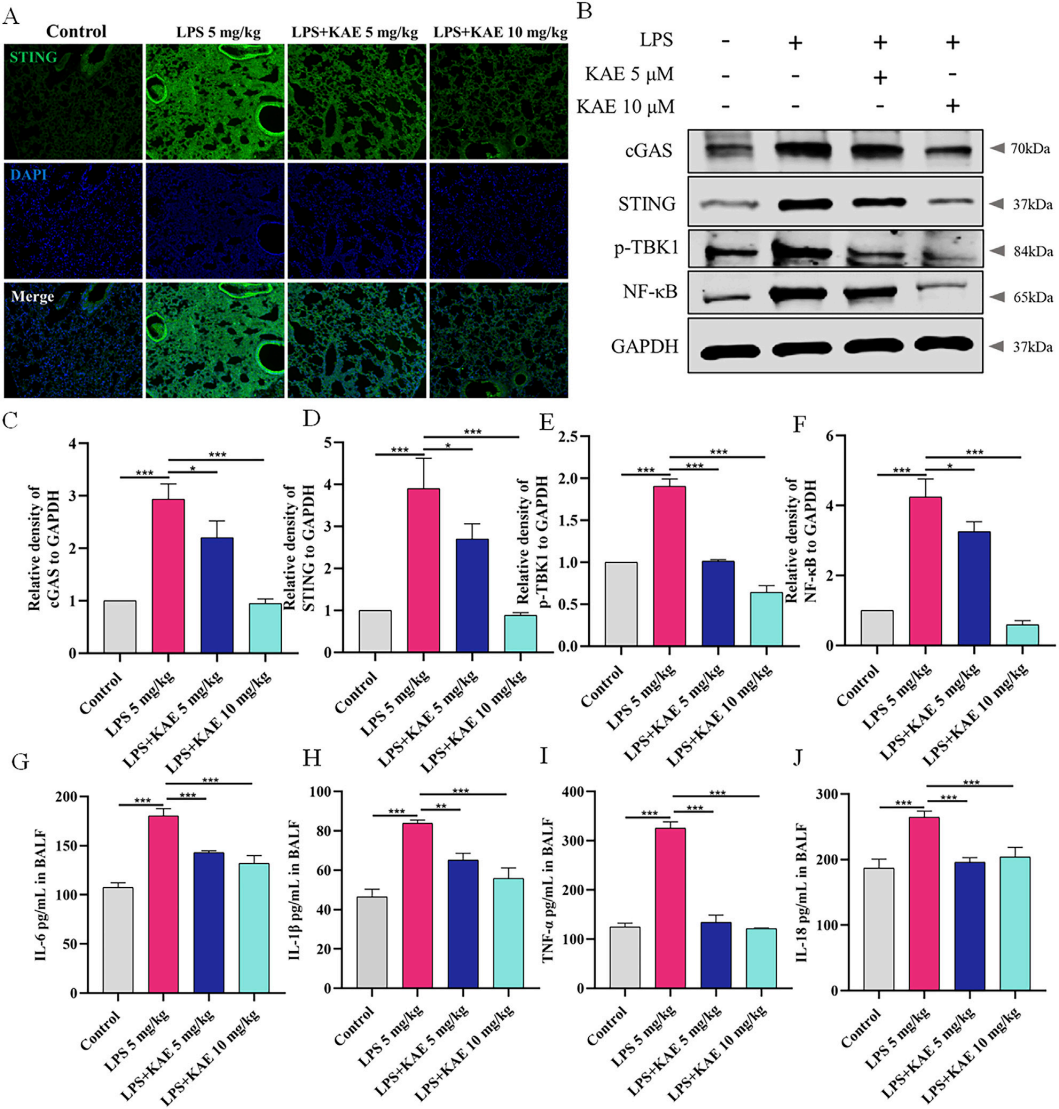
KAE inhibits cGAS-STING pathway activation in ALI to attenuate the inflammatory response. **(A)** STING immunofluorescence in lung tissues. **(B–F)** Western blot **(B)** and quantification **(C–F)** of the indicated cGAS-STING pathway proteins in lung tissues (means ± SEM, n = 3). **(G–J)** ELISAs of the indicated inflammatory cytokines in BALF (means ± SEM, n = 5). *P < 0.05, **P < 0.01, ***P < 0.001, calculated by one-way ANOVA followed by Tukey’s test.

As a natural immune signaling axis, cGAS-STING responds to intracellular DNA by triggering inflammatory responses (Chung et al., 2019; Kwon and Bakhoum, 2020). To elucidate the therapeutic mechanism of KAE in ALI, we examined the expression of STING in lung tissues (Figure 2A) using immunofluorescence staining. The Control group showed a very low level of STING expression, whereas the LPS 5 mg/kg group showed high expression in the lung tissues. Pre-treatment with either dose of KAE effectively attenuated the fluorescence intensity of STING in lung tissues. Next, we evaluated the lung expression of other proteins in the STING pathway (Figures 2B–F). Western blot experiments showed low expression of cGAS, STING, phosphorylated TANK-binding kinase 1 (p-TBK1), and nuclear factor-κB (NF-κB) in the normal lung tissues of the Control group. Although this pathway was significantly activated by LPS-induced injury, the KAE intervention significantly inhibited the expression and activation of cGAS, STING, p-TBK1, and NF-κB. To further evaluate the inflammation in lung tissues, we used ELISAs to examine the levels of IL-1β, IL-6, TNF-α, and IL-18 in BALF (Figures 2G–J). This revealed that all four cytokines were significantly elevated in injured lung tissues, but were reduced by pre-treatment with KAE, which lowered the inflammatory response to achieve an ameliorating effect on ALI.

### KAE inhibits PANoptosis to reduce cell death in ALI

**FIGURE 3:**
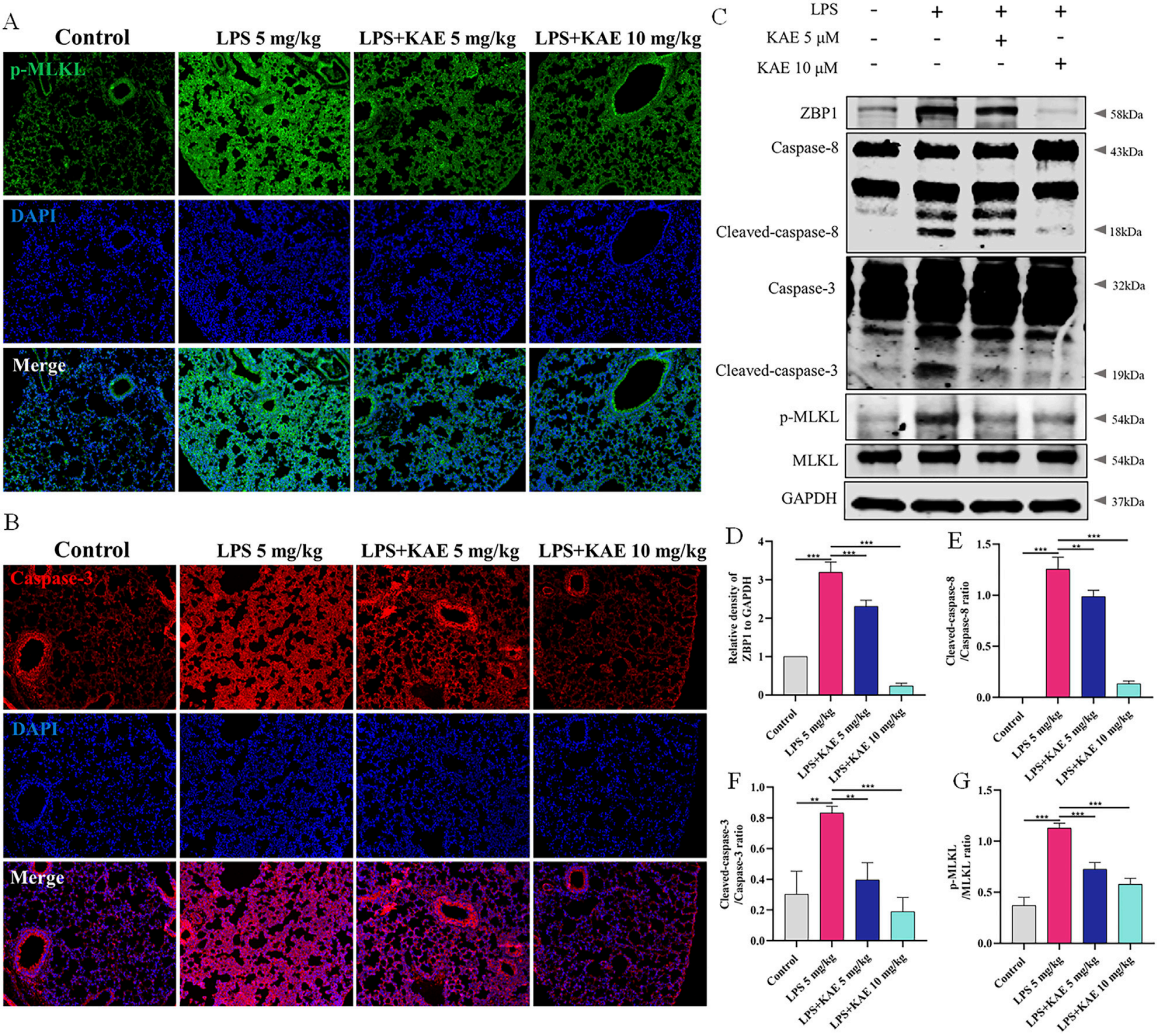
KAE reduces the occurrence of necroptosis and apoptosis in ALI. **(A, B)** Immunofluorescence staining of p-MLKL and Caspase-3 in lung tissues. **(C–G)** Western blot **(C)** and quantification of proteins related to necroptosis and apoptosis in lung tissues (means ± SEM; n = 3). **P < 0.01, ***P < 0.001, calculated by one-way ANOVA followed by Tukey’s test.

Studies have shown that STING can mediate apoptogenesis (Li et al., 2019; Victorelli et al., 2023), and therefore we further evaluated the pattern of cell death in ALI model mice by examining the expression of necroptosis- and apoptosis-related proteins in lung tissues. Western blot showed that, in the LPS 5 mg/kg group, the DNA receptor ZBP1 was activated, Caspase-8 and Caspase-3 were cleaved and activated, and MLKL phosphorylation was increased. Treatment with KAE significantly reduced the expression and reversed the activation of these LPS-induced proteins (Figures 3C–G). Using immunofluorescence, we demonstrated that KAE reversed the LPS-induced enhancement of the fluorescence intensities of the necroptosis signature protein p-MLKL and the apoptosis marker Caspase-3 (Figures 3A, B). These results suggested that both necroptosis and apoptosis are involved in the ALI process, which may be triggered by ZBP1 in response to intracytoplasmic DNA, with KAE reducing the occurrence of this death to protect against lung tissue damage.

**FIGURE 4:**
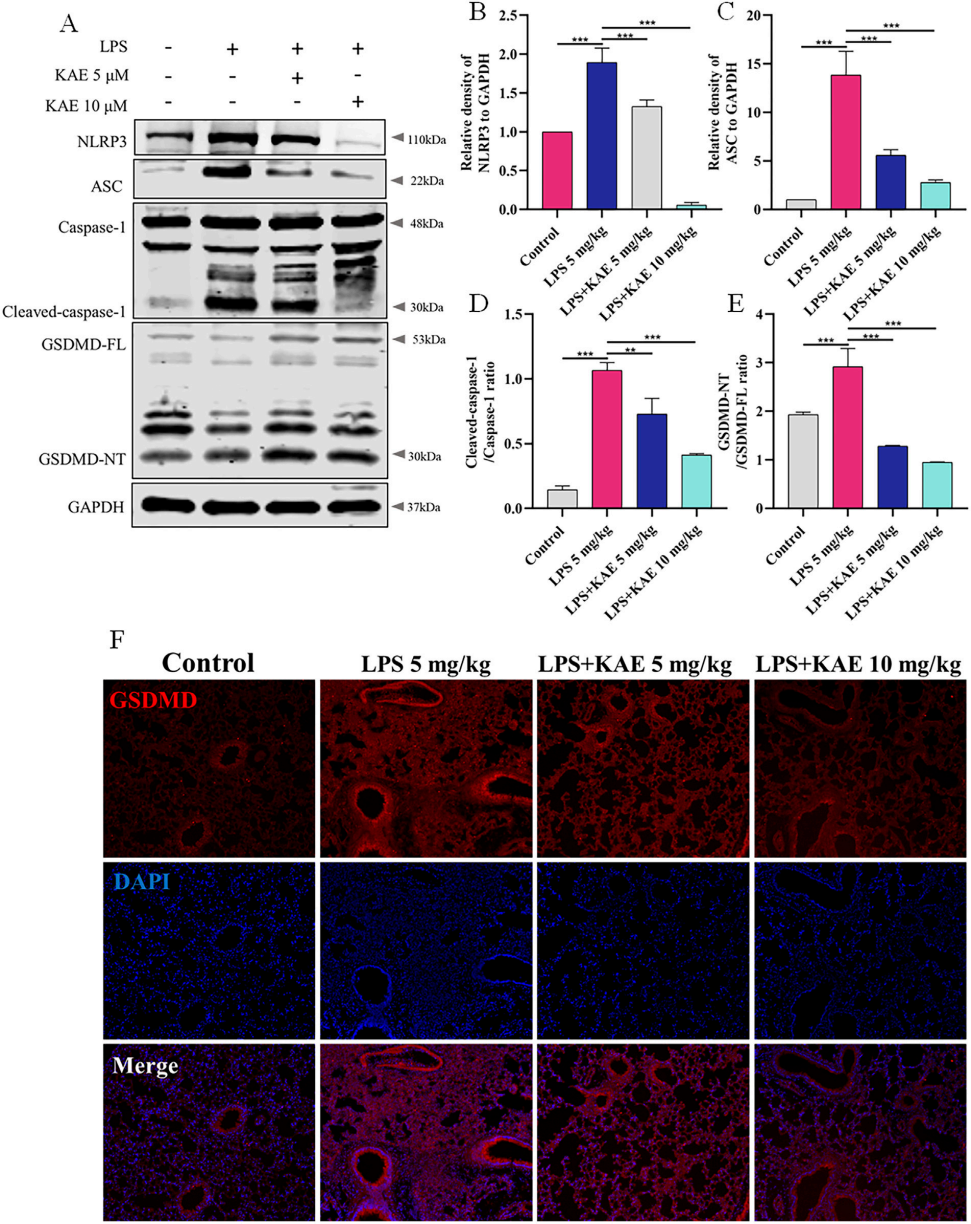
KAE inhibits pyroptosis in ALI. **(A–E)** Western blot **(A)** and quantitative analysis **(B–E)** of NLRP3 signaling pathway and pyroptosis-related proteins in lung tissues (means ± SEM; n = 3). **(F)** Immunofluorescence of GSDMD expression in lung tissue. **P < 0.01, ***P < 0.001, calculated by one-way ANOVA followed by Tukey’s test.

The inflammatory response is an important cause of tissue damage. Nucleotide-binding domain-like receptor protein 3 (NLRP3), as a hub of the inflammatory response, mediates NLRP3 inflammasome activation in response to external stimuli, leading to the transfer of GSDMD to the cell membrane and triggering pyroptosis (Wang et al., 2024; Xiao J. et al., 2020; Zhang et al., 2021). Therefore, we further examined the occurrence of pyroptosis in lung tissues using Western blot (Figures 4A–E). Intervention with KAE not only reduced the protein expression levels of NLRP3, ASC, and cleaved-Caspase-1, it also decreased NLRP3 inflammasome activation and GSDMD cleavage, as confirmed by immunofluorescence experiments (Figure 4F). These findings are consistent with previous reports of ALI symptom attenuation mediated through inhibition of NLRP3 inflammasome activation (Zeng et al., 2023), which may account for the therapeutic effect of KAE in ALI.

### KAE-mediated inhibition of the cGAS-STING pathway *in vitro* reduces cell death

**FIGURE 5:**
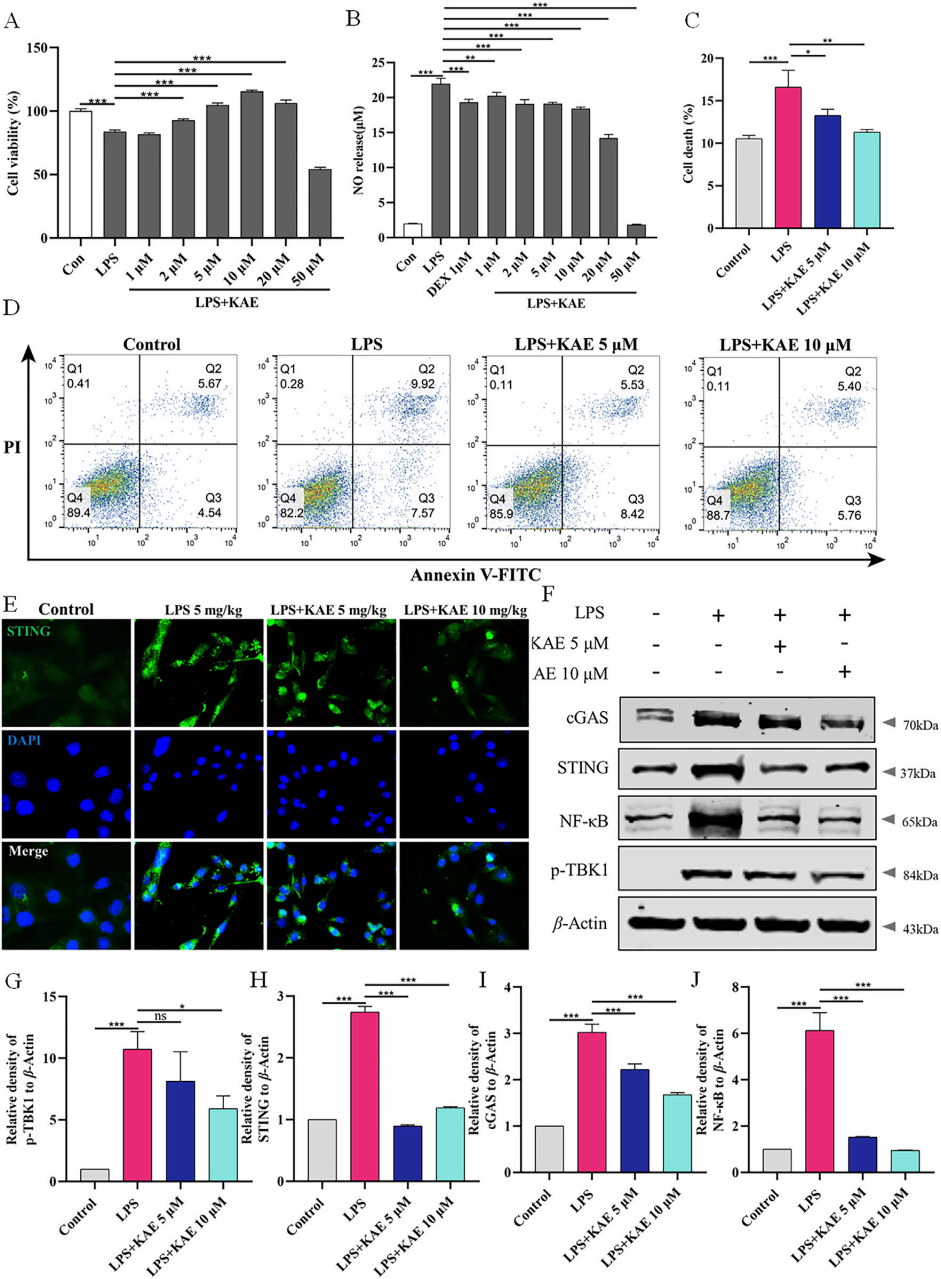
KAE reduces LPS-mediated cell death *in vitro*. **(A, B)** Effects of KAE on viability and NO release of RAW264.7 cells. **(C, D)** Flow cytometry detection **(C)** and quantitative analysis **(D)** of cell death. **(E–J)** Immunofluorescence **(E)**, Western blot **(F)** and quantification of the expression of cGAS-STING pathway proteins in RAW264.7 cells. Graphical data are presented as means ± SEM; n = 3. *P < 0.05, **P < 0.01, ***P < 0.001, calculated by one-way ANOVA followed by Tukey’s test.

Next, we investigated the detailed mechanism by which KAE inhibits the cGAS-STING pathway and PANoptosis to ameliorate ALI. LPS (1 μg/mL) was used to induce RAW264.7 cells to establish an *in vitro* cell injury model. Assays of RAW264.7 cell viability and NO release showed that KAE at 2 μM, 5 μM, and 10 μM significantly reversed LPS-induced cell injury and reduced the release of NO compared with DEX at 1 μM, suggesting that KAE reduces the inflammatory response induced by LPS (Figures 5A, B). To simulate the dose and treatment effects of KAE administered *in vivo*, 5 μM and 10 μM KAE were used for subsequent *in vitro* experiments.

The effect of KAE on cell death *in vitro* was evaluated using flow cytometry detection of living and dead cells. LPS-induced injury to RAW264.7 cells resulted in a significant increase in dead cells that was significantly reversed by KAE intervention at Figures 5C, D. Additionally, Western blot and immunofluorescence assessments of cGAS-STING pathway proteins showed that *in vitro* treatment with KAE significantly reduced the expression of cGAS and STING, which in turn inhibited the phosphorylation of the downstream protein, TBK1, and blocked inflammation (Figures 5E–J). Taken together, these findings demonstrated that KAE can protect against LPS-induced cell injury and reduce the occurrence of cell death *in vitro*.

### KAE inhibits PANoptosome assembly *in vitro*

**FIGURE 6:**
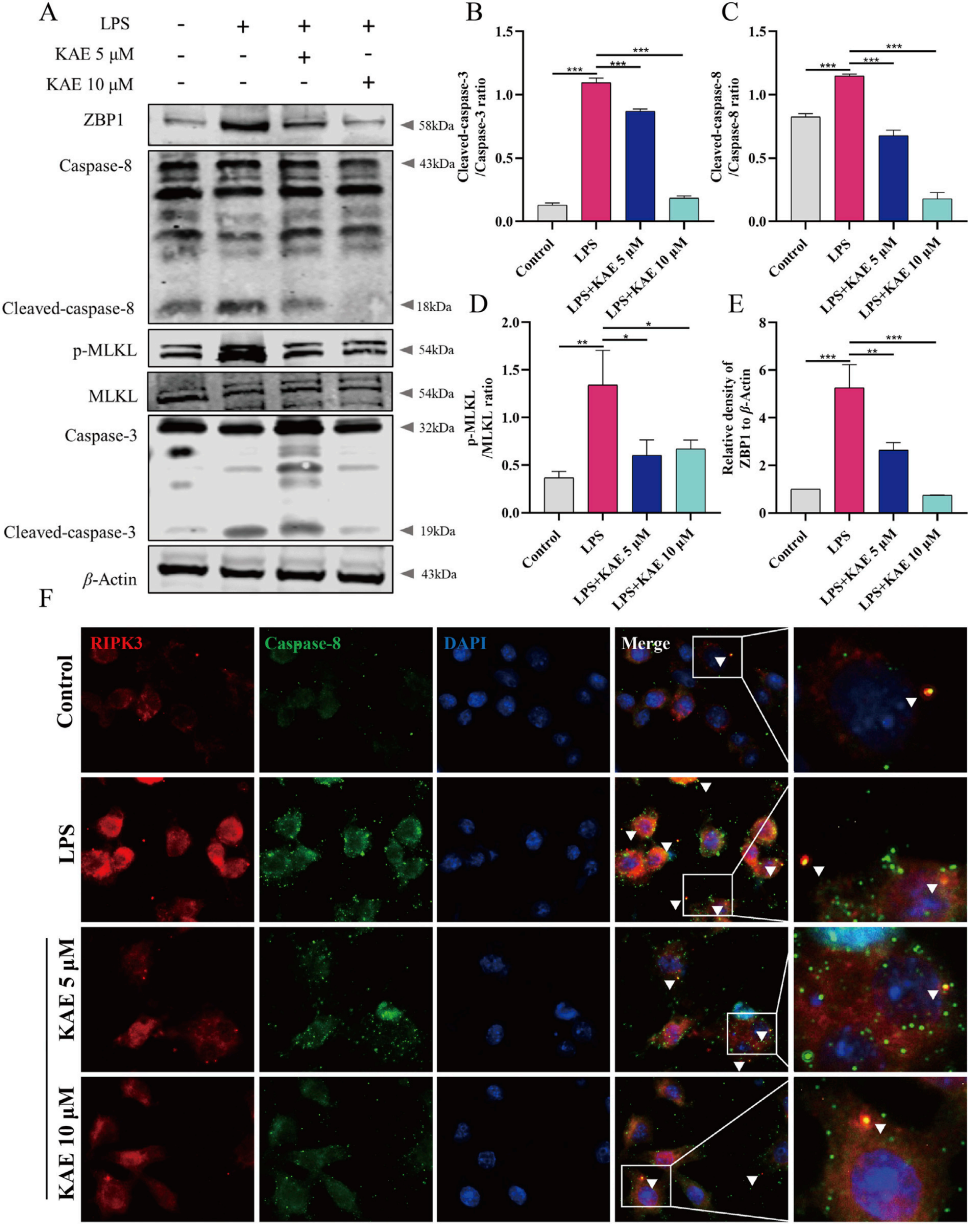
Inhibition of apoptosis and necroptosis by KAE leads to reduced binding between RIPK3 and Caspase-8. **(A–E)** Western blot **(A)** and quantification **(B–E)** of cell death-associated proteins in cells. **(F)** Immunofluorescence detection of RIPK3 and Caspase-8 in cells. Graphical data presented as means ± SEM; n = 3, *P < 0.05, **P < 0.01, ***P < 0.001, calculated by one-way ANOVA followed by Tukey’s test.

ZBP1, as an upstream activator of PANoptosis, promotes the assembly of RIPK3, Caspase-8, ASC, Caspase-1, and other proteins to form the PANoptosome, which induces cells to undergo pyroptosis, necroptosis, apoptosis, and other modes of cell death. Therefore, we assessed the effect of KAE on PANoptosome assembly in RAW264.7 cells *in vitro*. Western blot detection of apoptosis- and necroptosis-related proteins revealed significant upregulation of ZBP1 protein, cleavage and activation of Caspase-3 and Caspase-8, and significant elevation of the phosphorylation level of necroptosis protein MLKL under LPS-mediated cellular injury. KAE treatment significantly reversed these changes in protein expression (Figures 6A–E). Furthermore, immunofluorescence showed a significant reduction in RIPK3 binding to Caspase-8 protein in cells under KAE treatment (Figure 6F).

**FIGURE 7:**
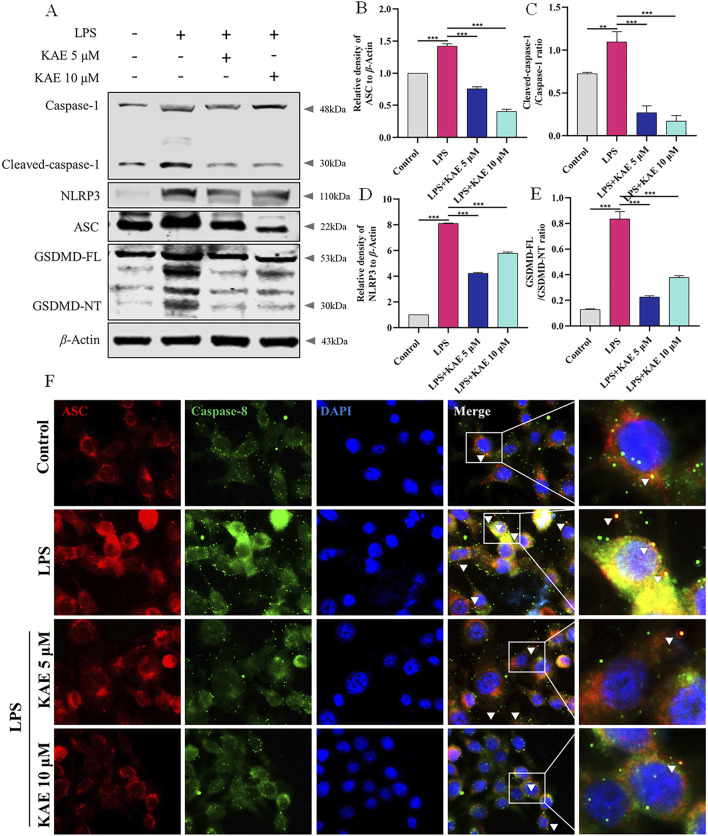
KAE inhibits LPS-mediated pyroptosis and reduces PANoptosome activation and assembly. **(A–E)** Western blot **(A)** and quantification **(B–E)** of pyroptosis-related proteins. **(F)** Immunofluorescence staining of ASC and Caspase-8 expression in cells. Graphical data presented as means ± SEM; n = 3. **P < 0.01, ***P < 0.001, calculated by one-way ANOVA followed by Tukey’s test.

NLRP3 both mediates and participates in the inflammatory response, along with activating the assembly of the PANoptosome (Yan et al., 2023). Therefore, we used Western blot to examine NLRP3-related proteins in RAW264.7 cells. LPS-injured cells exhibited activation of NLRP3, increased expression of ASC and cleavage-activated Caspase-1, and activation of the pyroptosis protein GSDMD, which is involved in the injury process. KAE intervention inhibited the NLRP3 signaling pathway while decreasing the expression of GSDMD (Figures 7A–E). To further observe the effect of KAE on PANoptosome binding, we labeled ASC and Caspase-8, which revealed that the high expression levels of ASC and Caspase-8 in LPS-treated cells was accompanied by a significant increase in their binding. This binding was reduced by intervention with KAE (Figure 7F). Combined with the previous results, we concluded that KAE significantly reduced binding between RIPK3 and Caspase-8. These findings indicated that the activation and assembly of the PANoptosome are key steps in the initiation of multiple forms of programmed cell death, and that the inhibition of cell death mediated by KAE *in vitro* is associated with inhibition of PANoptosome activation and assembly.

### KAE restores MMP to reduce intracytoplasmic DNA release

**FIGURE 8:**
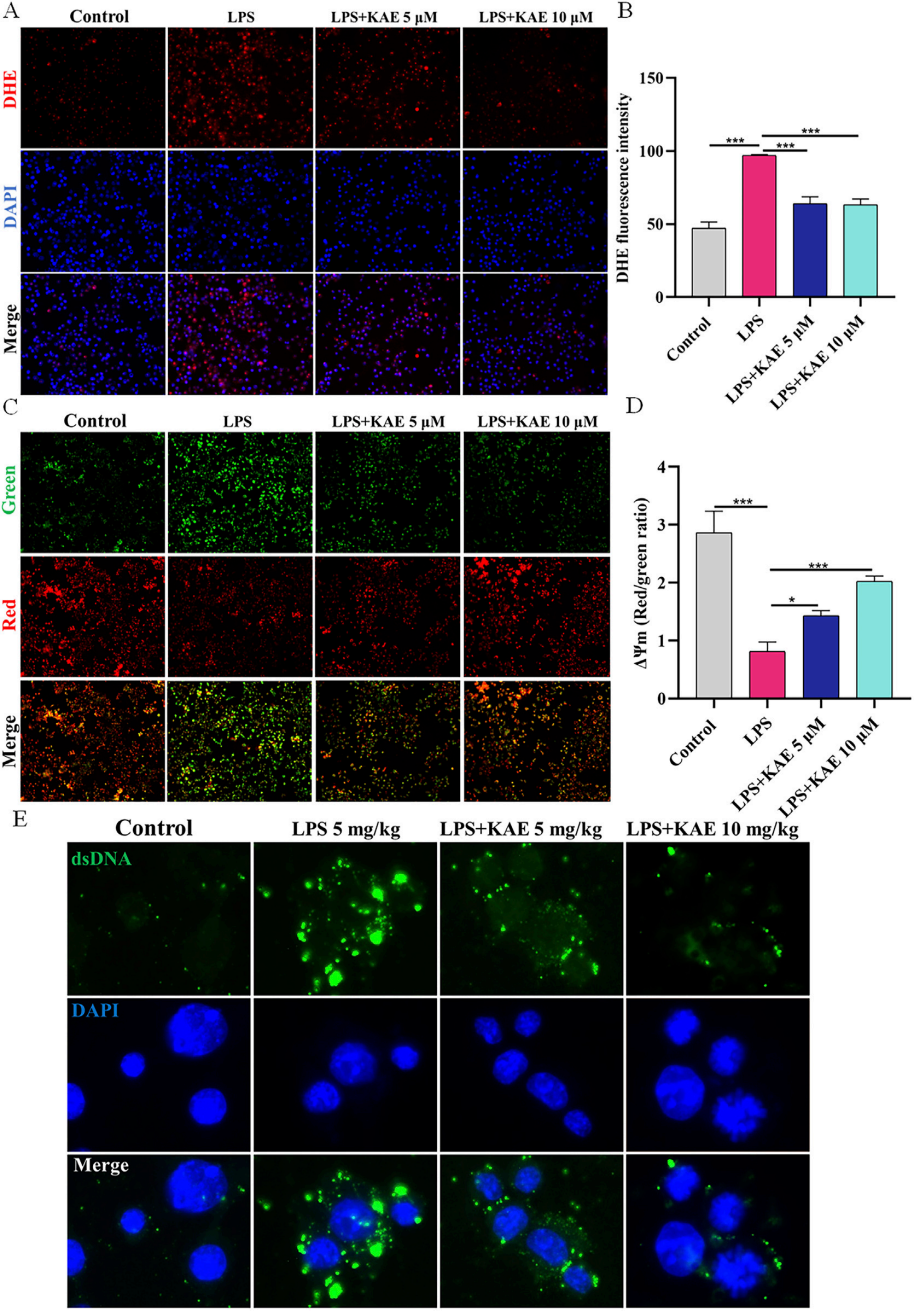
KAE restores MMP to reduce the release of intracytoplasmic DNA *in vitro*. **(A, B)** DHE assay of ROS content in RAW264.7 cells. **(C, D)** JC-1 assay of MMP in RAW264.7 cells. **(E)** Immunofluorescence detection of dsDNA in the cytoplasm of RAW264.7 cells. DAPI was used to stain DNA. Graphical data presented as means ± SEM; n = 3. *P < 0.05, ***P < 0.001, calculated by one-way ANOVA followed by Tukey’s test.

Having demonstrated that LPS-induced ALI *in vivo* and cellular injury *in vitro* could activate the intracytoplasmic DNA receptors cGAS and ZBP1, triggering a series of responses to lung inflammation that further enhanced the assembly of the lung PANoptosome, it became evident that inhibition of cytoplasmic DNA production suppresses the activation of cGAS-STING and PANoptosis. Therefore, we hypothesized that the effects of KAE on these events is exerted through a reduction in intracytoplasmic DNA production. We assessed the ROS content and MMP of RAW264.7 cells using DHE and JC-1 assays, respectively. Compared with untreated control cells, LPS-treated cells exhibited decreased the MMP (Figures 8A, B) and increased intracytoplasmic ROS content (Figures 8C, D). Treatment with KAE increased the MMP while decreasing the content of intracytoplasmic ROS, suggesting that KAE reduces mitochondrial damage and thus mitochondrial DNA leakage, possibly by restoring the MMP. Next, we used immunofluorescence to detect the dsDNA in cells. This revealed that, while LPS stimulation significantly increased the amount of cytoplasmic dsDNA compared with that in untreated cells, intervention with KAE significantly reduced it (Figure 8E). These findings indicated that KAE may reduce lung tissue damage by protecting against mitochondrial damage and reducing intracytoplasmic dsDNA release, which in turn inhibits the activation of DNA receptors ZBP1 and cGAS.

## Discussion

The DNA sensor cGAS recognizes and binds to DNA that leaks into the cytoplasm in response to cellular damage (Peng et al., 2024; Zhang et al., 2022), activating STING dimers, which promote the expression of type I interferons and NF-κB (Hong et al., 2022; Liang et al., 2022). Furthermore, studies have shown that prolonged activation of the cGAS-STING pathway impairs the self-repair ability of the lungs, leading to persistent inflammation and tissue damage (Wang N. N. et al., 2022; Wei et al., 2024). This pathway has emerged as a novel target for the treatment of ALI. Specific inhibition of the activation of this pathway may help to control the inflammatory process, thereby ameliorating ALI. Therefore, we established a mouse model of LPS-induced ALI to evaluate the ameliorative effect of KAE on lung tissues with ALI, which revealed that KAE reduced inflammatory factors in BALF *ex vivo*. We also evaluated the activation of the cGAS-STING pathway in mouse lung tissues and RAW264.7 cells. As expected, KAE effectively inhibited the expression of STING, reducing the expression of p-TBK1 and NF-κB in the organism, and blocking the inflammatory injury to lung tissues. These findings suggested that KAE controls the occurrence of lung inflammation by regulating cGAS-STING activity, thus reducing lung injury.

Sustained activation of the cGAS-STING signaling pathway has been shown to lead to a convergent form of programmed cell death known as PANoptosis (Messaoud-Nacer et al., 2022), which exacerbates lung tissue damage and dysfunction. In ALI, PANoptosis results in extensive death of alveolar epithelial and endothelial cells, disrupting the lung barrier and increasing pulmonary edema and inflammatory infiltrates, which worsen dyspnea and impair oxygenation (D'Alessandro-Gabazza et al., 2022). To investigate this further, we performed a targeted examination of apoptosis, necroptosis, and pyroptosis-related proteins in lung tissues. Our study found that KAE significantly reduced the cleavage of caspase family proteins, phosphorylation of MLKL, and activation of GSDMD, indicating that KAE inhibits PANoptosis in ALI. To further elucidate the therapeutic mechanism, we examined its effect on LPS-induced damage to RAW264.7 macrophages and discovered that KAE prevented macrophage death. The multiprotein PANoptosome complex plays a central role in PANoptosis, with core components that include ASC, Caspase-1, Caspase-8, and RIPK3 (Huang et al., 2022; Sundaram et al., 2023). This complex facilitates cell death by activating key proteins, such as MLKL, Caspase-3, and GSDMD (Pandeya and Kanneganti, 2024). Activation of the PANoptosome leads to the formation of cellular membrane pores and the release of inflammatory factors, triggering a robust inflammatory response (Lu et al., 2021). In inflammatory diseases such as ALI, PANoptosome activation is closely linked to tissue damage and disease progression (Guo et al., 2023; Zhang et al., 2023). In this study, we used immunofluorescence to localize the constituent proteins of the PANoptosome, revealing that KAE reduced the binding of Caspase-8 to RIPK3 and ASC in RAW264.7 cells, which suggested that KAE inhibits the activation and assembly of the PANoptosome. Therefore, KAE may exert its effects by attenuating the inflammatory response and tissue damage associated with PANoptosis, thereby ameliorating ALI symptoms.

In cells exposed to injury, oxidative stress, or inflammation, mitochondria can exhibit functional abnormalities or structural damage. This results in the loss of MMP, changes in mitochondrial permeability, and the release of mitochondrial DNA into the cytoplasm (Jeon et al., 2021; Lee et al., 2022; Ma et al., 2022). These cytoplasmic DNA fragments are recognized as pathogenic features or DAMPs by receptors such as cGAS and ZBP1, which activate the cGAS-STING pathway leading to PANoptosis (Domizio et al., 2022; Shi et al., 2024). Here, we found that LPS-induced cellular metabolic injury led to an increase in ROS, a decrease in MMP, and a significant accumulation of cytoplasmic dsDNA. Treatment with KAE restored the MMP and significantly reduced the accumulation of cytoplasmic dsDNA, indicating that intracytoplasmic DNA activates the cGAS-STING pathway, causing PANoptosis in ALI. The ability of KAE to mitigate these effects confirms its therapeutic potential in ALI.

Our finding that treatment with KAE significantly ameliorated LPS-induced ALI by inhibiting the activation of the cGAS-STING pathway is of great scientific and clinical importance. The cGAS-STING pathway plays a key role in the complex pathogenesis of ALI, which mainly involves an excessive inflammatory response, oxidative stress, and cell death (Chen et al., 2024). Our experiments have shown that KAE inhibits the overactivation of the cGAS-STING pathway by reducing the release of cytoplasmic DNA, thereby attenuating the infiltration of inflammatory cells and reducing cell death. This finding provides a new targeting strategy for the treatment of ALI. Compared with existing ALI treatments, such as mechanical ventilation and anti-inflammatory drugs, KAE, as a natural product, has fewer side effects and broad therapeutic potential.

The possibility that the KAE-mediated prevention of LPS-induced macrophage death increases the number of macrophages contributing to ALI requires further study. Macrophages have a dual role in the development of ALI, acting as the first, innate line of defense against organ damage and infection through the removal of pathogens and cellular debris, and potentially exacerbating lung injury through the release of excessive inflammatory factors (Amo-Aparicio et al., 2021; Zhang et al., 2023). Therefore, in this study, we sought to administer KAE 2 h before ALI induction in mice, which effectively reduced lung injury and controlled macrophage overactivation and inflammatory factor release. Indeed, KAE is known to reduce inflammation and death of macrophages, thereby improving the body’s defenses. In addition, while mesenchymal stem cell-derived exosomes have been shown to inhibit Caspase-1 activation and reduce macrophage pyroptosis, thereby attenuating ALI (Liu, 2021), triptolide was found to induce PANoptosis in macrophages, leading to multi-organ damage in mice (Zhang et al., 2023). These findings suggest that reducing macrophage death may be a beneficial therapeutic strategy. However, the specific effects of KAE on regulation of macrophage function and homeostasis of the immune response need to be further clarified in future studies. Strategies aimed at treating ALI by reducing macrophage PANoptosis deserve further research and exploration.

## Conclusion

Our study findings indicate that KAE mitigates LPS-induced ALI by inhibiting the activation of the cGAS-STING signaling pathway. This inhibition reduces the release of intracytoplasmic DNA, subsequently decreasing PANoptosis and attenuating inflammatory responses.

## Data Availability

The original contributions presented in the study are included in the article/Supplementary Material, further inquiries can be directed to the corresponding author.
