# Supplementary material for: KAE ameliorates LPS-mediated acute lung injury by inhibiting PANoptosis through the intracellular DNA-cGAS-STING axis
Source: Front Pharmacol. 2025 Jan 7;15:1461931. doi: 10.3389/fphar.2024.1461931 (PMC11747328; doi:10.3389/fphar.2024.1461931)
Supplement: Supplementary file 1 [file DataSheet2.pdf]

Animals-western blot-densitometr

		Repeat 1		Repeat 2		Repeat 3		Repeat 1		Repeat 2		Repeat 3					
Caspase1	1	3176.983	1	3863.054	1	5545.418	1	25135.66	1	30013.13	1	31326.54		0.126393	0.128712	0.17702	
	2	35258.39	2	41093.92	2	39809.39	2	31381.42	2	38405.95	2	39626.54		1.123543	1.069988	1.004614	
	3	26829.92	3	29734.8	3	23450.75	3	32637.97	3	38527.75	3	39673.63		0.822046	0.771776	0.591092	
	4	10913.59	4	12865	4	14092.56	4	26347.25	4	31864.49	4	33359.32		0.414221	0.403741	0.422447	
GSDMD	4	19981.29	1	23477.07	1	22492.24	1	10166.1	1	12570.77	1	11512.05		1.965483	1.867591	1.9538	
	5	23425.22	2	26284.7	2	25220.17	2	7061.56	2	10190.77	2	8819.095		3.317286	2.579265	2.859723	
	6	37090.48	3	38921.31	3	37615.19	3	28540.7	3	30595.97	3	29565.65		1.299564	1.272106	1.27226	
	7	30503.53	4	32276.65	4	31174.24	4	32568.29	4	33618.7	4	32968.41		0.936602	0.96008	0.945579	
Caspase-8	1	96.899	1	93.192	1	43.364	1	23871.8	1	24440.22	1	24728.97		0.004059	0.003813	0.001754	
	2	29644.39	2	30514.56	2	26084.02	2	22477.68	2	23011.92	2	23282.1		1.318837	1.326033	1.120347	
	3	22632.46	3	24342.92	3	21784.63	3	22796.9	3	23305.9	3	23605.08		0.992787	1.044496	0.922879	
	4	4961.024	4	5121.024	4	3546.196	4	33522.39	4	34003.1	4	34480.8		0.147991	0.150605	0.102846	
Caspase-3	1	14789.75	1	16043	1	4932.288	1	39665.88	1	39660.88	1	37874.47		0.372858	0.404504	0.130227	
	2	38781.14	2	39645.14	2	34958.24	2	45726.76	2	45803.4	2	44511.64		0.848106	0.86555	0.785373	
	3	19861.31	3	20617.43	3	11415.68	3	43825.64	3	43970.1	3	42799.23		0.453189	0.468897	0.266726	
	4	10868.02	4	11449.77	4	3617.276	4	45911.3	4	46101.47	4	44874.88		0.236718	0.24836	0.080608	
MLKL	1	11354.36	1	11759.65	1	7893.409	1	29123.34	1	26788.68	1	28078.39		0.389871	0.438978	0.28112	
	2	31742.22	2	32015.75	2	31521.63	2	29200.9	2	27093.37	2	28263.95		1.087029	1.181682	1.115259	
	3	18084.05	3	18480.17	3	16417.39	3	25561.97	3	23111.15	3	24505.85		0.707459	0.799621	0.669937	
	4	15993.34	4	16363.92	4	14091.8	4	27858.49	4	25576.78	4	26881.9		0.574092	0.639796	0.524212	
cGAS	1	11661.37	1	12151.37	1	15951.2	1	36812.42	0.316778	1	0.330089	1	0.43331	1	1	1	1
	2	34624.51	2	34660.51	2	39001.27	2	34571.56	1.001532	3.161619	1.002573	3.037282	1.128132	2.603519	3.161619	3.037282	2.603519
	3	28466.61	3	28602.08	3	29572.66	3	36986.32	0.769652	2.429626	0.773315	2.342748	0.799557	1.84523	2.429626	2.342748	1.84523
	4	11491.71	4	11699.71	4	13123.3	4	36043.7	0.318827	1.006468	0.324598	0.983365	0.364094	0.840262	1.006468	0.983365	0.840262

	1	11317.63	1	7823.853	1	9913.217	1	37152.42	0.304627	1	0.210588	1	0.266826	1	1	1	1
STING	2	34768.85	2	34352.15	2	34633.44	2	34763.85	1.000144	3.283175	0.988157	4.692371	0.996249	3.733707	3.283175	4.692371	3.733707
	3	26729.27	3	24165.9	3	26523.44	3	37241.61	0.717726	2.356081	0.648895	3.08135	0.712199	2.669155	2.356081	3.08135	2.669155
	4	9140.66	4	7264.539	4	8420.711	4	36277.29	0.251966	0.827131	0.20025	0.950911	0.232121	0.869934	0.827131	0.950911	0.869934
	1	15621.9	1	14623.2	1	15829.73	1	34535.3	0.452346	1	0.423428	1	0.458364	1	1	1	1
p-TBK1	2	27963.46	2	27925.92	2	27663.58	2	32913.56	0.849603	1.878215	0.848463	2.003796	0.840492	1.833678	1.878215	2.003796	1.833678
	3	16333.63	3	14883.75	3	16017.75	3	34959.59	0.467215	1.03287	0.425742	1.005465	0.458179	0.999597	1.03287	1.005465	0.999597
	4	12707.05	4	9592.125	4	13042.29	4	41065.32	0.309435	0.684067	0.233582	0.551646	0.317599	0.692896	0.684067	0.551646	0.692896
	1	8670.933	1	7216.104	1	6610.276	1	37499.54	0.231228	1	0.192432	1	0.176276	1	1	1	1
NF-KB	2	30129.56	2	29383.2	2	29323.9	2	35256.51	0.854581	3.695843	0.833412	4.330947	0.83173	4.718335	3.695843	4.330947	4.718335
	3	25865.56	3	24138.32	3	23392.61	3	37836.9	0.683607	2.956422	0.637957	3.315237	0.618249	3.507272	2.956422	3.315237	3.507272
	4	6128.104	4	3980.276	4	3152.912	4	36866.87	0.166223	0.718869	0.107963	0.561048	0.085522	0.485157	0.718869	0.561048	0.485157
	1	18007.8	1	16105.68	1	20889.41	1	38515.34	0.467549	1	0.418163	1	0.542366	1	1	1	1
NLRP3	2	34549.75	2	33769.75	2	36068.02	2	38886.22	0.888483	1.9003	0.868425	2.076762	0.927527	1.71015	1.9003	2.076762	1.71015
	3	21911.78	3	20832.25	3	23803.73	3	35319.02	0.620396	1.326912	0.589831	1.410529	0.673963	1.242636	1.326912	1.410529	1.242636
	4	923.134	4	380.749	4	1834.447	4	36663	0.025179	0.053853	0.010385	0.024835	0.050035	0.092254	0.053853	0.024835	0.092254
	1	2614.811	1	2690.104	1	1958.326	1	37898.8	0.068995	1	0.070981	1	0.051673	1	1	1	1
ASC	2	33347.2	2	33492.2	2	33074.61	2	38389.44	0.868655	12.5902	0.872433	12.29103	0.861555	16.67337	12.5902	12.29103	16.67337
	3	12402.51	3	12757.22	3	11117.97	3	34409.54	0.360438	5.224153	0.370747	5.223162	0.323107	6.252984	5.224153	5.223162	6.252984
	4	6213.539	4	6371.539	4	5456.589	4	34288.8	0.181212	2.626467	0.18582	2.617872	0.159136	3.079707	2.626467	2.617872	3.079707
	1	10826.08	1	9110.66	1	10463.37	1	39188.7	0.276255	1	0.232482	1	0.267	1	1	1	1
ZBP1	2	32941.8	2	32413.61	2	32834.85	2	39821.51	0.827236	2.994467	0.813972	3.501231	0.824551	3.088209	2.994467	3.501231	3.088209
	3	23671.73	3	22597.49	3	23449.9	3	39059.46	0.606043	2.193783	0.578541	2.488542	0.600364	2.248559	2.193783	2.488542	2.248559
	4	3087.933	4	1317.619	4	2624.589	4	38442.22	0.080327	0.29077	0.034275	0.147432	0.068274	0.255707	0.29077	0.147432	0.255707

Cell-western blot-densitometr

		Repeat 1		Repeat 2		Repeat 3		Repeat 1		Repeat 2		Repeat 3					
Caspase1	1	10733.66	1	9197.539	1	11156.49	1	14965.08	1	12376.54	1	15532.9		0.717247	0.743143	0.718249	
	2	27746.8	2	28328.1	2	28072.92	2	23598.68	2	24517.8	2	29220.75		1.175778	1.155409	0.960719	
	3	7780.146	3	3614.66	3	8536.56	3	25442.75	3	20673.75	3	26336.34		0.30579	0.174843	0.324136	
	4	7277.903	4	3546.711	4	7948.903	4	36393.34	4	36925.61	4	36573.8		0.199979	0.09605	0.217339	
GSDMD	1	2687.146	1	2781.439	1	2716.903	1	19859.77	1	21858.95	1	21551.77		0.135306	0.127245	0.126064	
	2	32102.7	2	32603.7	2	32206.58	2	35630.36	2	40378.19	2	40349.77		0.900993	0.807458	0.798185	
	3	6176.095	3	6473.338	3	6294.217	3	26130.29	3	29059.65	3	28848.95		0.236358	0.22276	0.218178	
	4	7863.459	4	8051.116	4	7978.045	4	19927.1	4	21961.85	4	21579.8		0.394611	0.366595	0.3697	
Caspase-8	1	25381.58	1	24255.46	1	26862.29	1	31018.39	1	30281.73	1	31462.1		0.818275	0.800993	0.853798	
	2	36073.72	2	34717.6	2	36470.43	2	31790.29	2	30123.58	2	31383.58		1.13474	1.152506	1.162086	
	3	20391.46	3	18599.63	3	21885.65	3	30349.15	3	29215.15	3	30279.85		0.671896	0.636643	0.722779	
	4	5307.631	4	3721.51	4	6878.752	4	29924.27	4	28759.73	4	30099.56		0.177369	0.1294	0.228533	
Caspase-3	1	4340.054	1	3507.397	1	4431.933	1	31704.95	1	31818.13	1	31397.59		0.136889	0.110233	0.141155	
	2	27939.97	2	29316.32	2	27542.32	2	26134.52	2	25795.1	2	25358.28		1.069083	1.136507	1.086127	
	3	33896.92	3	34559.22	3	34450.1	3	39875.46	3	39494.05	3	38889.63		0.85007	0.875049	0.885843	
	4	5249.761	4	4558.175	4	5505.539	4	28184.3	4	27994.05	4	27496.18		0.186265	0.162827	0.200229	
MLKL	1	14519.25	1	13273.95	1	14650.66	1	41904.79	4	42214.92	1	33150.55		0.346482	0.314438	0.441943	
	2	37468.17	2	36431.58	2	37497.87	2	32761.6	5	32512.77	2	21291.97		1.143661	1.120531	1.761127	
	3	16712.78	3	15084.83	3	16702.49	3	30964.84	6	30840.67	3	21171.15		0.539734	0.489121	0.788927	
	4	16253.97	4	13826.56	4	16072.68	4	24031.24	7	23987.53	4	21124.97		0.676369	0.576406	0.760838	
cGAS	1	11326.08	1	9134.832	1	10135.83	1	38270.78	0.295946	1	0.238689	1	0.264845	1	1	1	1
	2	30567.05	2	30067.1	2	30540.68	2	36630.25	0.834476	2.819691	0.820827	3.438891	0.833756	3.148088	2.819691	3.099577	3.148088
	3	24882.1	3	24250.97	3	24619.27	3	40283.95	0.617668	2.087098	0.602001	2.522109	0.611143	2.307549	2.087098	2.273254	2.307549
	4	17013.22	4	15695.39	4	16060.15	4	35279.3	0.482244	1.6295	0.444889	1.863884	0.455229	1.718848	1.6295	1.679975	1.718848

ZBP1	1	10103.17	1	8014.267	1	6370.731	1	30390.58	0.332444	1	0.263709	1	0.209628	1	1	1	1
	2	38332.72	2	36301.48	2	34976.36	2	26590.97	1.441569	4.336276	1.365181	5.176846	1.315347	6.274658	4.336276	5.176846	6.274658
	3	24776	3	21923.41	3	19753.46	3	31696.46	0.781664	2.351266	0.691667	2.622844	0.623207	2.972913	2.351266	2.622844	2.972913
	4	7550.853	4	5796.782	4	4607.903	4	29880.39	0.252703	0.760136	0.194	0.735658	0.154212	0.735643	0.760136	0.735658	0.735643
STING	1	11897.15	1	10390.61	4	10924.15	1	38268.49	0.310886	1	0.271519	1	0.285461	1	1	1	1
	2	28259.02	2	28392.56	5	28114.85	2	36772.54	0.768482	2.471907	0.772113	2.843683	0.764561	2.678342	2.471907	2.843683	2.678342
	3	10351.37	3	9499.246	6	10358.08	3	40153.9	0.257792	0.829218	0.236571	0.871288	0.257959	0.90366	0.829218	0.871288	0.90366
	4	11825.2	4	11549.78	7	11830.95	4	35124.88	0.336662	1.082909	0.328821	1.211042	0.336825	1.179937	1.082909	1.211042	1.179937
p-TBK1	1	3100	1	3000	1	4178.012	1	34535.3	0.089763	1	0.086868	1	0.120978	1	1	1	1
	1	33059.44	4	33076.44	2	36263.47	2	32913.56	1.004432	11.18979	1.004949	11.56873	1.101779	9.107265	11.18979	11.56873	9.107265
	2	28840.1	5	28992.87	3	22801.09	3	34959.59	0.824955	9.190346	0.829325	9.546999	0.652213	5.391168	9.190346	9.546999	5.391168
	3	21118.92	6	24752.82	4	24340	4	41065.32	0.514276	5.729254	0.602767	6.938914	0.592714	4.899356	5.729254	6.938914	4.899356
NF-KB	1	7829.296	1	6073.276	1	5780.054	1	33746.68	0.232002	1	0.179967	1	0.171278	1	1	1	1
	2	38615.58	2	36914.29	2	36177.17	2	31701.49	1.2181	5.250386	1.164434	6.470277	1.141182	6.662758	5.250386	6.470277	6.662758
	3	11597.13	3	8896.468	3	8209.64	3	32391.1	0.358034	1.543239	0.274658	1.52616	0.253454	1.479782	1.543239	1.52616	1.479782
	4	7281.539	4	5539.175	4	5140.468	4	32331.23	0.225217	0.970754	0.171326	0.951987	0.158994	0.928281	0.970754	0.951987	0.928281
NLRP3	1	3852.962	1	3891.255	1	3743.255	1	29846.3	0.129093	1	0.130376	1	0.125418	1	1	1	1
	2	34973.56	2	35030.85	2	34311.85	2	33544.39	1.042605	8.07636	1.044313	8.009983	1.022879	8.155777	8.07636	8.072216	8.155777
	3	21211.56	3	20983.78	3	20940.73	3	38883.97	0.545509	4.22569	0.539651	4.139176	0.538544	4.294002	4.22569	4.171335	4.294002
	4	26372.41	4	26207.58	4	26259.17	4	35495.32	0.742983	5.755386	0.738339	5.663131	0.739792	5.898627	5.755386	5.707131	5.898627
ASC	1	33625.34	1	32196.1	1	31522.68	1	32792.05	1.025411	1	0.981826	1	0.96129	1	1	1	1
	2	42222.6	2	41536.95	2	41816.92	2	29793.9	1.417156	1.382036	1.394142	1.419948	1.40354	1.460058	1.382036	1.419948	1.460058
	3	28137.46	3	26194.63	3	24331.02	3	34947.85	0.805127	0.785175	0.749535	0.763409	0.696209	0.724245	0.785175	0.763409	0.724245
	4	15283.73	4	12866.37	4	12468.47	4	33608.83	0.454753	0.443484	0.382827	0.389913	0.370988	0.385927	0.443484	0.389913	0.385927
