# Supplementary figures and images for: KAE ameliorates LPS-mediated acute lung injury by inhibiting PANoptosis through the intracellular DNA-cGAS-STING axis
Source: Front Pharmacol. 2025 Jan 7;15:1461931. doi: 10.3389/fphar.2024.1461931 (PMC11747328; doi:10.3389/fphar.2024.1461931)

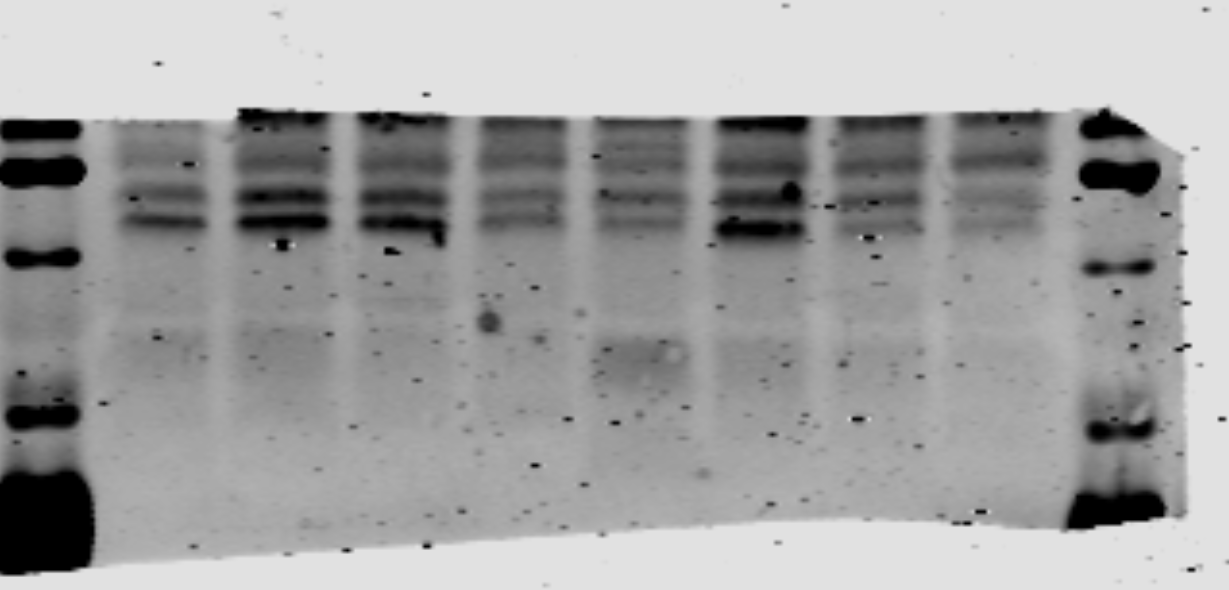

Supplement: Supplementary file 2 [file DataSheet1.zip › Supplementary Information/animals-Western blot/ASC/ASC 2.tif]

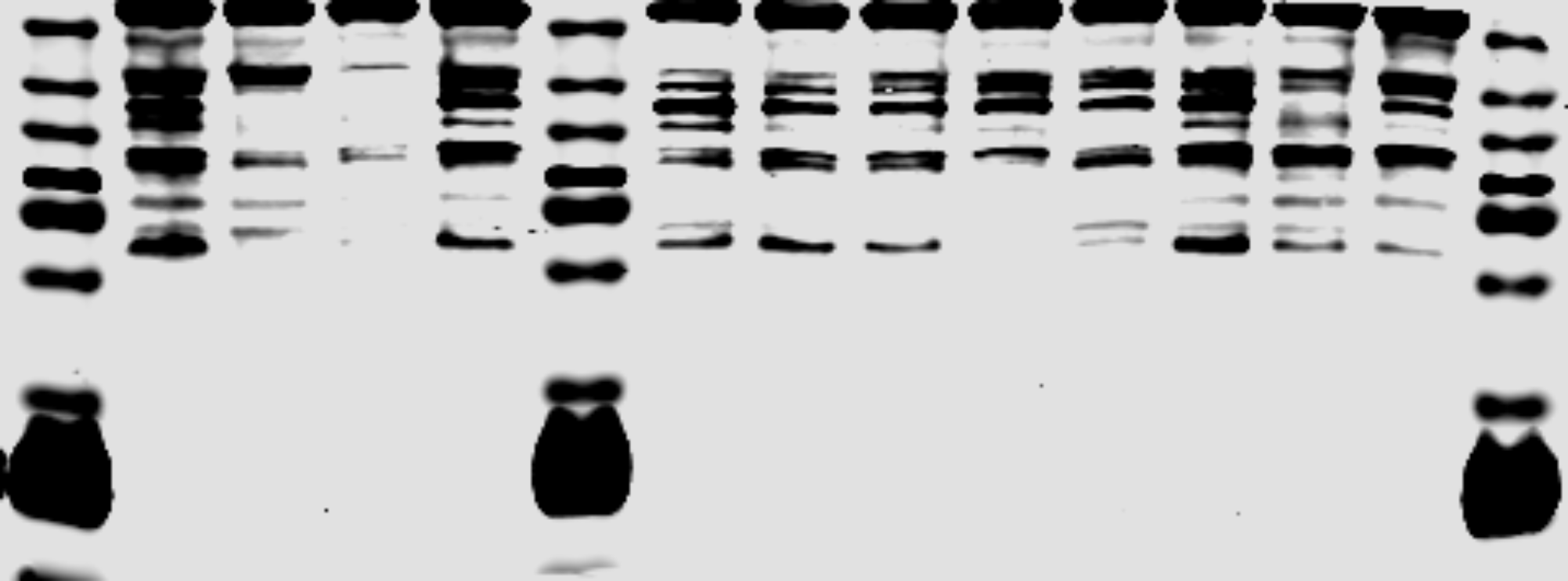

Supplement: Supplementary file 2 [file DataSheet1.zip › Supplementary Information/animals-Western blot/ASC/asc 1.tif]

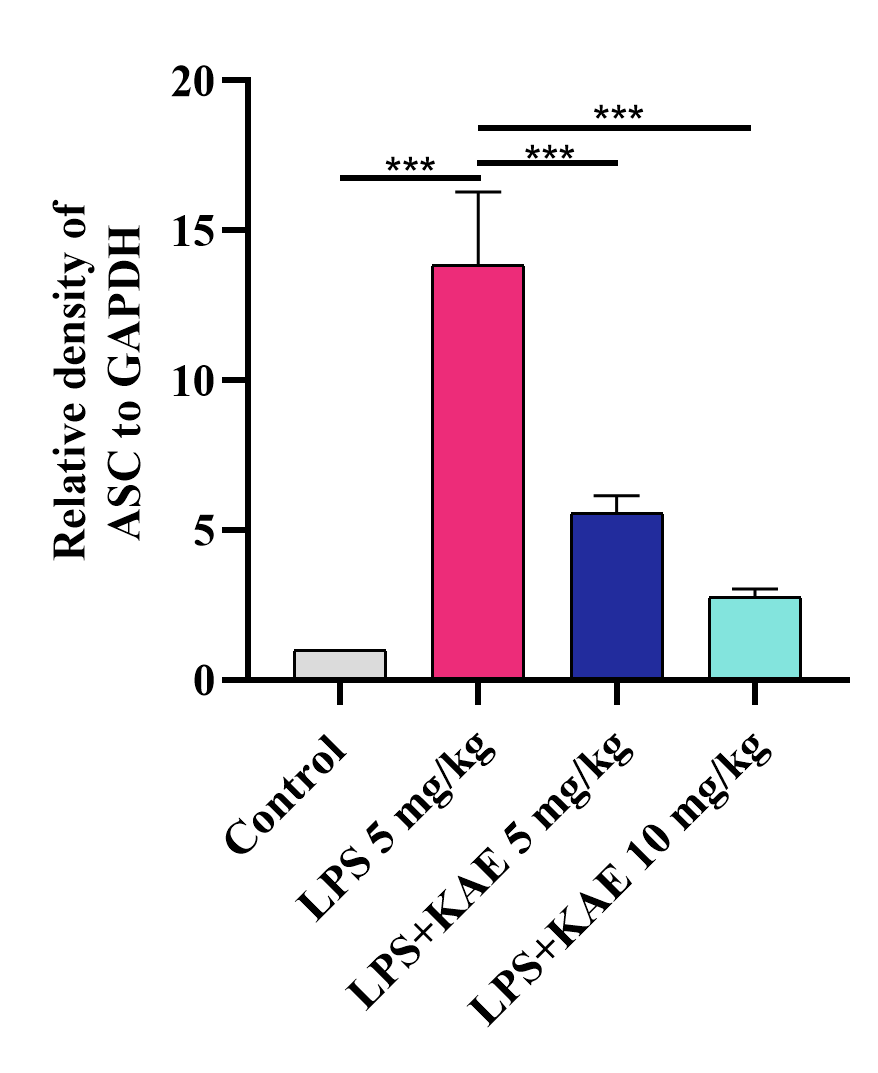

Supplement: Supplementary file 2 [file DataSheet1.zip › Supplementary Information/animals-Western blot/ASC.tif]

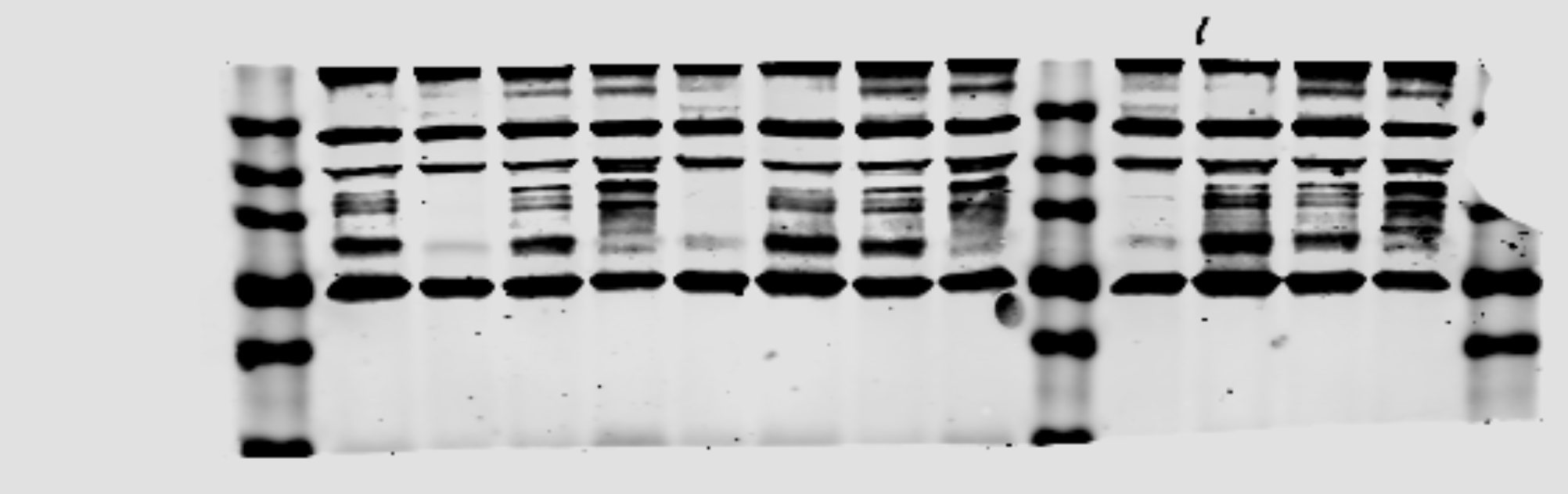

Supplement: Supplementary file 2 [file DataSheet1.zip › Supplementary Information/animals-Western blot/CASP1/CAS-1-1.tif]

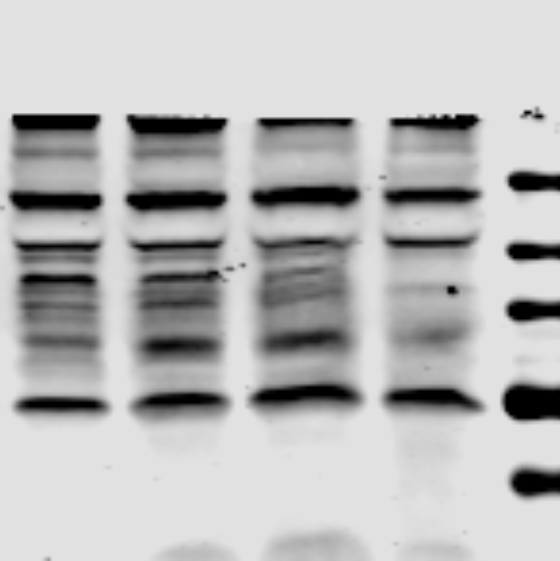

Supplement: Supplementary file 2 [file DataSheet1.zip › Supplementary Information/animals-Western blot/CASP3/CASP 3-1.tif]

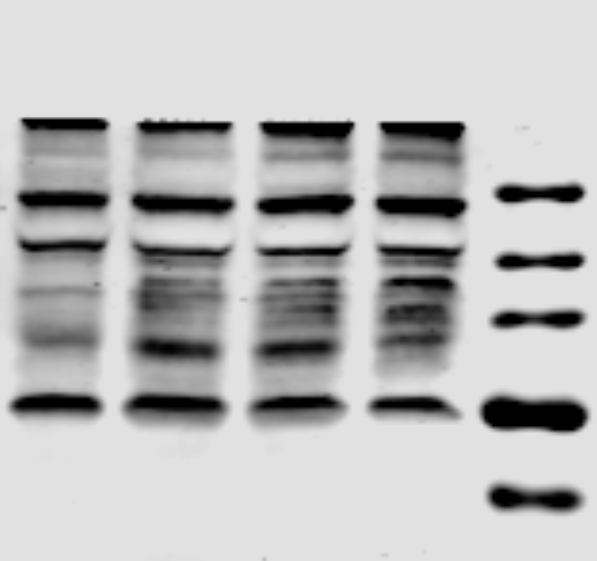

Supplement: Supplementary file 2 [file DataSheet1.zip › Supplementary Information/animals-Western blot/CASP3/CASP 3-2.tif]

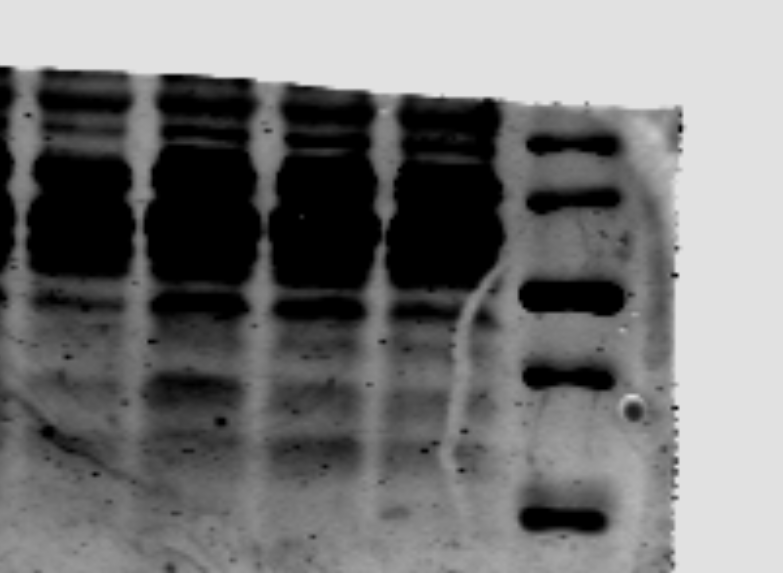

Supplement: Supplementary file 2 [file DataSheet1.zip › Supplementary Information/animals-Western blot/CASP3/casp 3..tif]

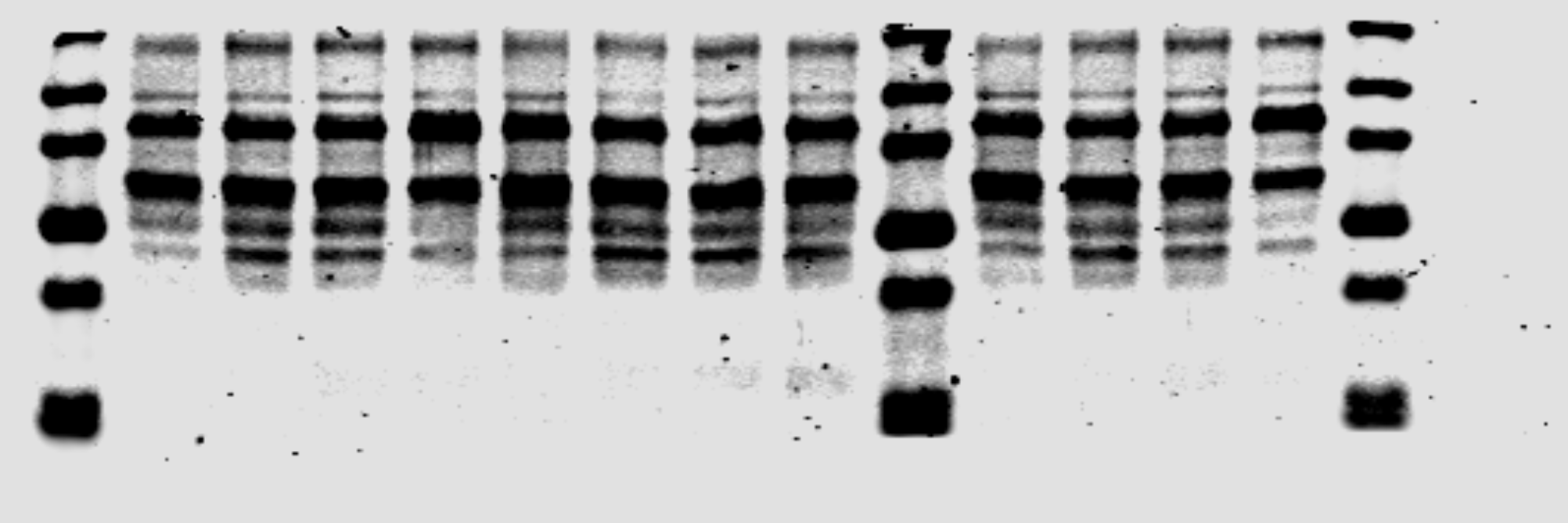

Supplement: Supplementary file 2 [file DataSheet1.zip › Supplementary Information/animals-Western blot/CASP8/casps8-1.tif]

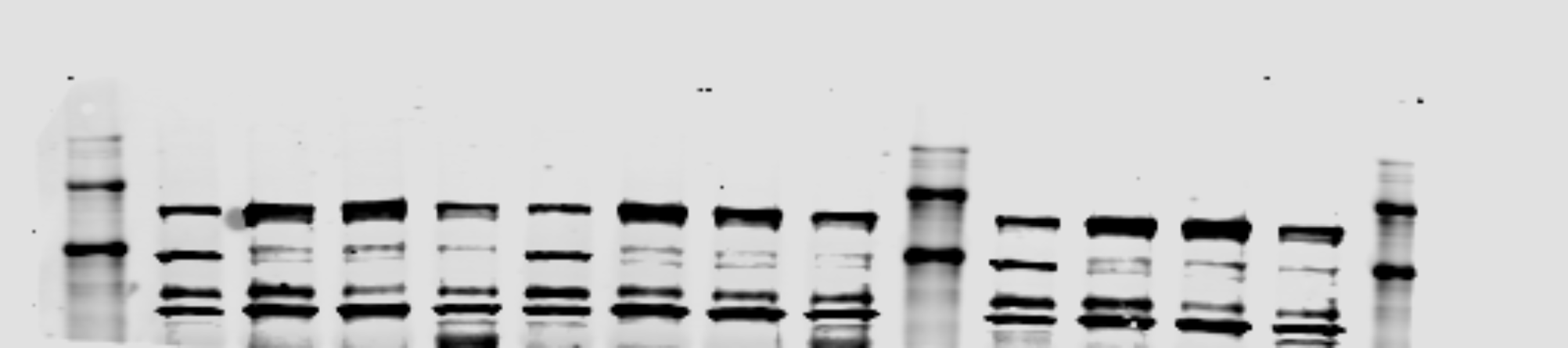

Supplement: Supplementary file 2 [file DataSheet1.zip › Supplementary Information/animals-Western blot/CGAS/CGAS 1.tif]

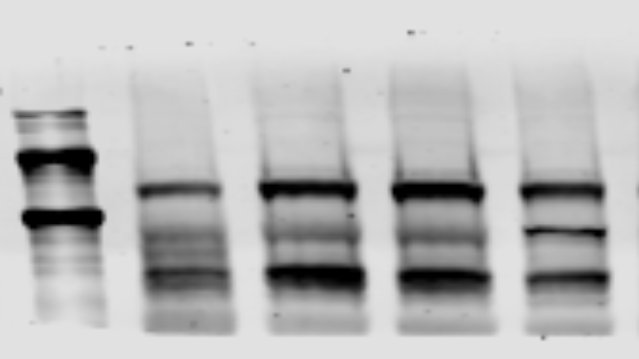

Supplement: Supplementary file 2 [file DataSheet1.zip › Supplementary Information/animals-Western blot/CGAS/CGAS 2.tif]

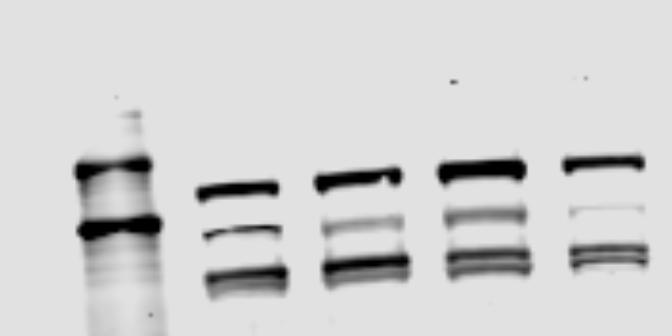

Supplement: Supplementary file 2 [file DataSheet1.zip › Supplementary Information/animals-Western blot/CGAS/cgas 3.tif]

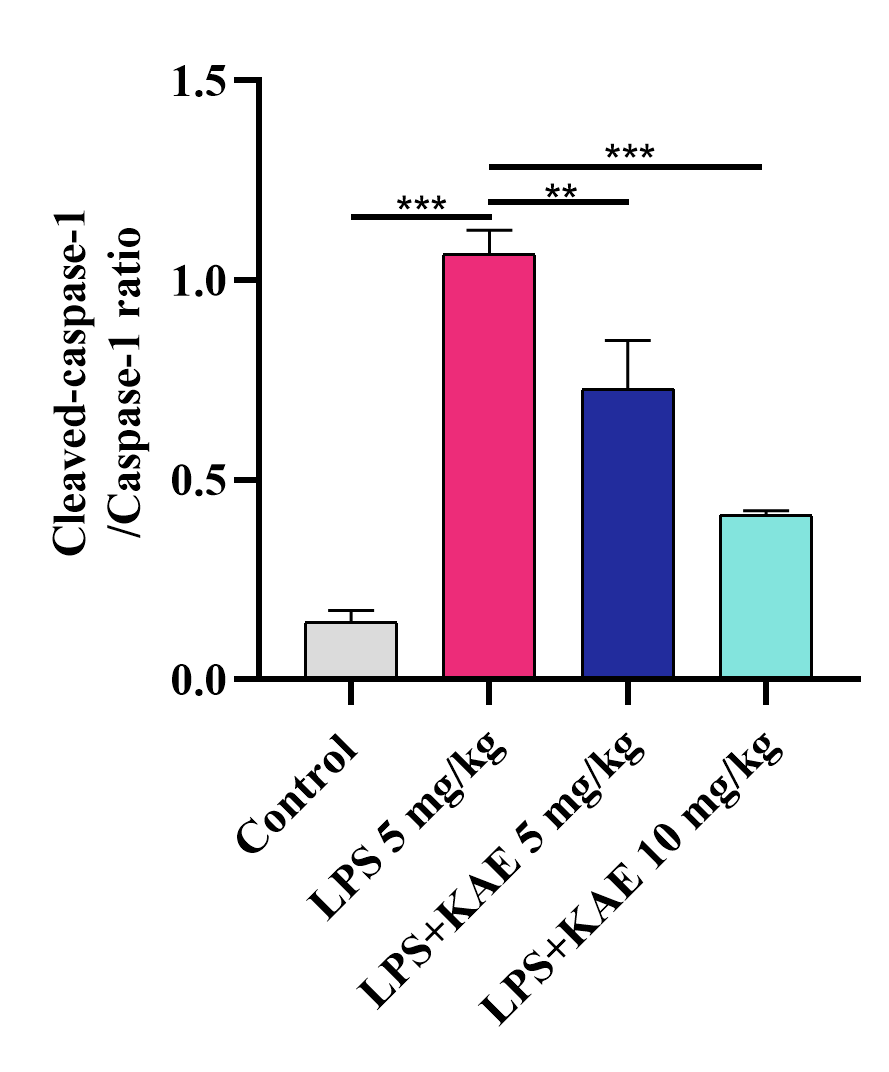

Supplement: Supplementary file 2 [file DataSheet1.zip › Supplementary Information/animals-Western blot/Caspase-1.tif]

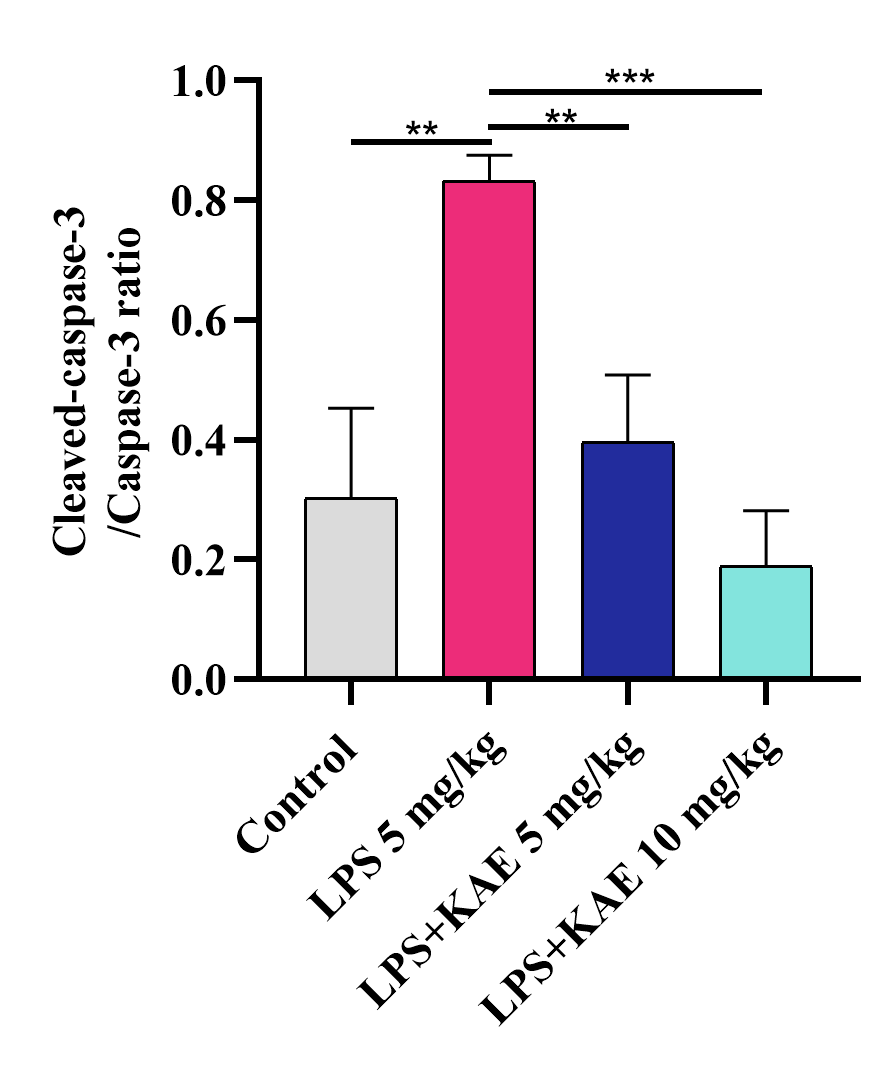

Supplement: Supplementary file 2 [file DataSheet1.zip › Supplementary Information/animals-Western blot/Caspase-3.tif]

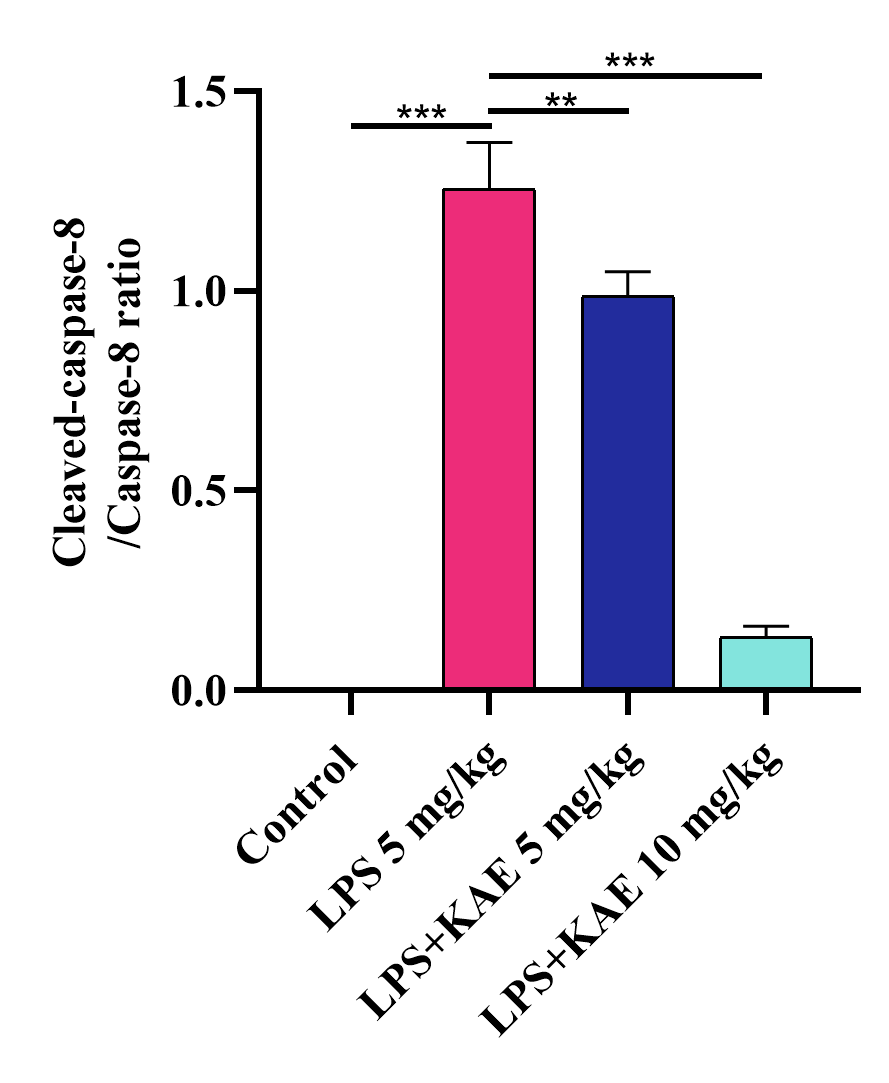

Supplement: Supplementary file 2 [file DataSheet1.zip › Supplementary Information/animals-Western blot/Caspase-8.tif]

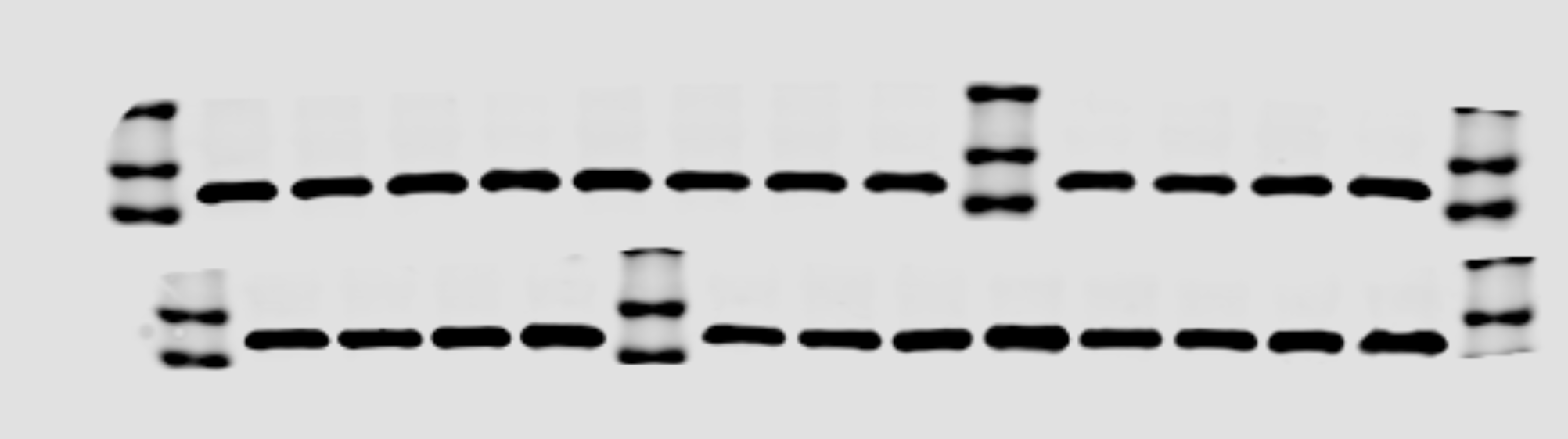

Supplement: Supplementary file 2 [file DataSheet1.zip › Supplementary Information/animals-Western blot/GAPDH/gapdh 1.tif]

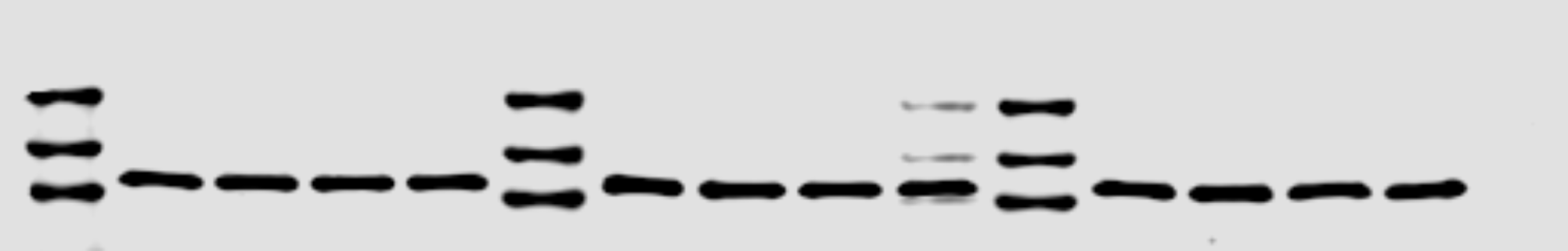

Supplement: Supplementary file 2 [file DataSheet1.zip › Supplementary Information/animals-Western blot/GAPDH/gapdh..tif]

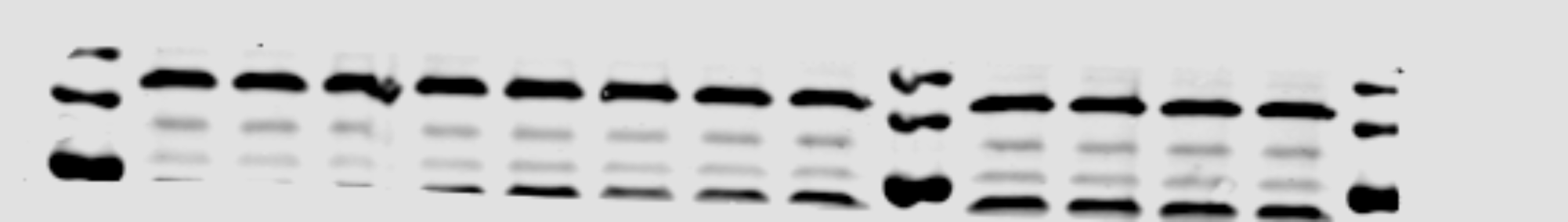

Supplement: Supplementary file 2 [file DataSheet1.zip › Supplementary Information/animals-Western blot/GAPDH/gapdh.tif]

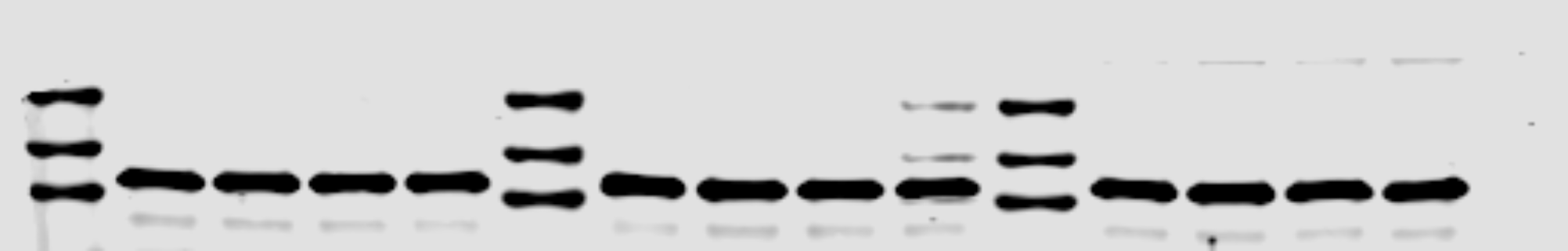

Supplement: Supplementary file 2 [file DataSheet1.zip › Supplementary Information/animals-Western blot/GAPDH/gapdh1.tif]

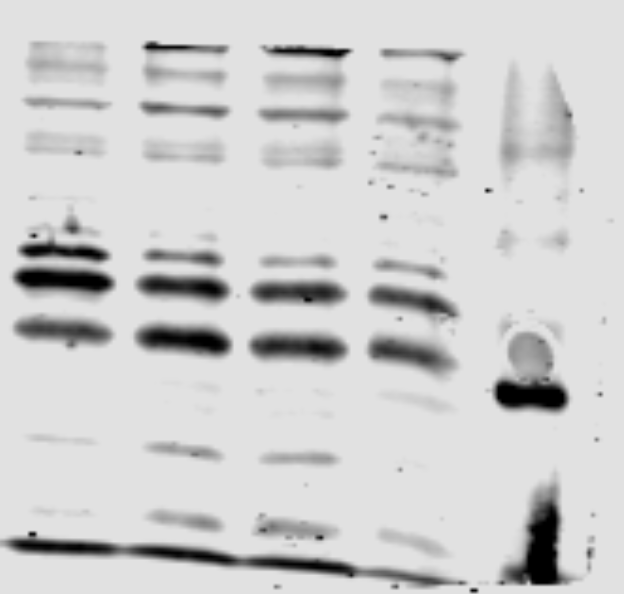

Supplement: Supplementary file 2 [file DataSheet1.zip › Supplementary Information/animals-Western blot/GSDMD/GSDMD 1.tif]

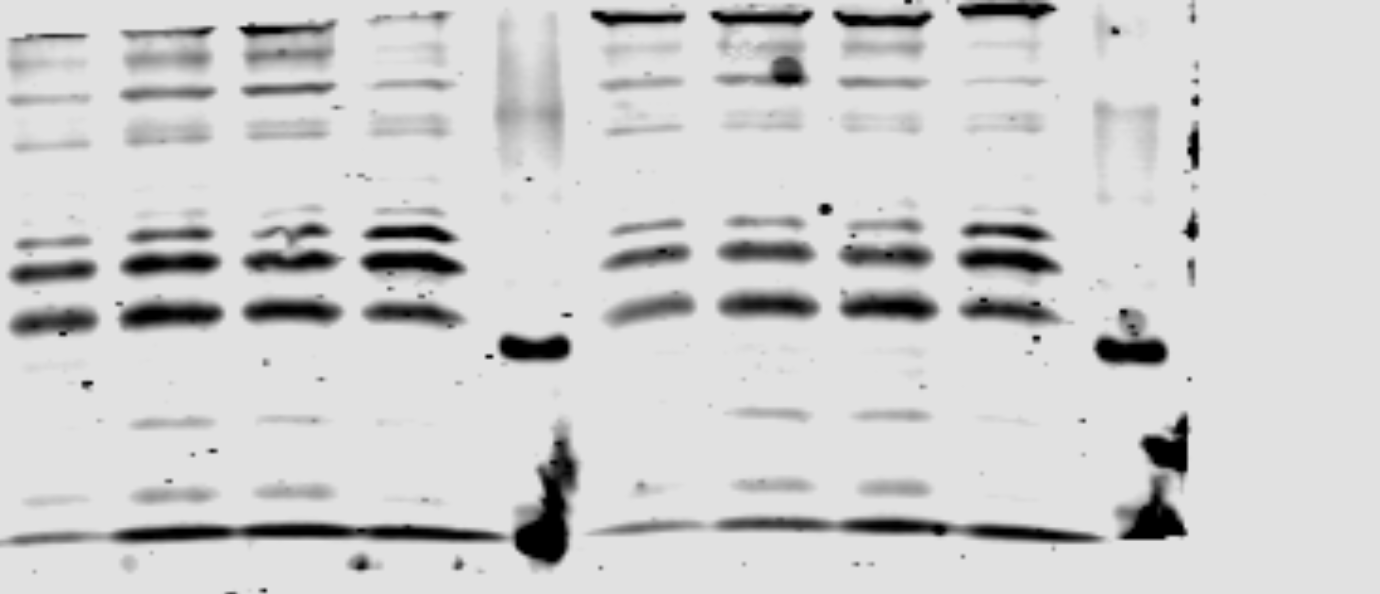

Supplement: Supplementary file 2 [file DataSheet1.zip › Supplementary Information/animals-Western blot/GSDMD/GSDMD 2.tif]

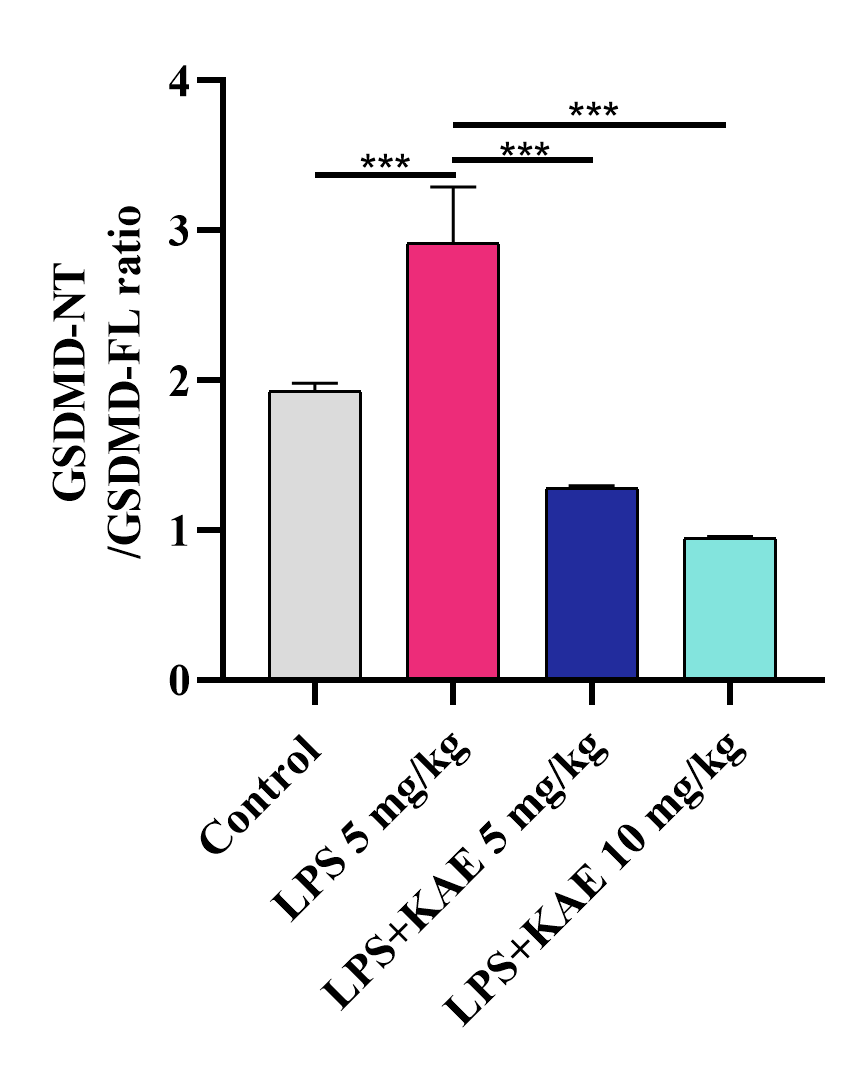

Supplement: Supplementary file 2 [file DataSheet1.zip › Supplementary Information/animals-Western blot/GSDMD.tif]

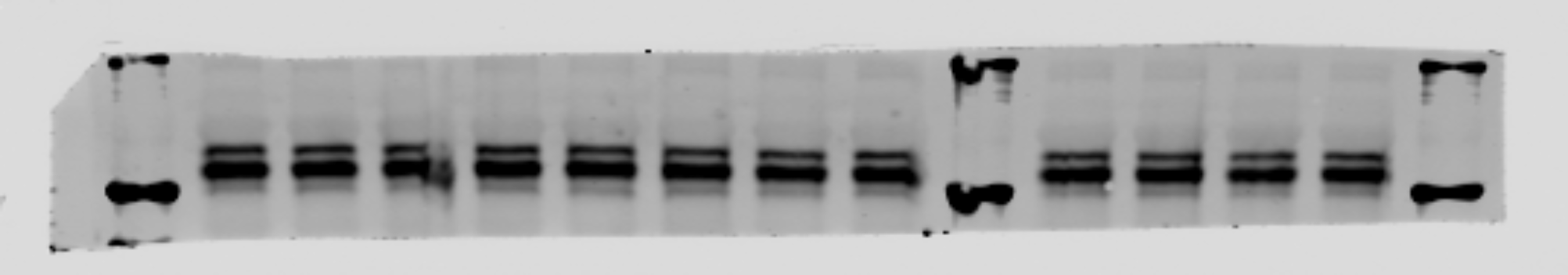

Supplement: Supplementary file 2 [file DataSheet1.zip › Supplementary Information/animals-Western blot/MLKL/mlkl .tif]

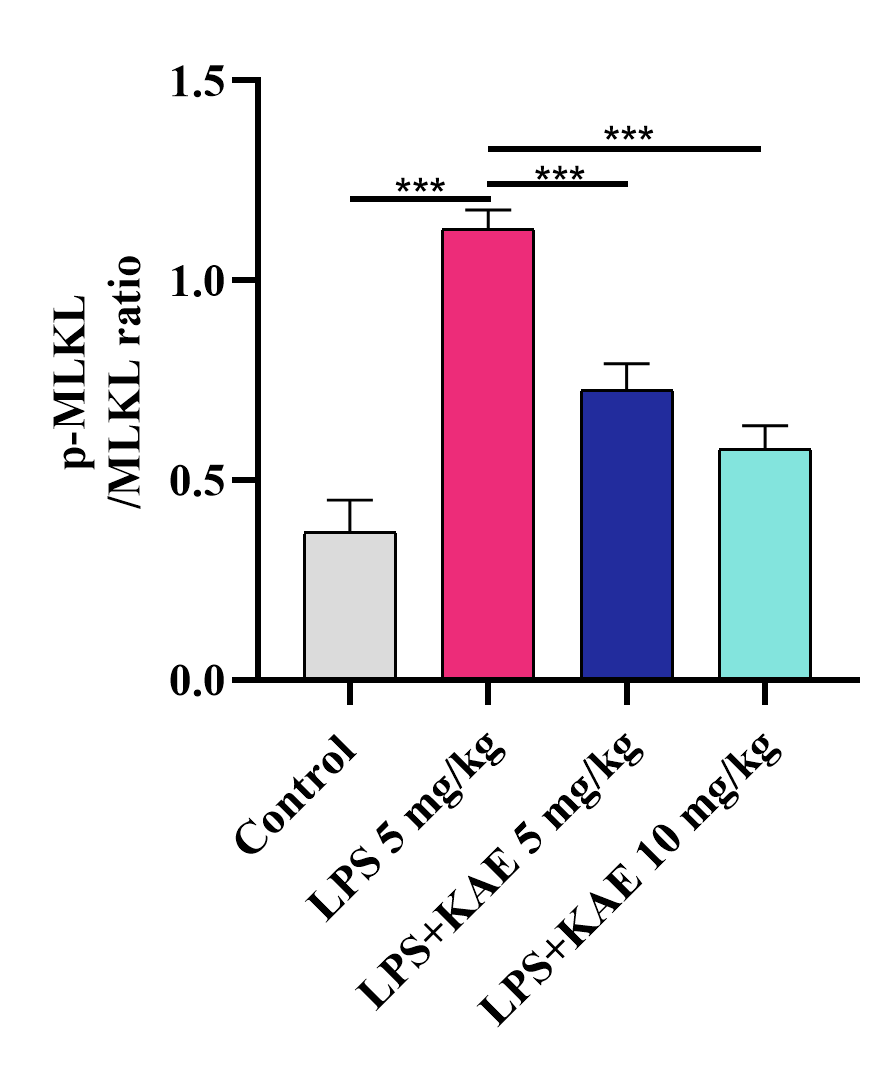

Supplement: Supplementary file 2 [file DataSheet1.zip › Supplementary Information/animals-Western blot/MLKL.tif]

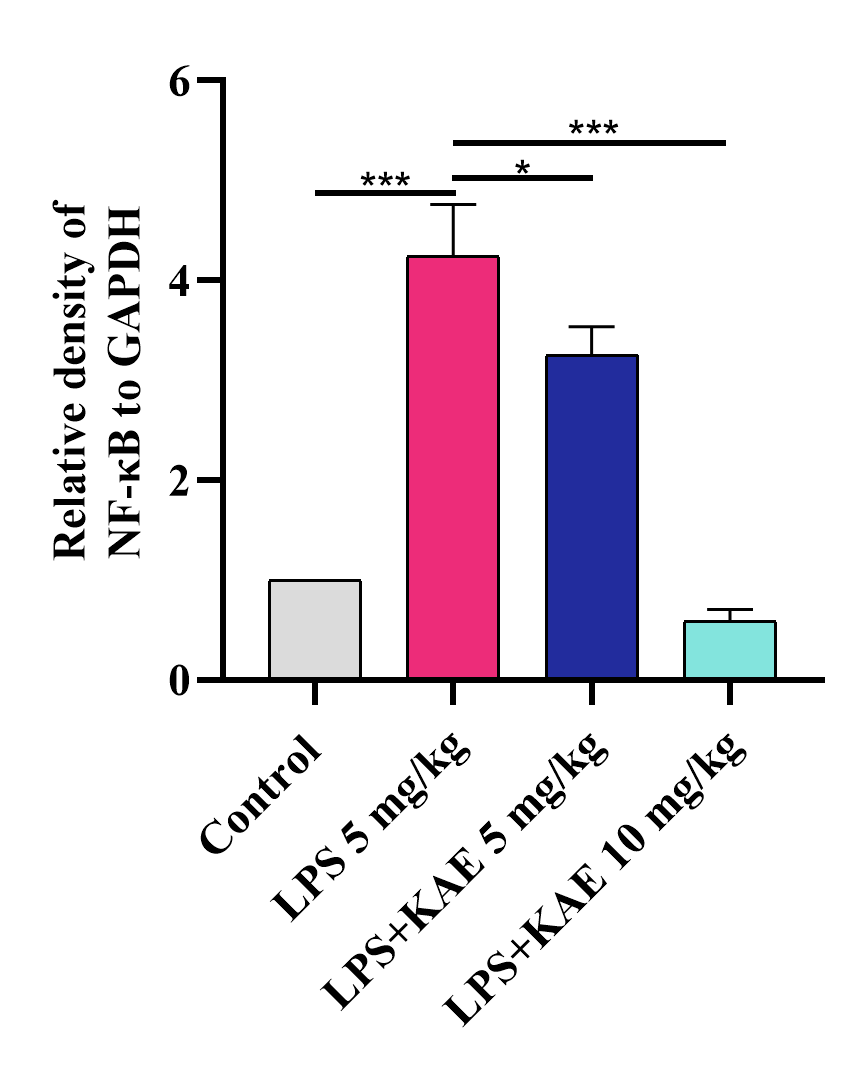

Supplement: Supplementary file 2 [file DataSheet1.zip › Supplementary Information/animals-Western blot/NF-KB.tif]

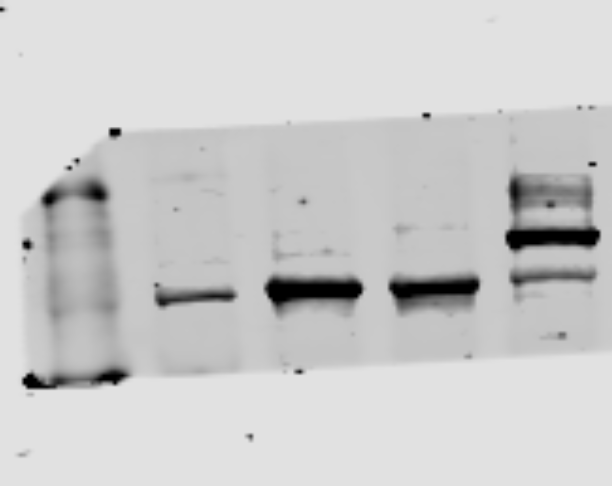

Supplement: Supplementary file 2 [file DataSheet1.zip › Supplementary Information/animals-Western blot/NFKB/NF-KB 1.tif]

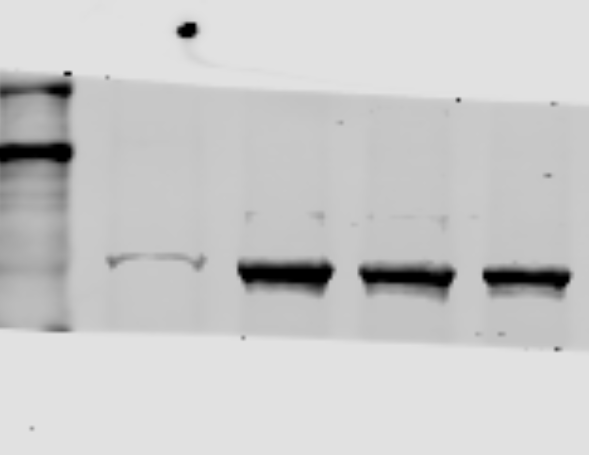

Supplement: Supplementary file 2 [file DataSheet1.zip › Supplementary Information/animals-Western blot/NFKB/NF-KB 2.tif]

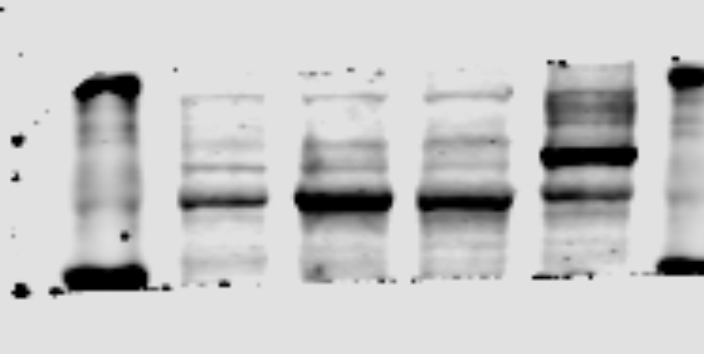

Supplement: Supplementary file 2 [file DataSheet1.zip › Supplementary Information/animals-Western blot/NFKB/NF-kB 3.tif]

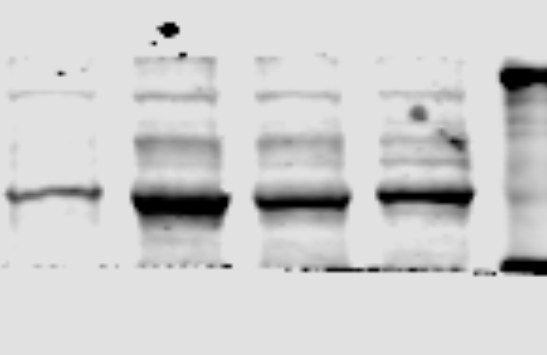

Supplement: Supplementary file 2 [file DataSheet1.zip › Supplementary Information/animals-Western blot/NFKB/NF-kB 4.tif]

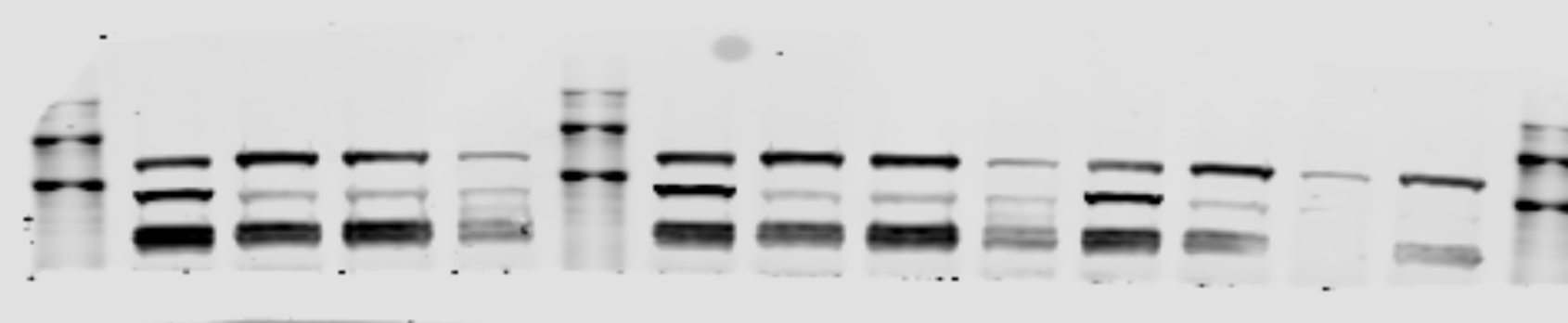

Supplement: Supplementary file 2 [file DataSheet1.zip › Supplementary Information/animals-Western blot/NLRP3/NLRP3-5.tif]

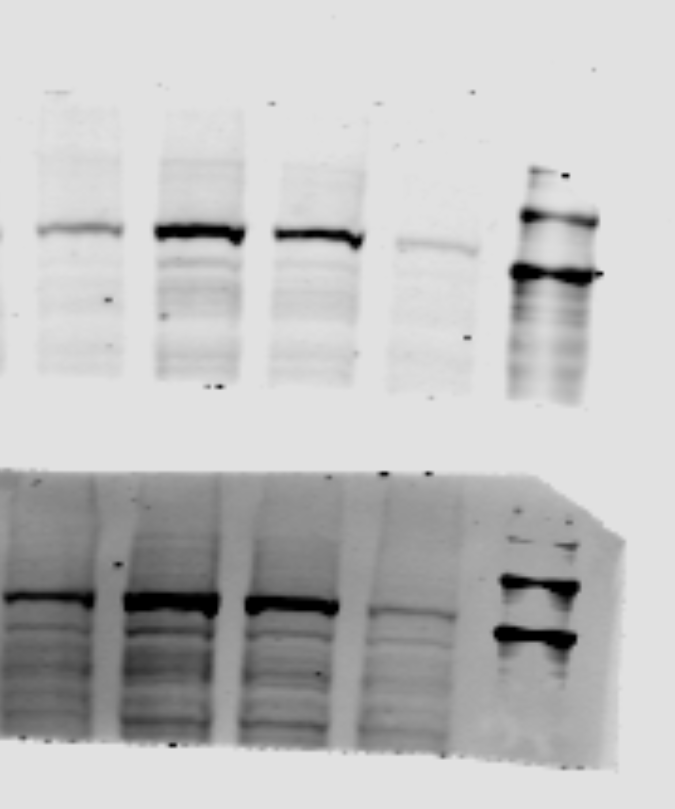

Supplement: Supplementary file 2 [file DataSheet1.zip › Supplementary Information/animals-Western blot/NLRP3/nlrp3 - 1.tif]

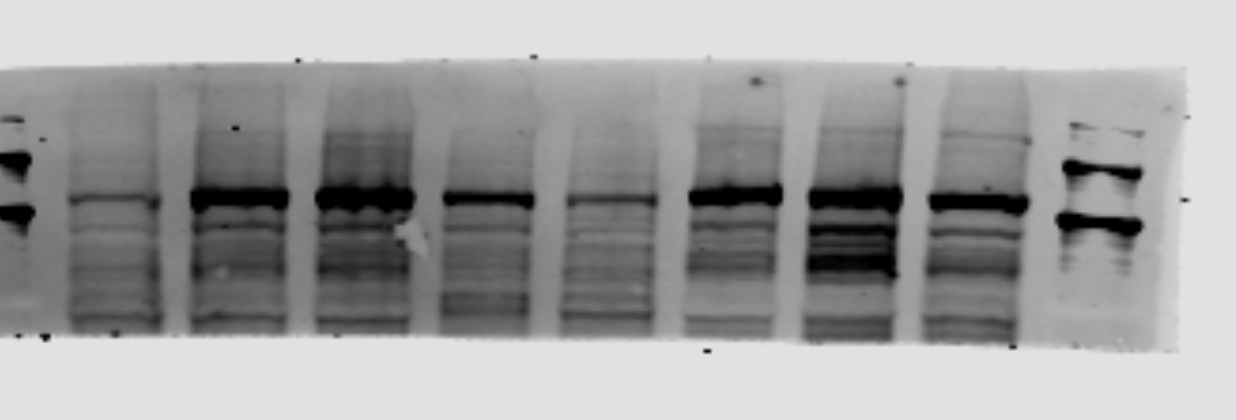

Supplement: Supplementary file 2 [file DataSheet1.zip › Supplementary Information/animals-Western blot/NLRP3/nlrp3 - 2.tif]

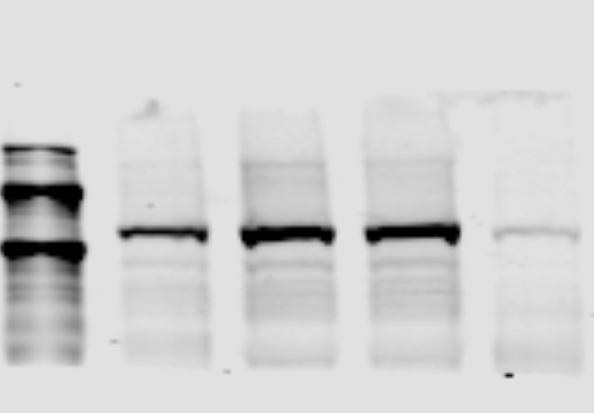

Supplement: Supplementary file 2 [file DataSheet1.zip › Supplementary Information/animals-Western blot/NLRP3/nlrp3 - 3.tif]

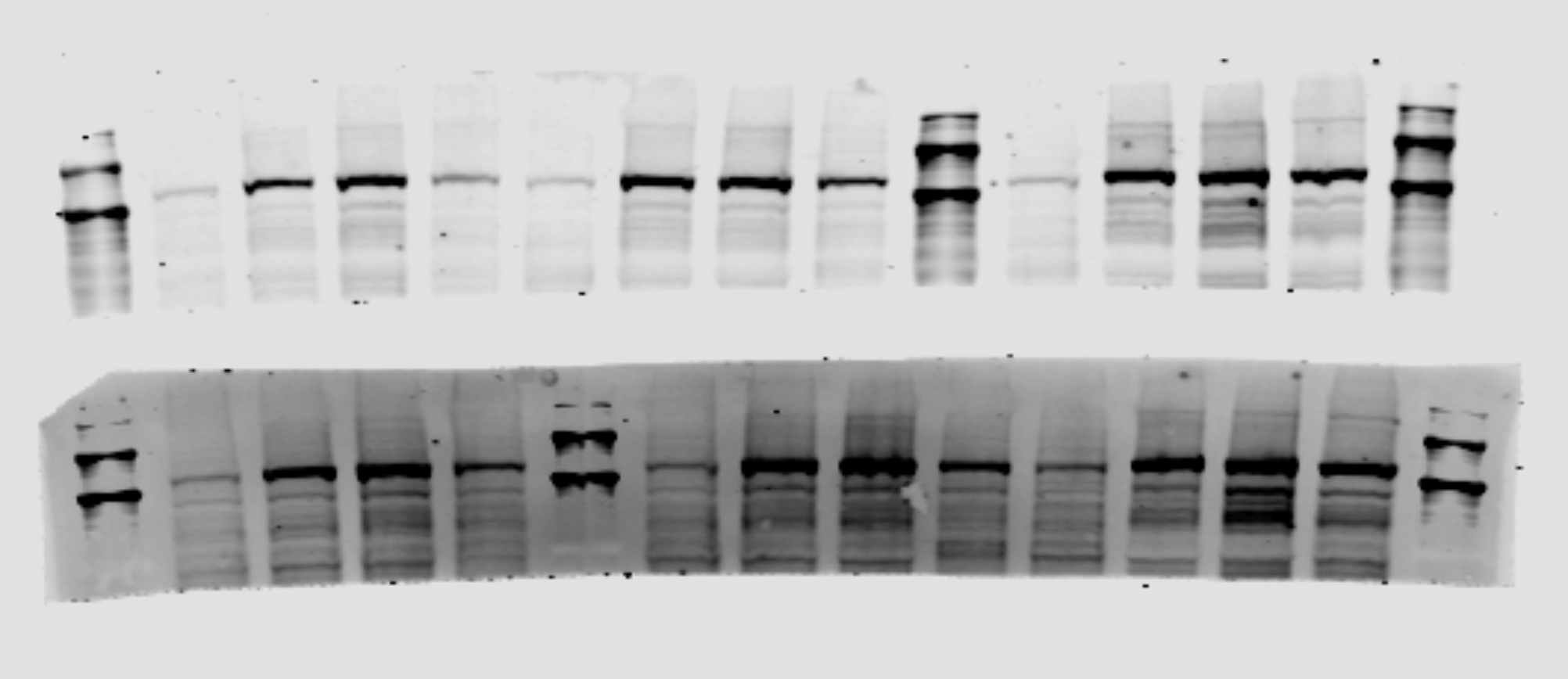

Supplement: Supplementary file 2 [file DataSheet1.zip › Supplementary Information/animals-Western blot/NLRP3/nlrp3-4.tif]

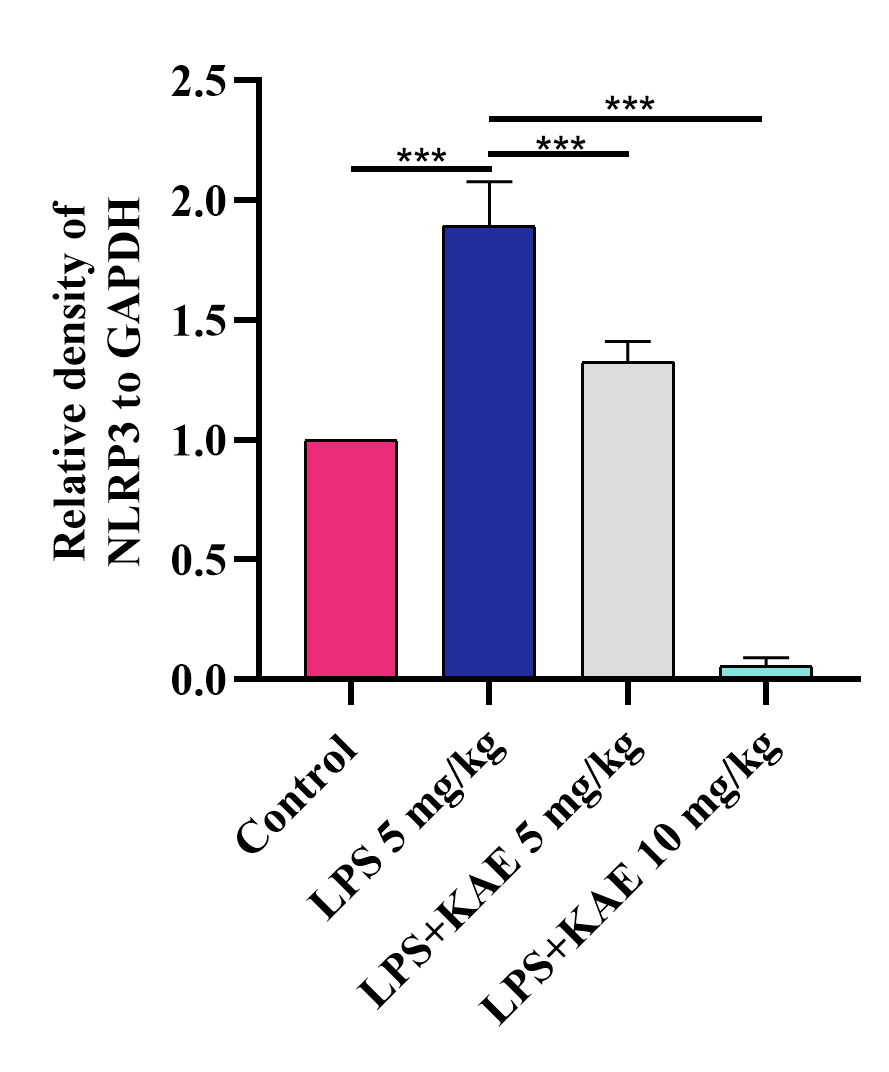

Supplement: Supplementary file 2 [file DataSheet1.zip › Supplementary Information/animals-Western blot/NLRP3.tif]

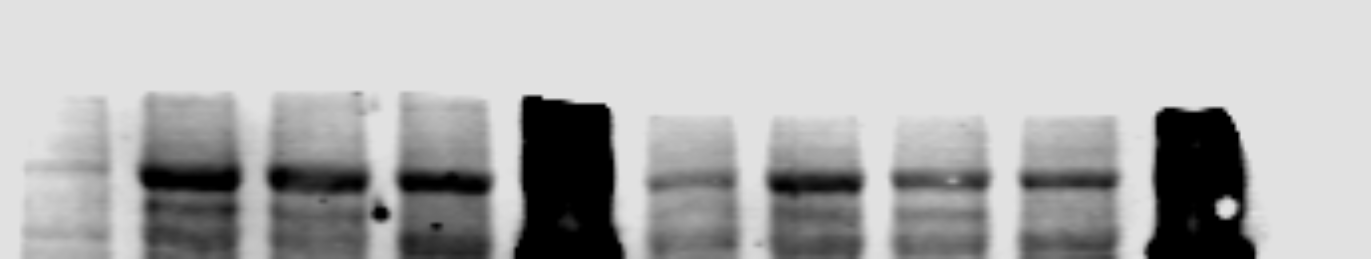

Supplement: Supplementary file 2 [file DataSheet1.zip › Supplementary Information/animals-Western blot/P-MLKL/P-MLKL.tif]

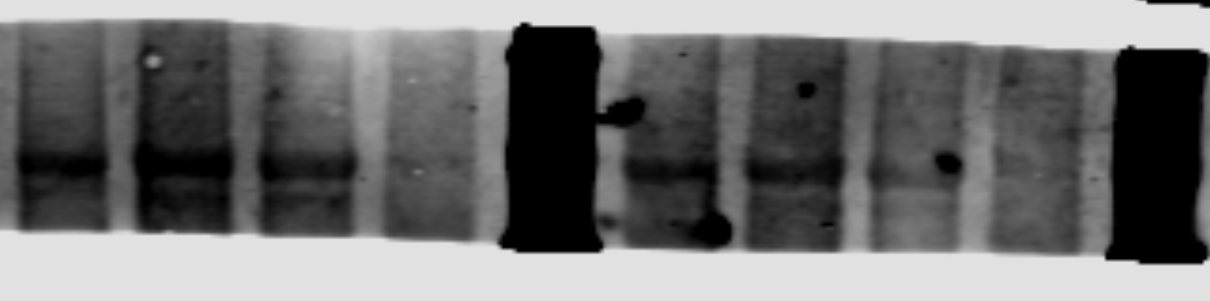

Supplement: Supplementary file 2 [file DataSheet1.zip › Supplementary Information/animals-Western blot/P-MLKL/p-mlkl2.tif]

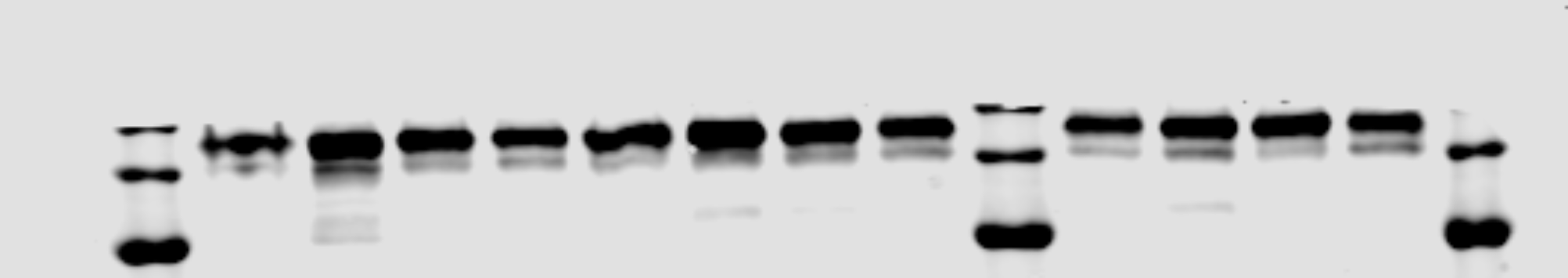

Supplement: Supplementary file 2 [file DataSheet1.zip › Supplementary Information/animals-Western blot/STING/STING 1.tif]

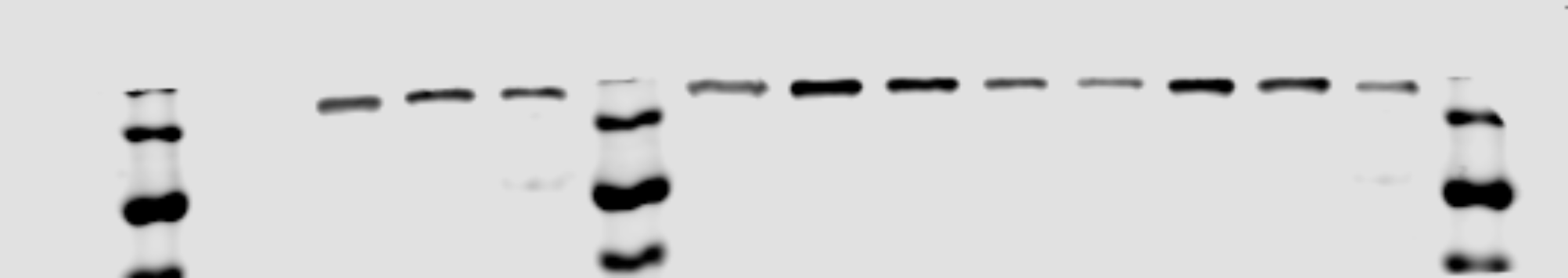

Supplement: Supplementary file 2 [file DataSheet1.zip › Supplementary Information/animals-Western blot/STING/STING 2.tif]

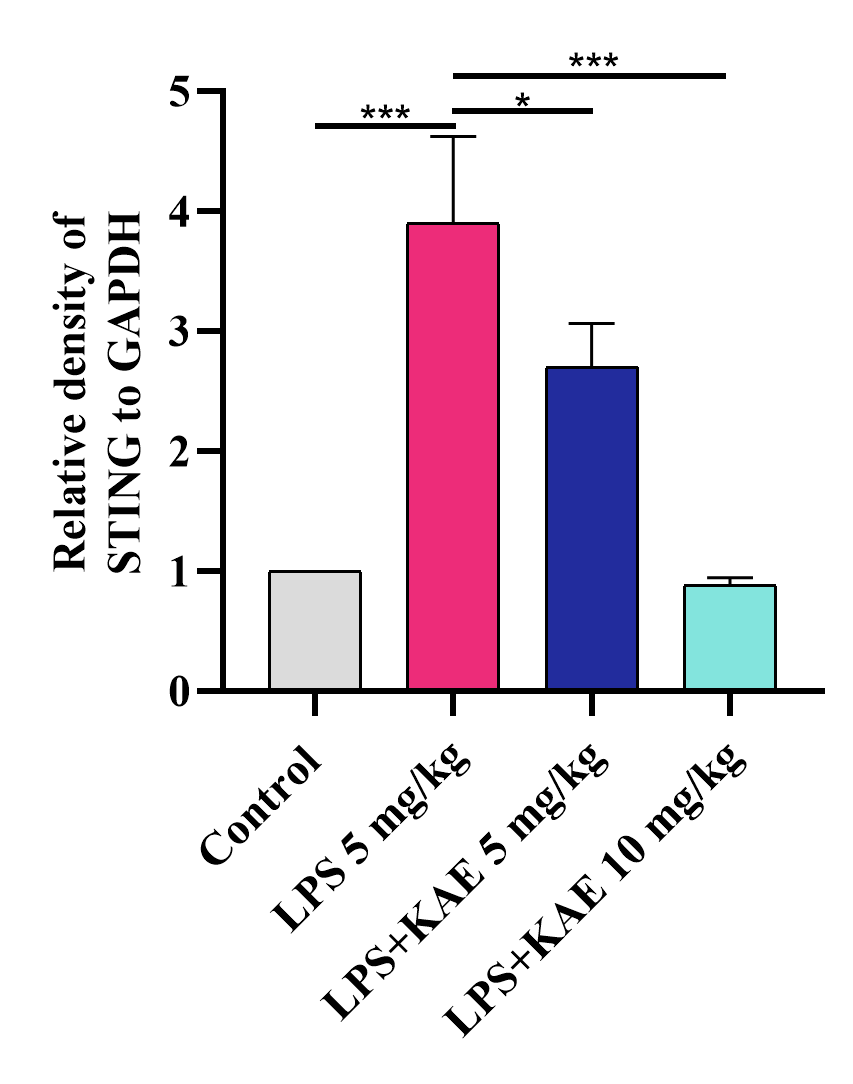

Supplement: Supplementary file 2 [file DataSheet1.zip › Supplementary Information/animals-Western blot/STING.tif]

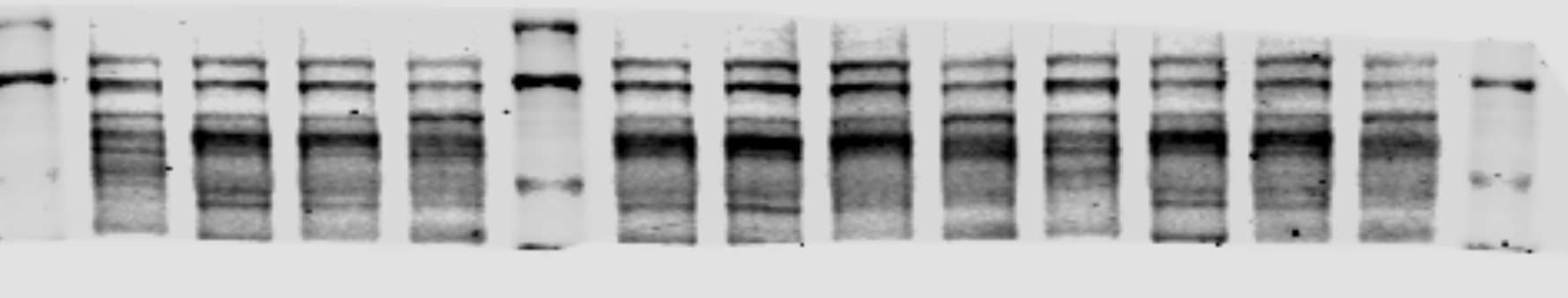

Supplement: Supplementary file 2 [file DataSheet1.zip › Supplementary Information/animals-Western blot/ZBP1/zbp1-1.tif]

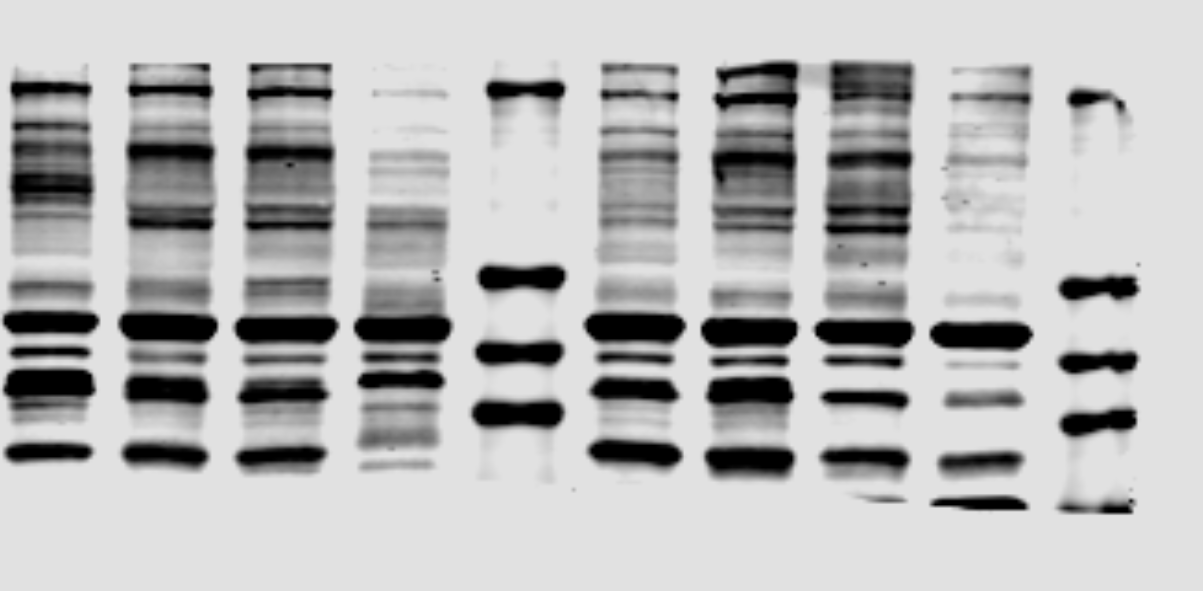

Supplement: Supplementary file 2 [file DataSheet1.zip › Supplementary Information/animals-Western blot/ZBP1/zbp1-2.tif]

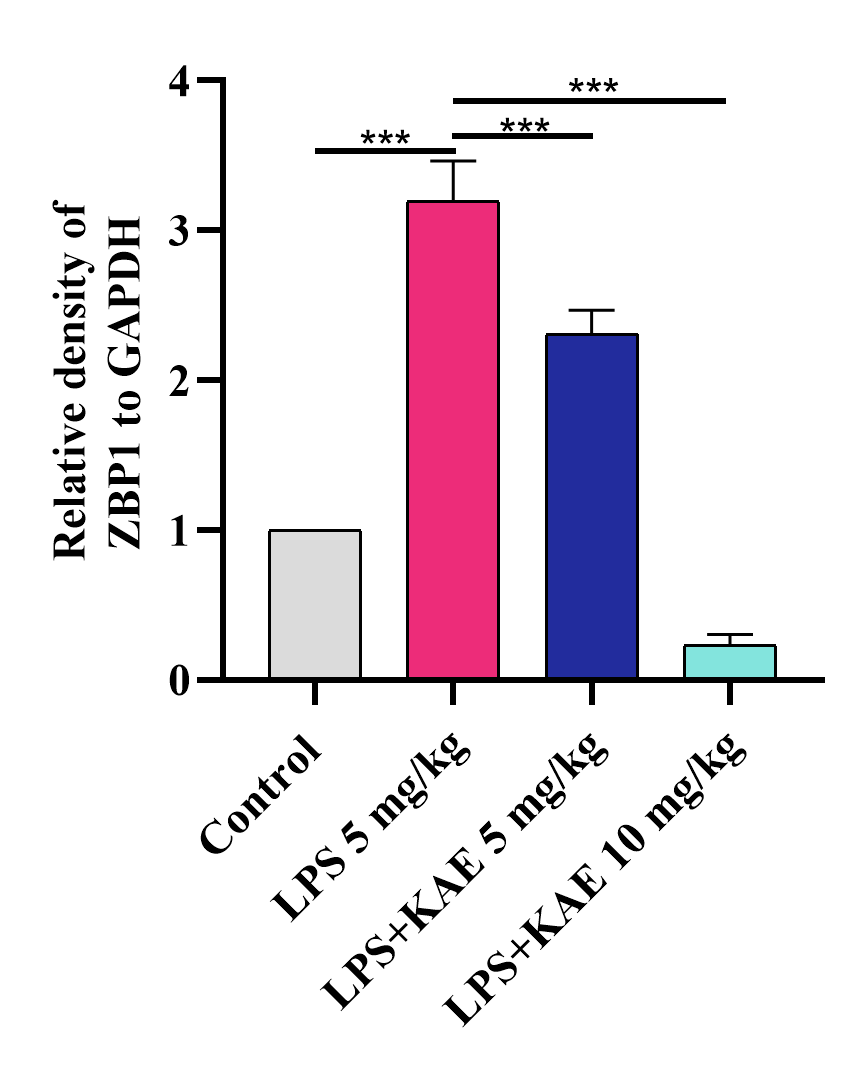

Supplement: Supplementary file 2 [file DataSheet1.zip › Supplementary Information/animals-Western blot/ZBP1.tif]

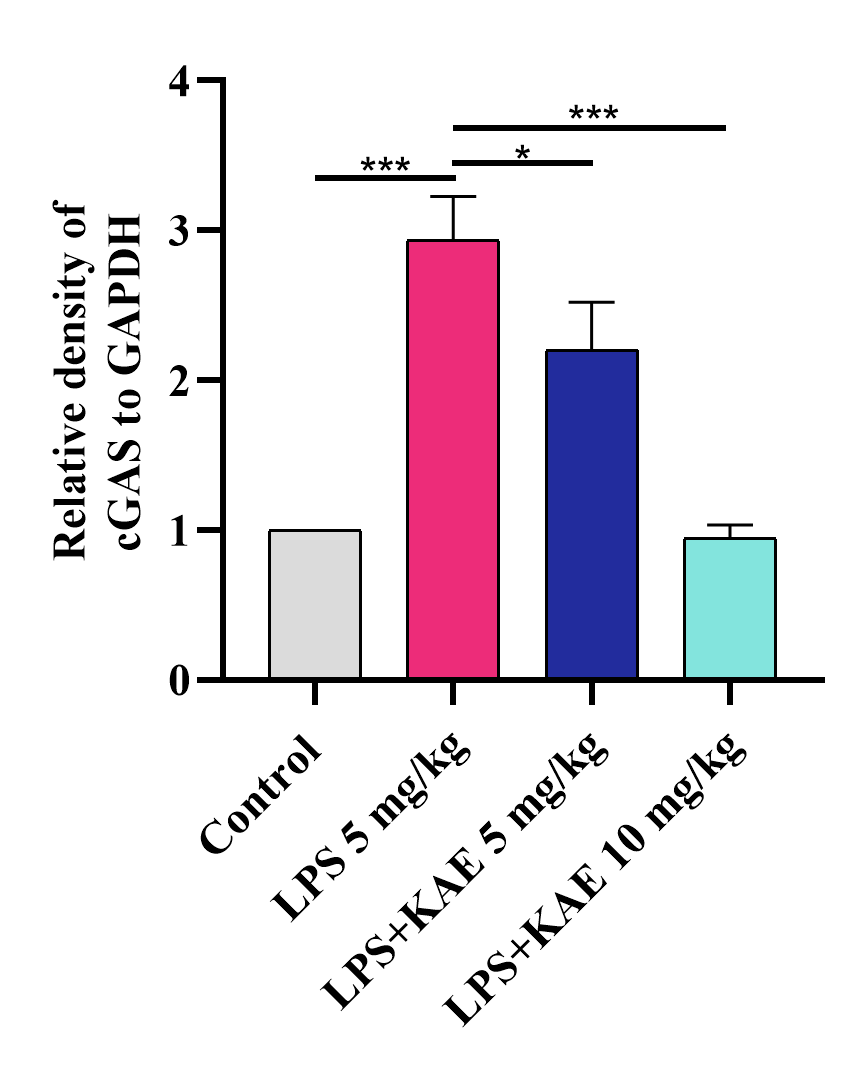

Supplement: Supplementary file 2 [file DataSheet1.zip › Supplementary Information/animals-Western blot/cGAS.tif]

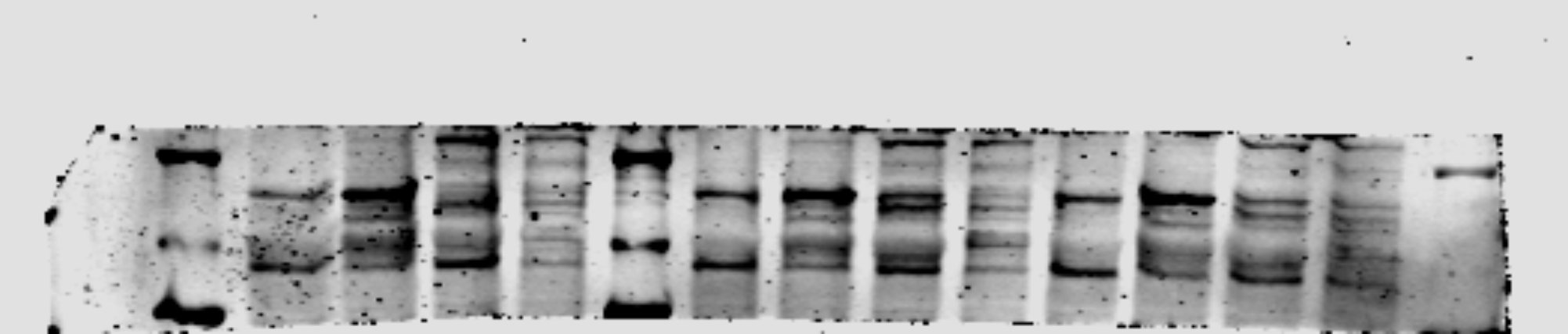

Supplement: Supplementary file 2 [file DataSheet1.zip › Supplementary Information/animals-Western blot/p-TBK1/P-TBK1 1.tif]

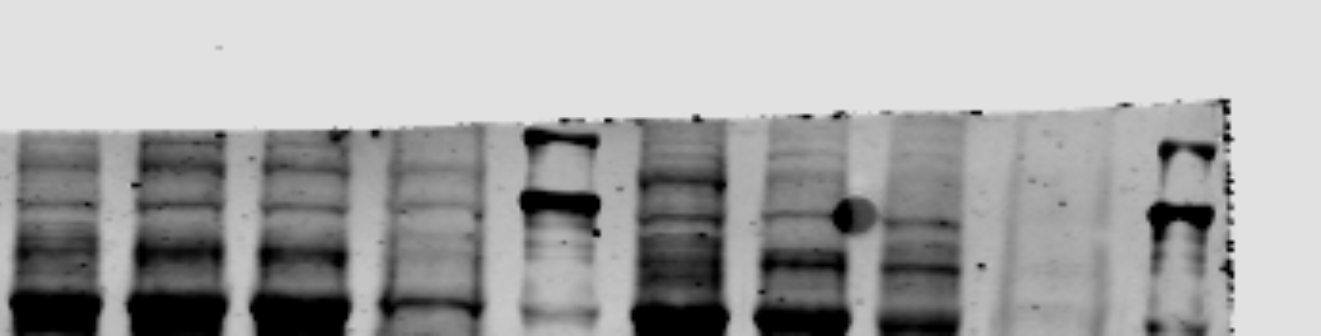

Supplement: Supplementary file 2 [file DataSheet1.zip › Supplementary Information/animals-Western blot/p-TBK1/P-tbk1 2..tif]

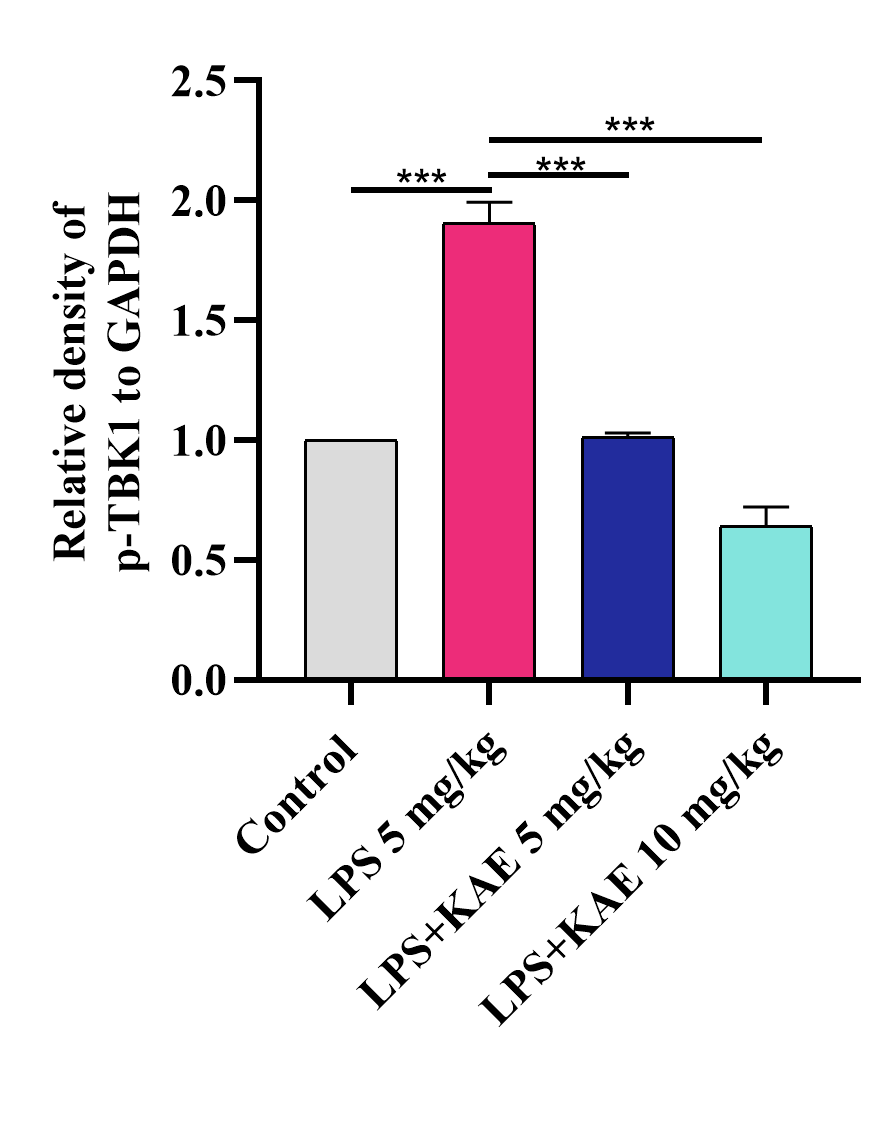

Supplement: Supplementary file 2 [file DataSheet1.zip › Supplementary Information/animals-Western blot/p-TBK1.tif]

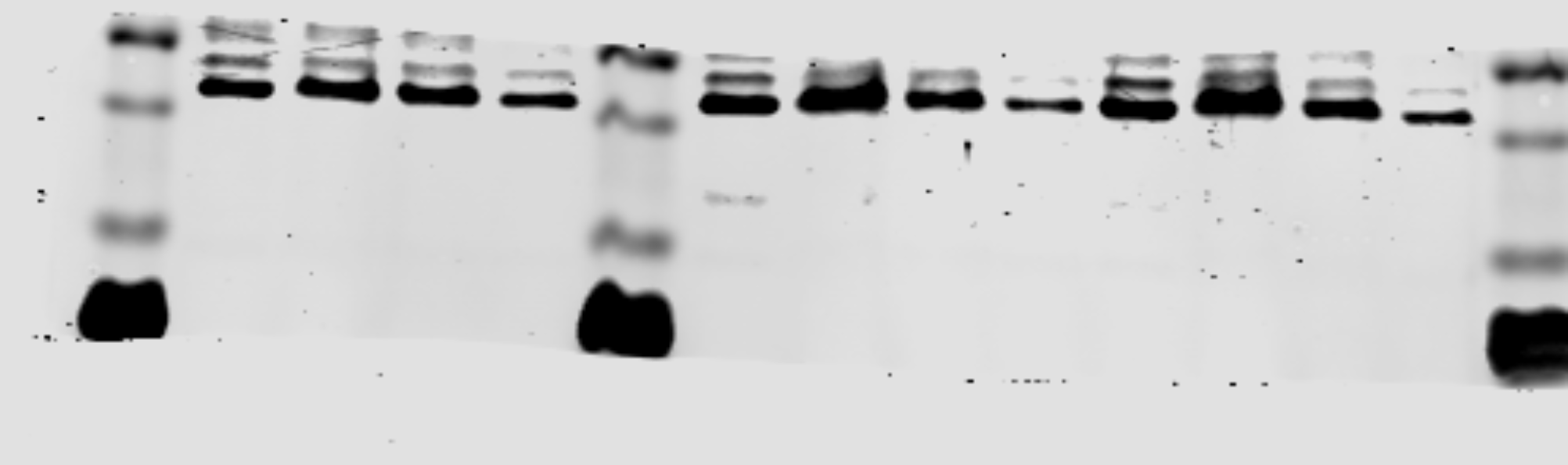

Supplement: Supplementary file 2 [file DataSheet1.zip › Supplementary Information/cell-Western blot/ASC/ASC.tif]

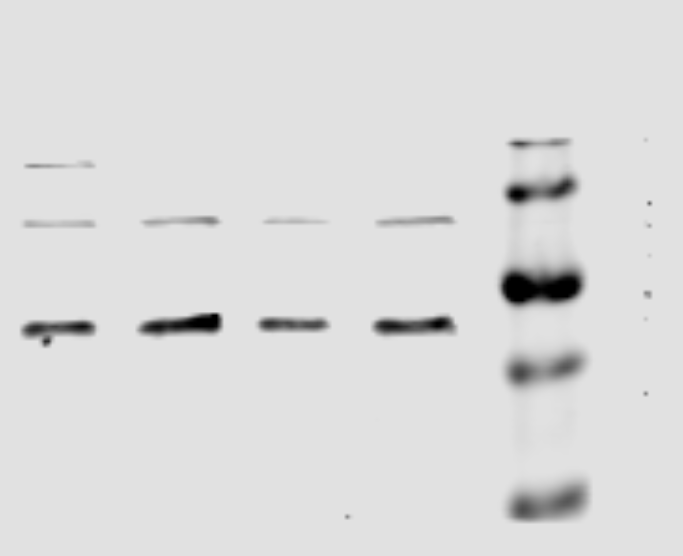

Supplement: Supplementary file 2 [file DataSheet1.zip › Supplementary Information/cell-Western blot/ASC/asc-1.tif]

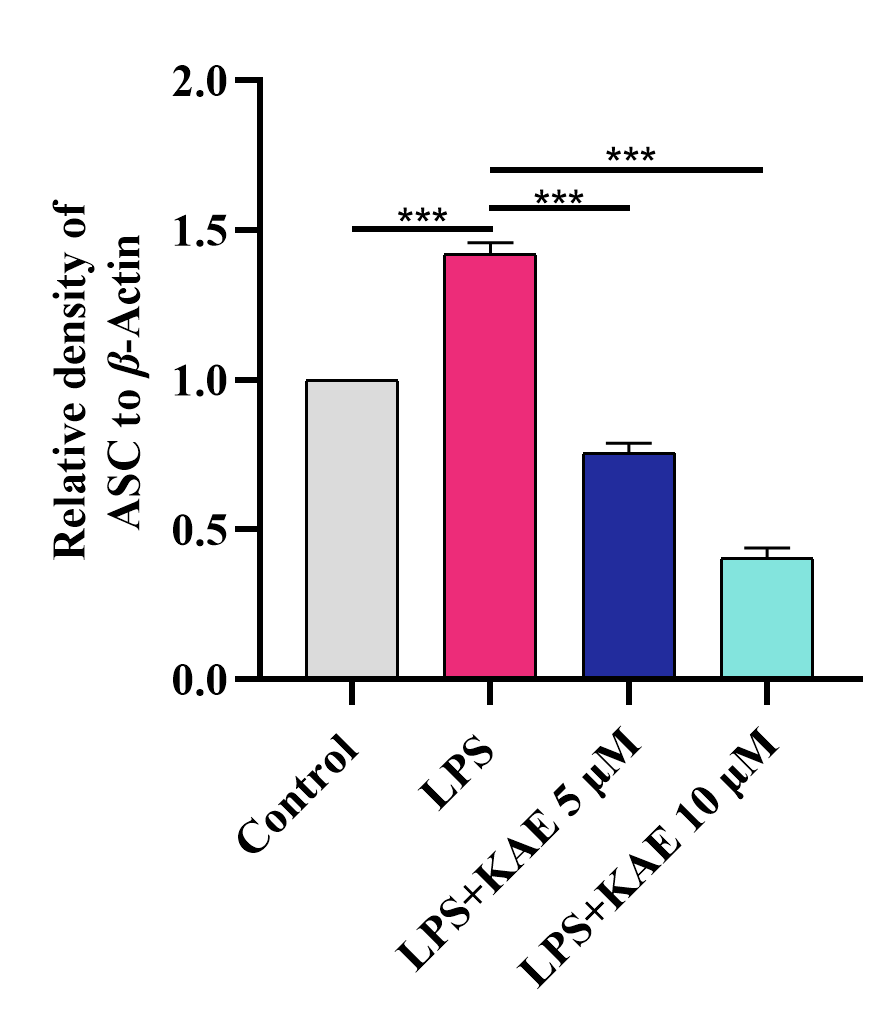

Supplement: Supplementary file 2 [file DataSheet1.zip › Supplementary Information/cell-Western blot/ASC.tif]

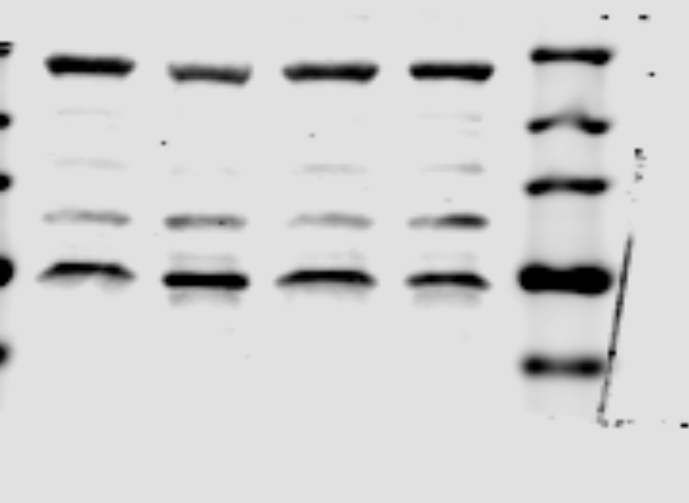

Supplement: Supplementary file 2 [file DataSheet1.zip › Supplementary Information/cell-Western blot/CASP1/casp1 11 - 副本.tif]

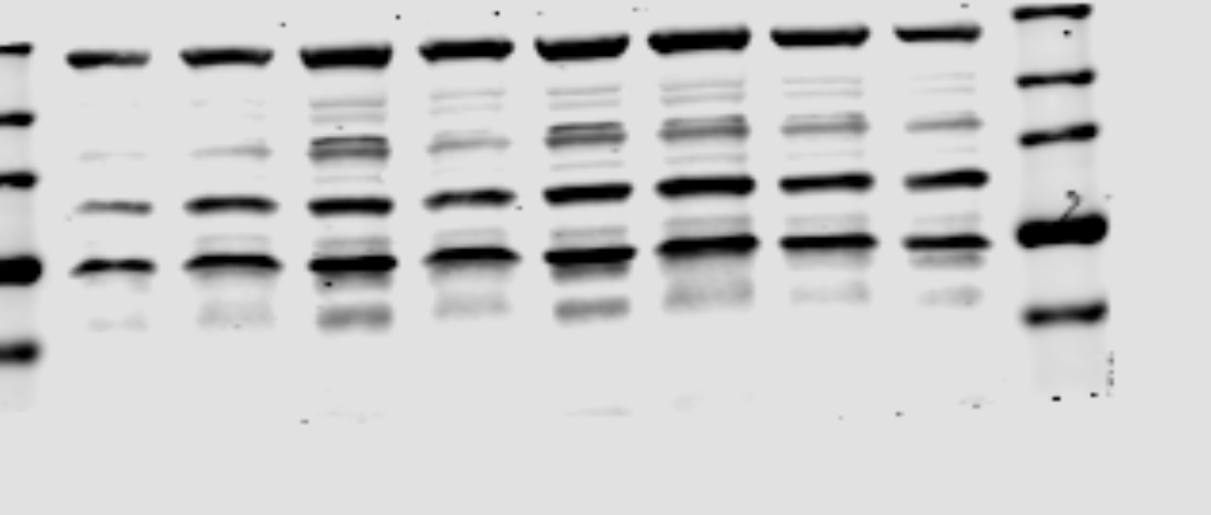

Supplement: Supplementary file 2 [file DataSheet1.zip › Supplementary Information/cell-Western blot/CASP1/casp1 11.tif]

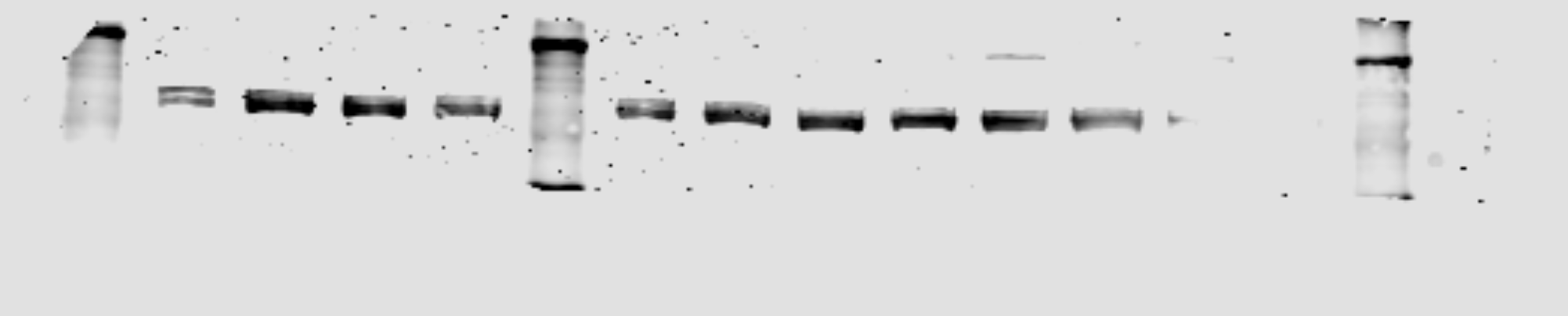

Supplement: Supplementary file 2 [file DataSheet1.zip › Supplementary Information/cell-Western blot/CGAS/cgas1.tif]

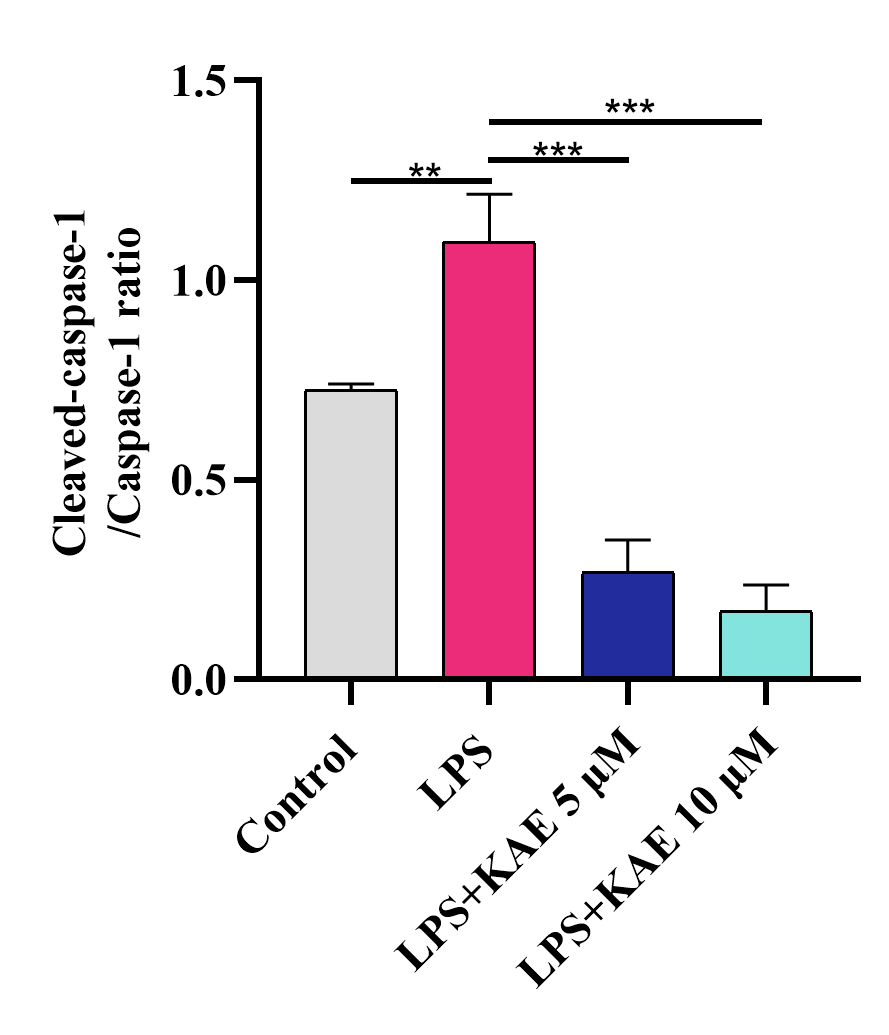

Supplement: Supplementary file 2 [file DataSheet1.zip › Supplementary Information/cell-Western blot/Caspase-1.tif]

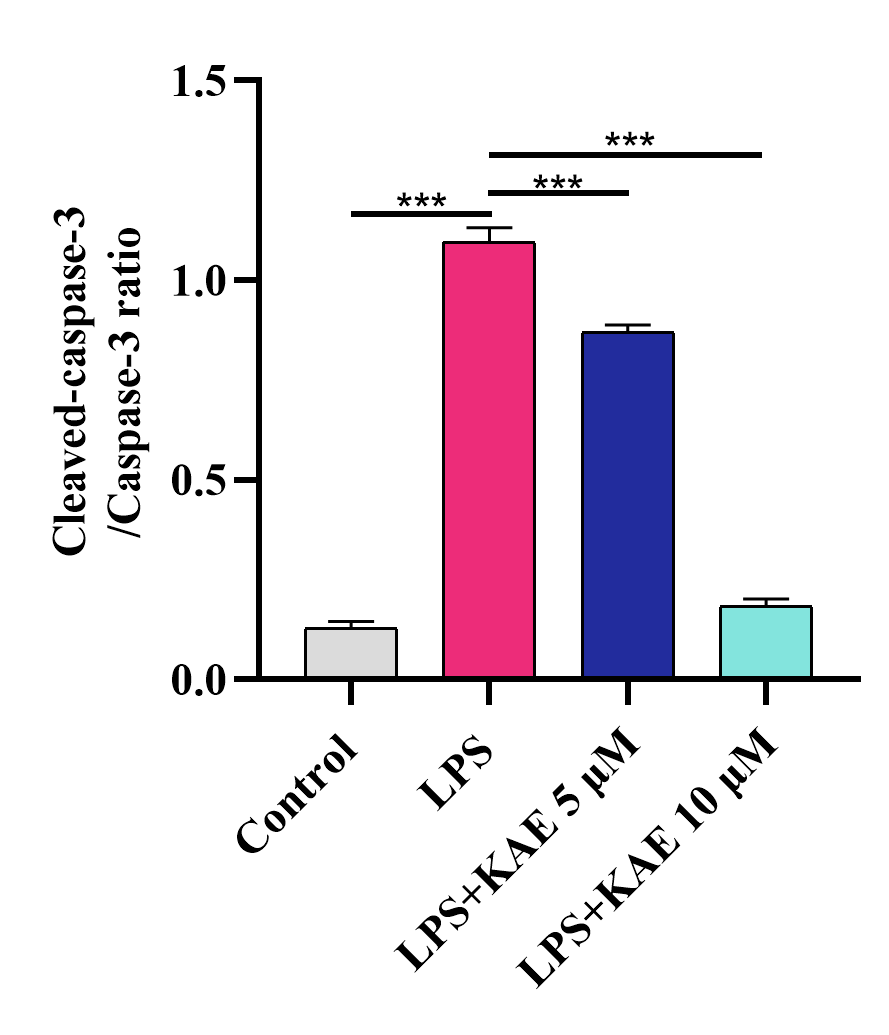

Supplement: Supplementary file 2 [file DataSheet1.zip › Supplementary Information/cell-Western blot/Caspase-3.tif]

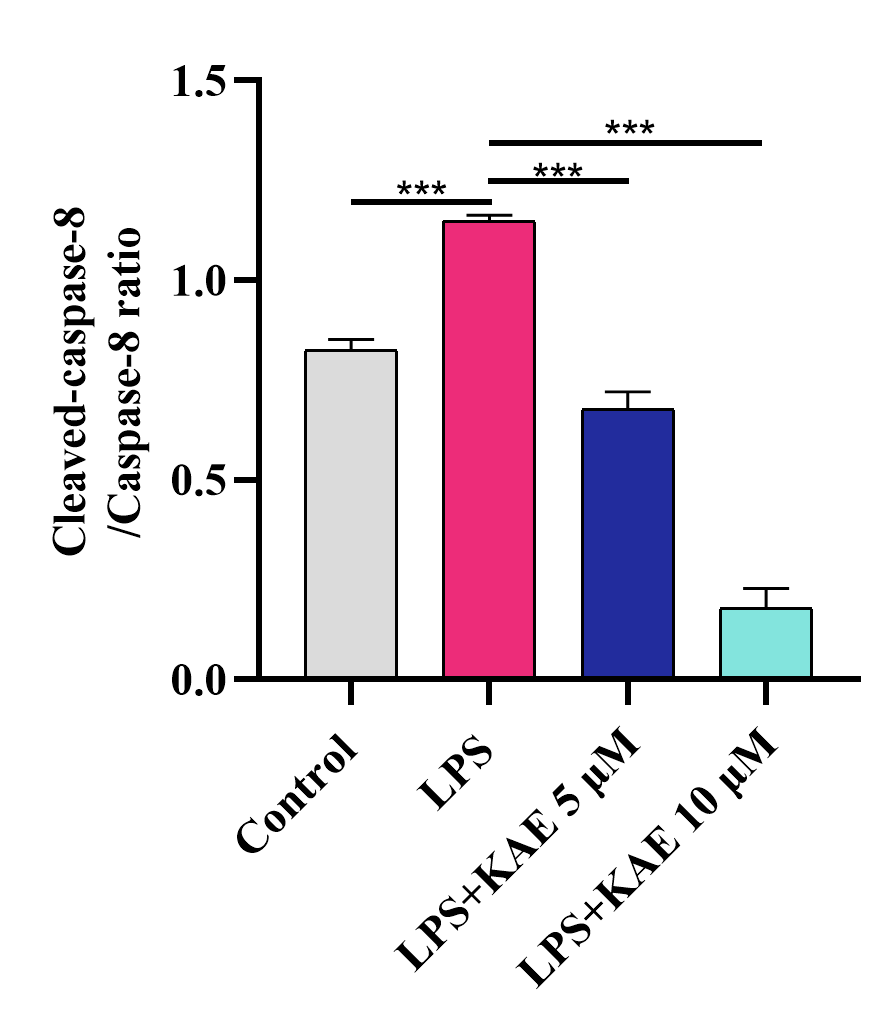

Supplement: Supplementary file 2 [file DataSheet1.zip › Supplementary Information/cell-Western blot/Caspase-8.tif]

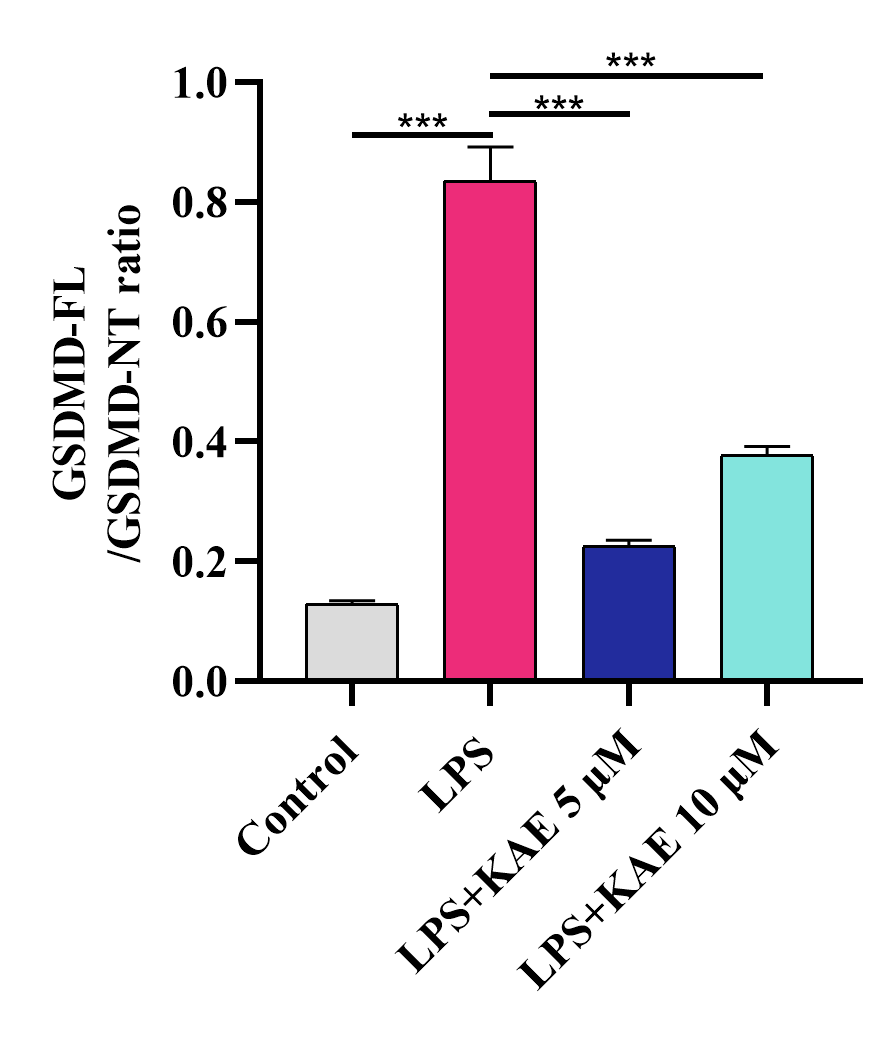

Supplement: Supplementary file 2 [file DataSheet1.zip › Supplementary Information/cell-Western blot/GSDMD.tif]

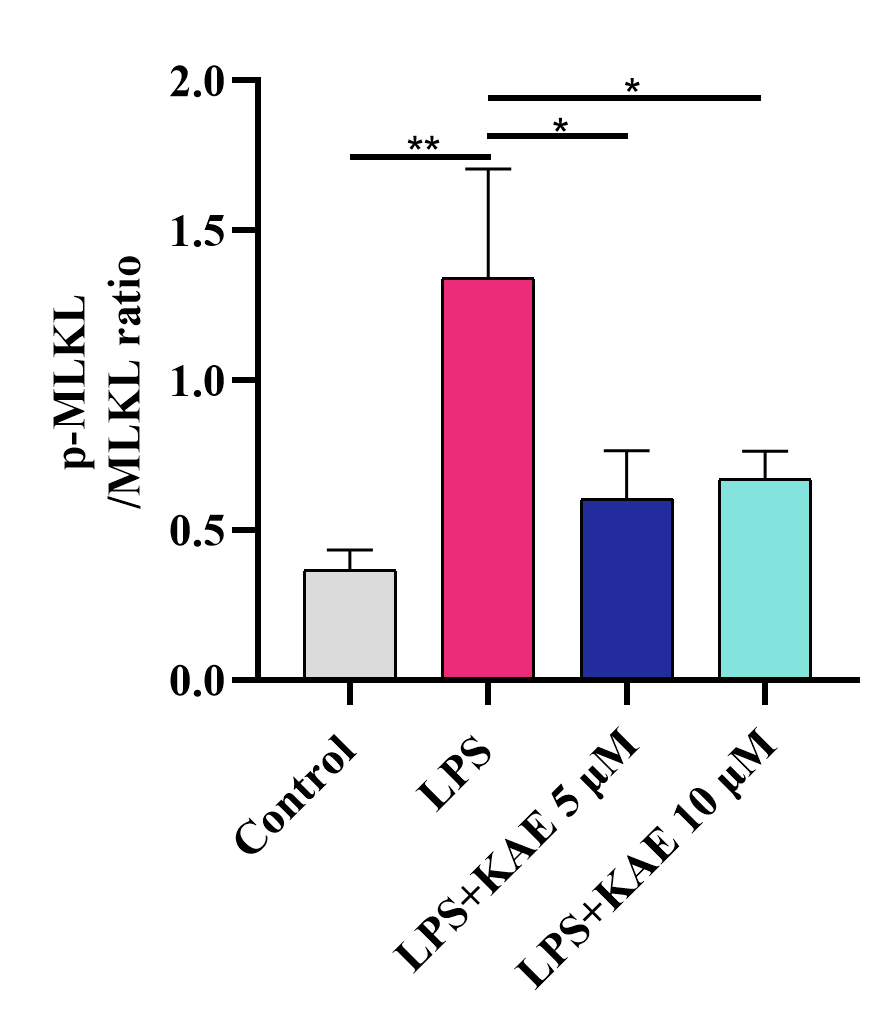

Supplement: Supplementary file 2 [file DataSheet1.zip › Supplementary Information/cell-Western blot/MLKL.tif]

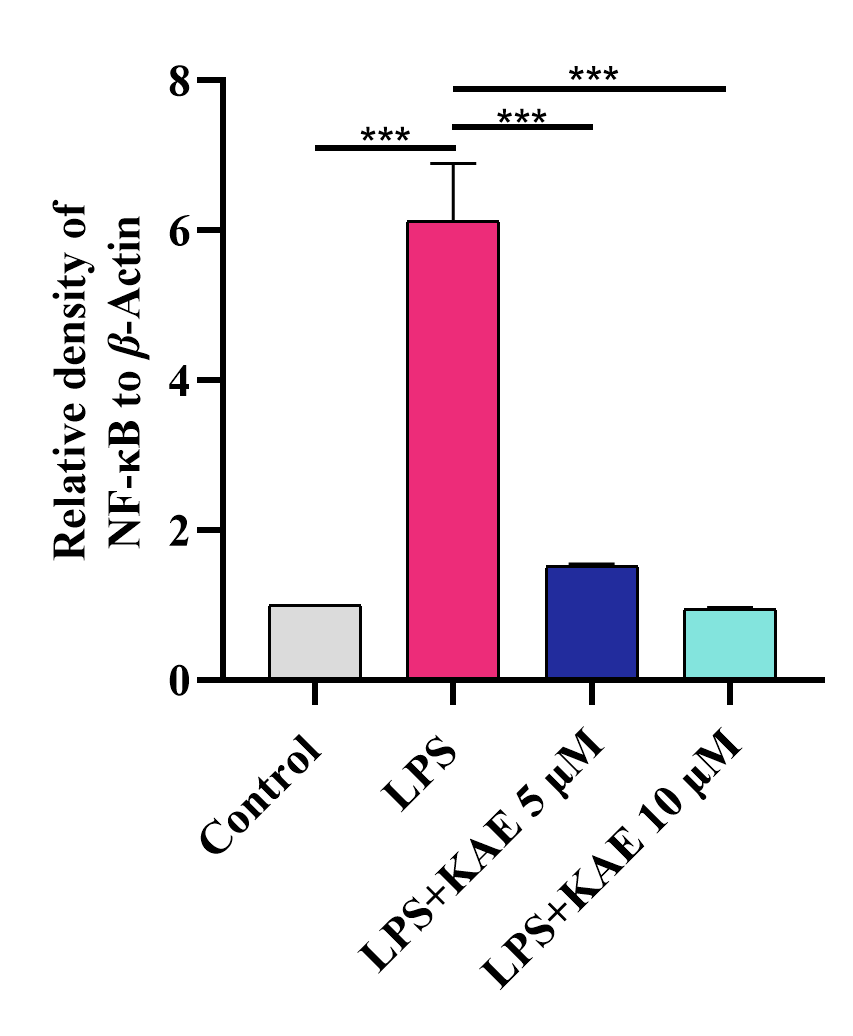

Supplement: Supplementary file 2 [file DataSheet1.zip › Supplementary Information/cell-Western blot/NF-KB.tif]

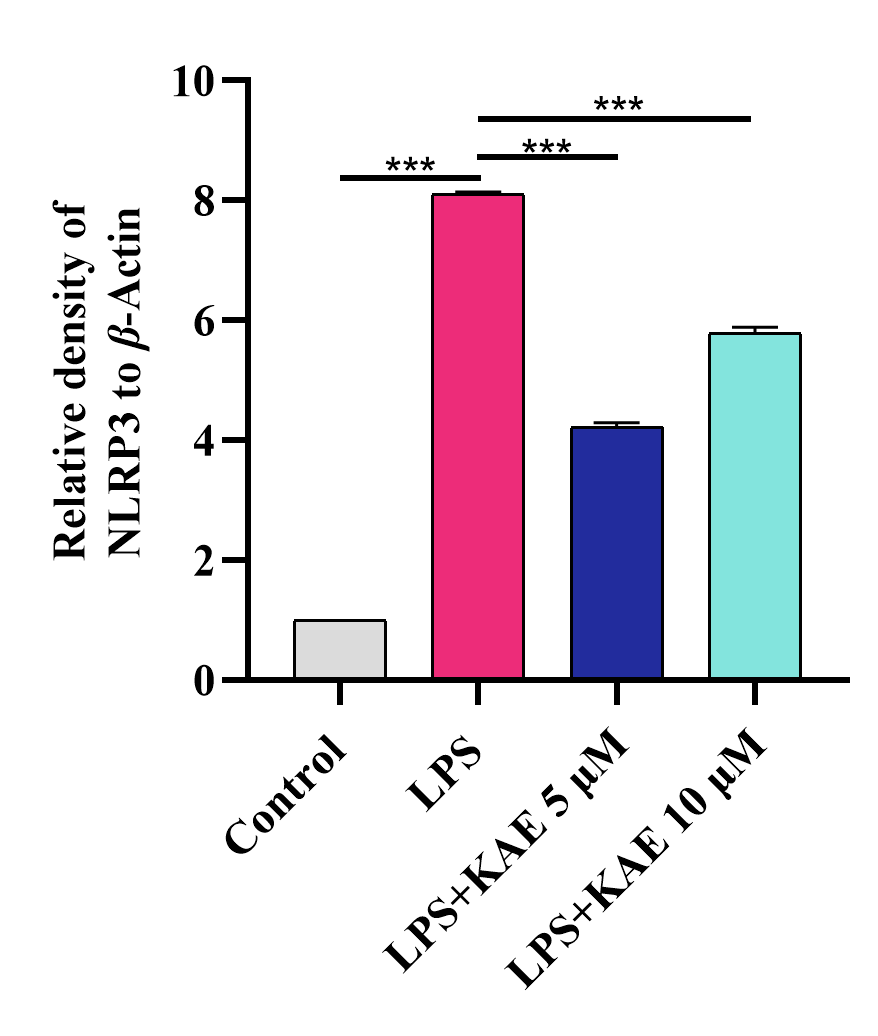

Supplement: Supplementary file 2 [file DataSheet1.zip › Supplementary Information/cell-Western blot/NLRP3.tif]

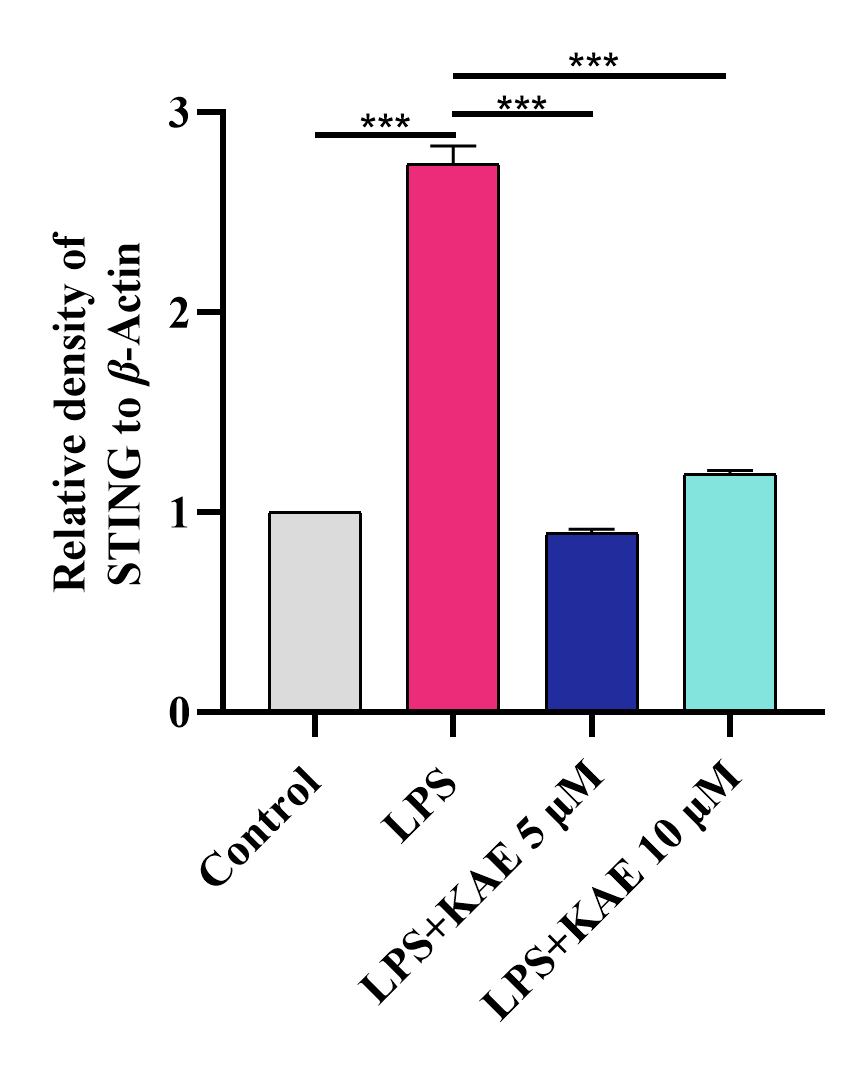

Supplement: Supplementary file 2 [file DataSheet1.zip › Supplementary Information/cell-Western blot/STING.tif]

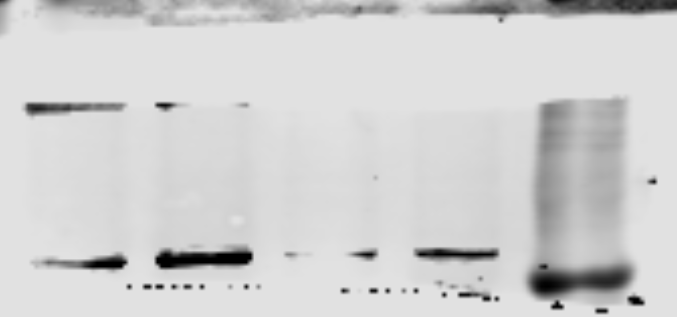

Supplement: Supplementary file 2 [file DataSheet1.zip › Supplementary Information/cell-Western blot/ZBP1/ZBP1 2.tif]

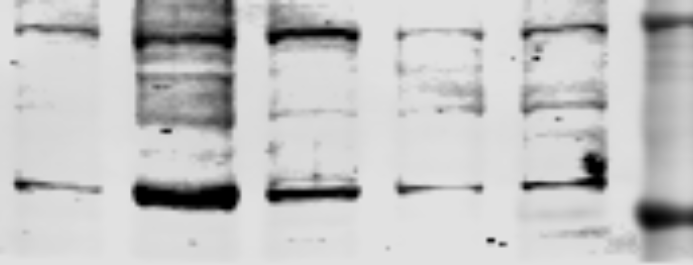

Supplement: Supplementary file 2 [file DataSheet1.zip › Supplementary Information/cell-Western blot/ZBP1/ZBP1 3.tif]

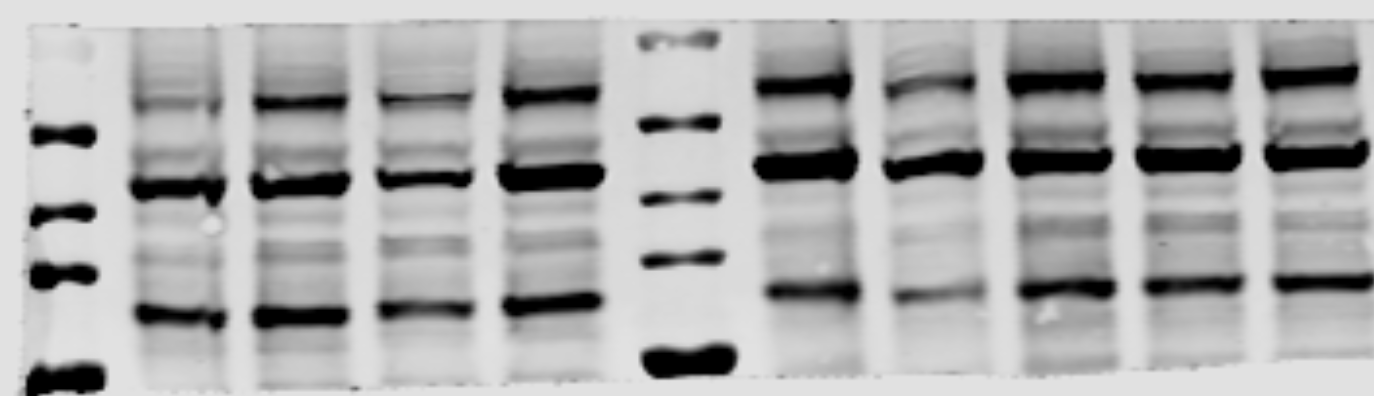

Supplement: Supplementary file 2 [file DataSheet1.zip › Supplementary Information/cell-Western blot/ZBP1/zbp1.tif]

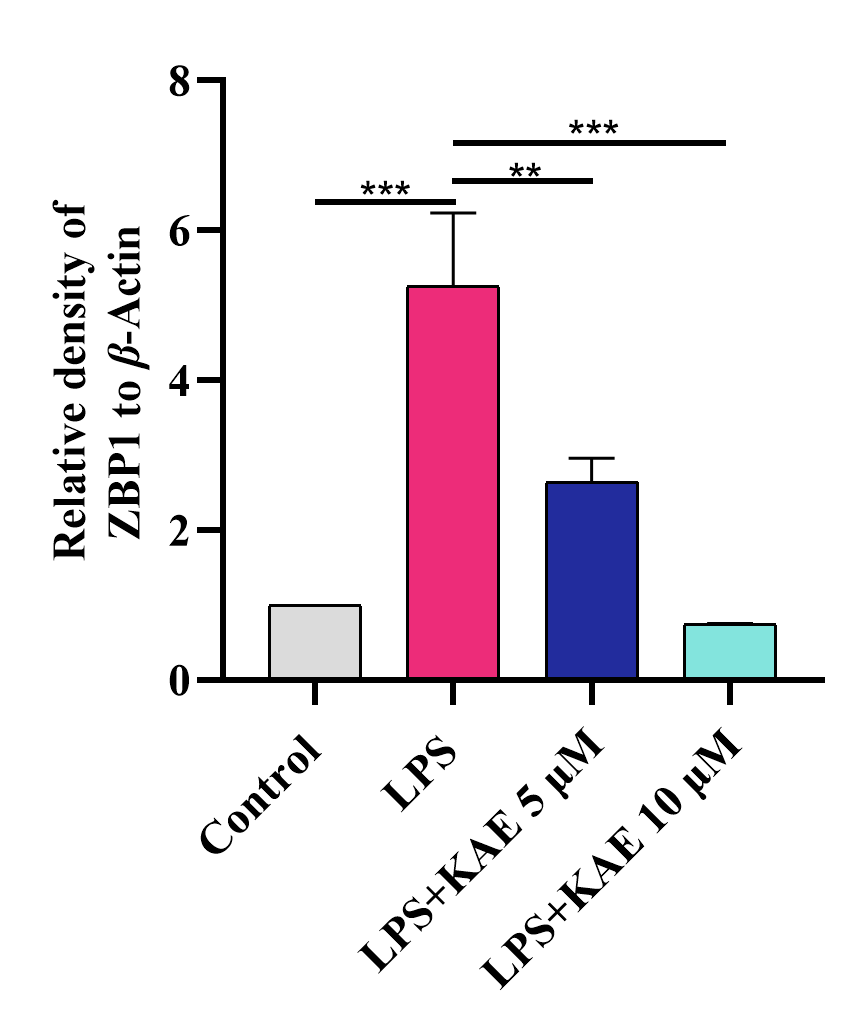

Supplement: Supplementary file 2 [file DataSheet1.zip › Supplementary Information/cell-Western blot/ZBP1.tif]

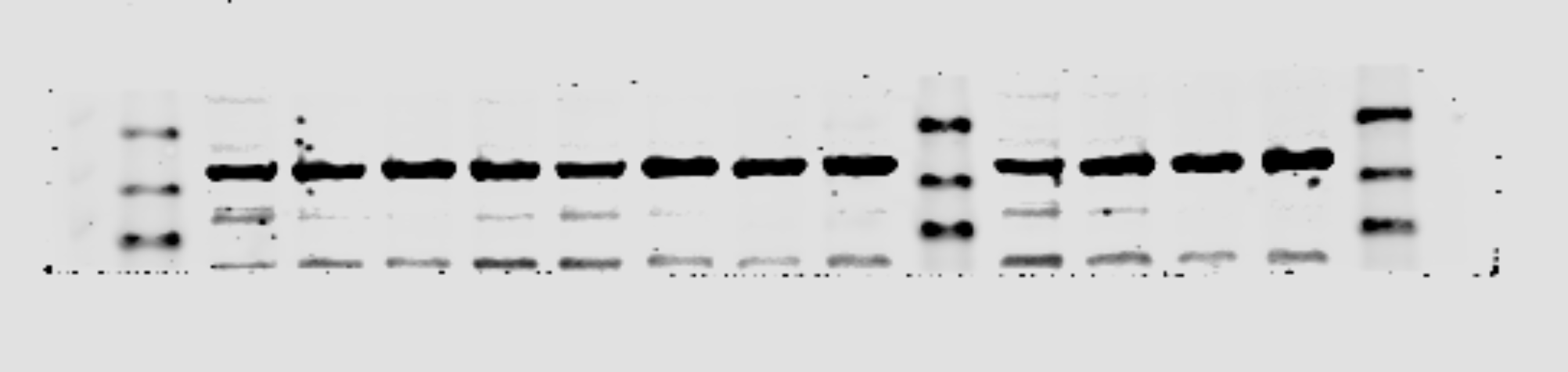

Supplement: Supplementary file 2 [file DataSheet1.zip › Supplementary Information/cell-Western blot/b-actin/actin-1.tif]

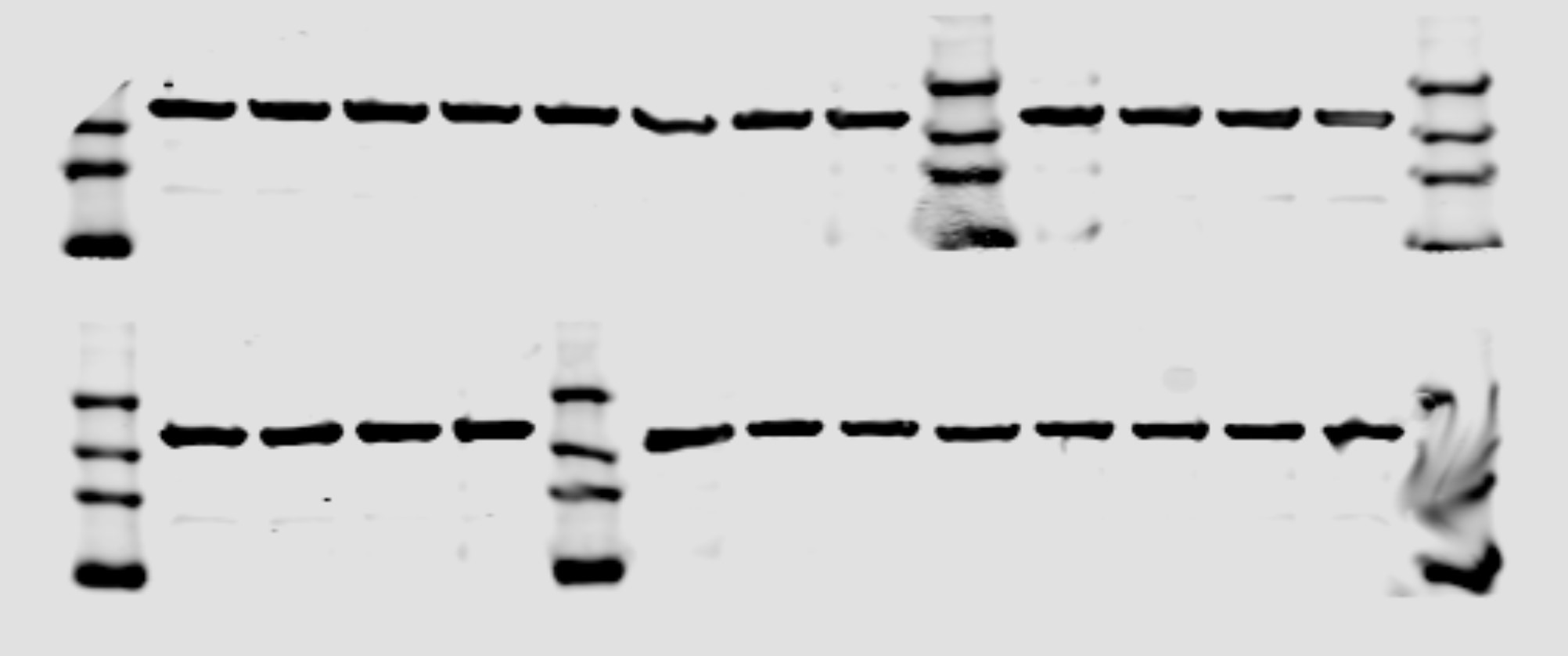

Supplement: Supplementary file 2 [file DataSheet1.zip › Supplementary Information/cell-Western blot/b-actin/actin.png]

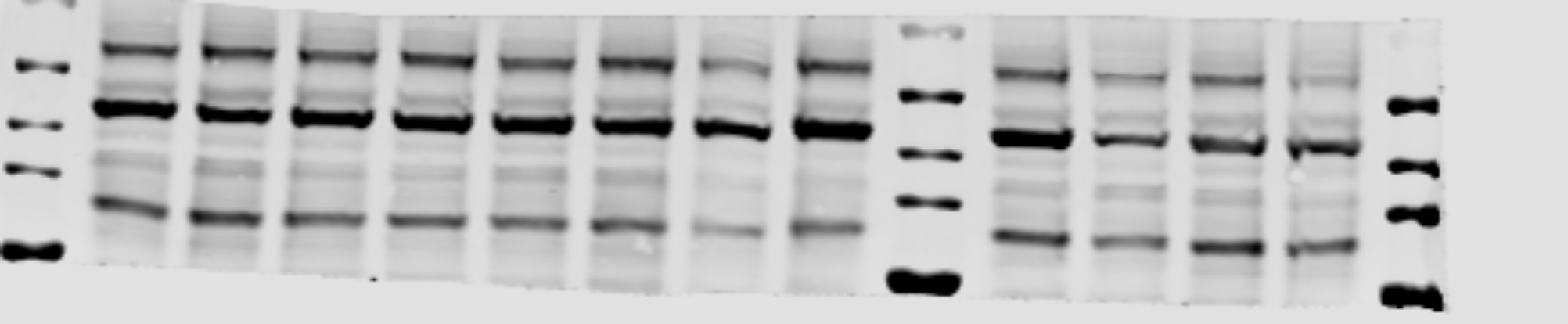

Supplement: Supplementary file 2 [file DataSheet1.zip › Supplementary Information/cell-Western blot/b-actin/b-actin 123.tif]

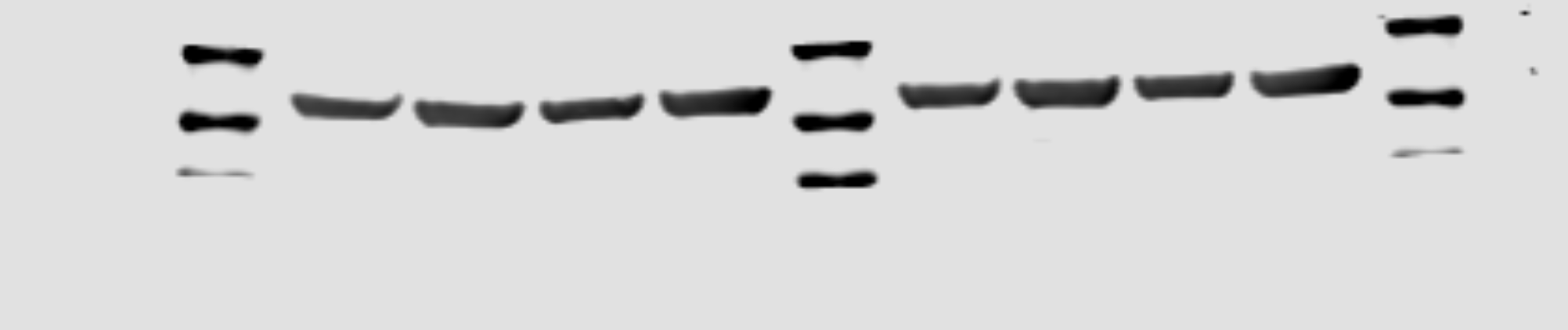

Supplement: Supplementary file 2 [file DataSheet1.zip › Supplementary Information/cell-Western blot/b-actin/b-actin.tif]

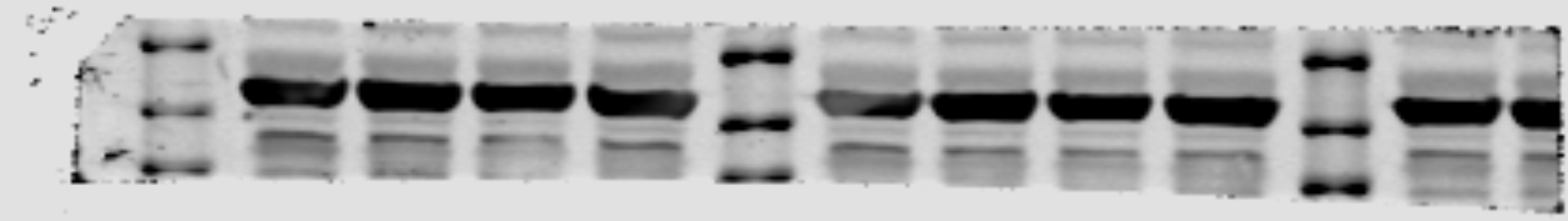

Supplement: Supplementary file 2 [file DataSheet1.zip › Supplementary Information/cell-Western blot/b-actin/b-actin1.tif]

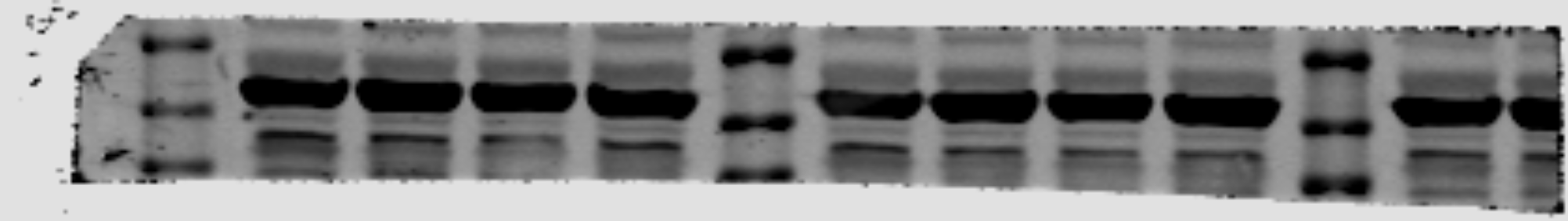

Supplement: Supplementary file 2 [file DataSheet1.zip › Supplementary Information/cell-Western blot/b-actin/b-actin2.tif]

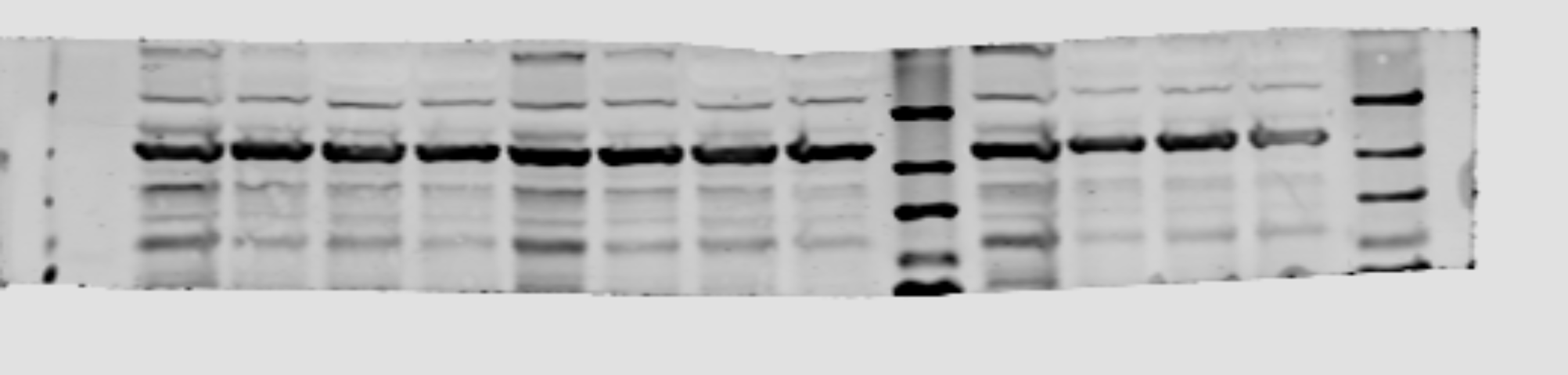

Supplement: Supplementary file 2 [file DataSheet1.zip › Supplementary Information/cell-Western blot/b-actin/b-actin4.tif]

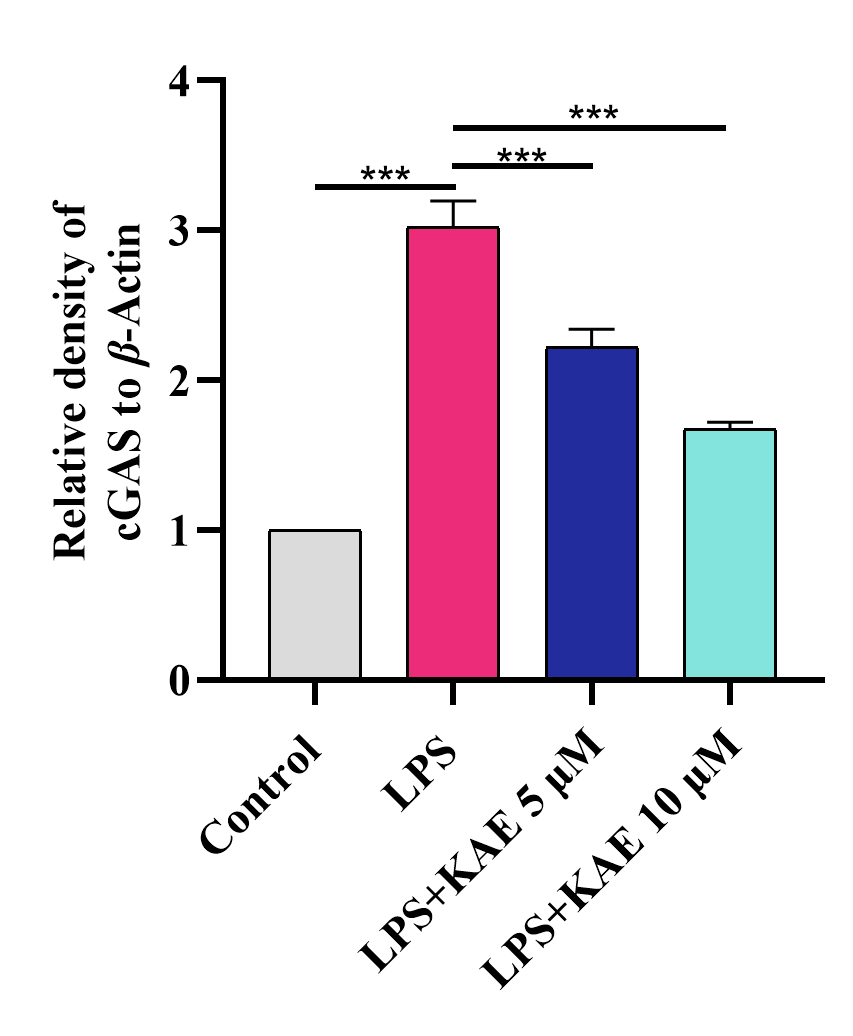

Supplement: Supplementary file 2 [file DataSheet1.zip › Supplementary Information/cell-Western blot/cGAS.tif]

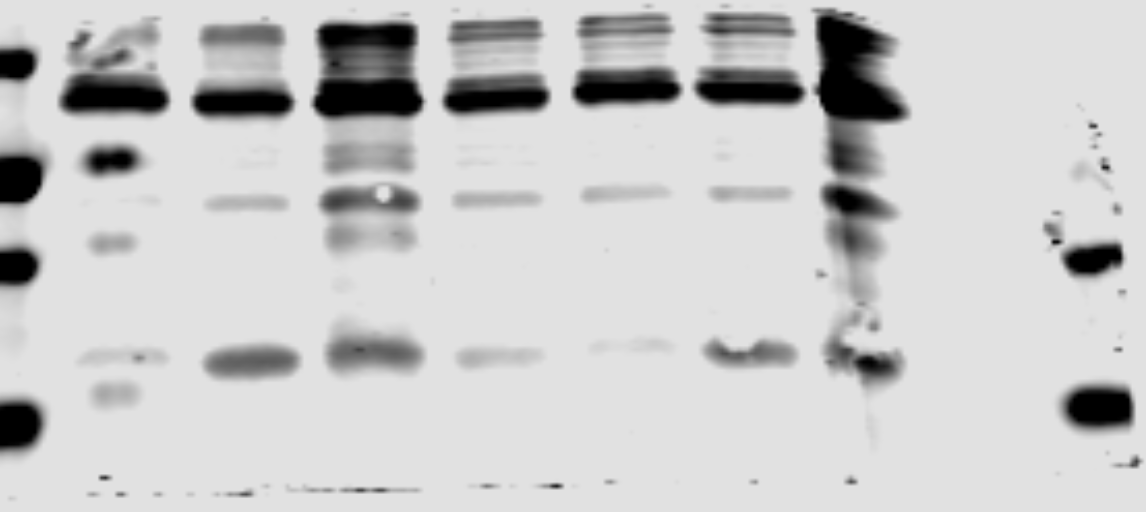

Supplement: Supplementary file 2 [file DataSheet1.zip › Supplementary Information/cell-Western blot/caspase3/caspase3 23 - 副本.tif]

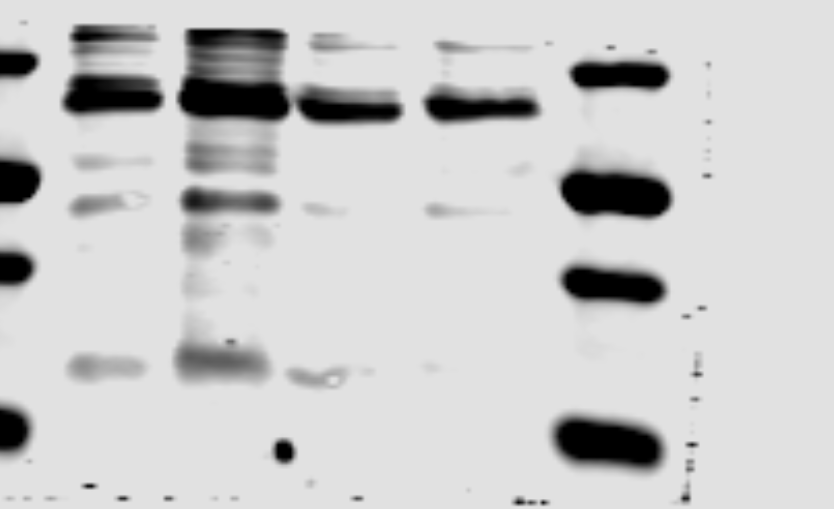

Supplement: Supplementary file 2 [file DataSheet1.zip › Supplementary Information/cell-Western blot/caspase3/caspase3 23.tif]

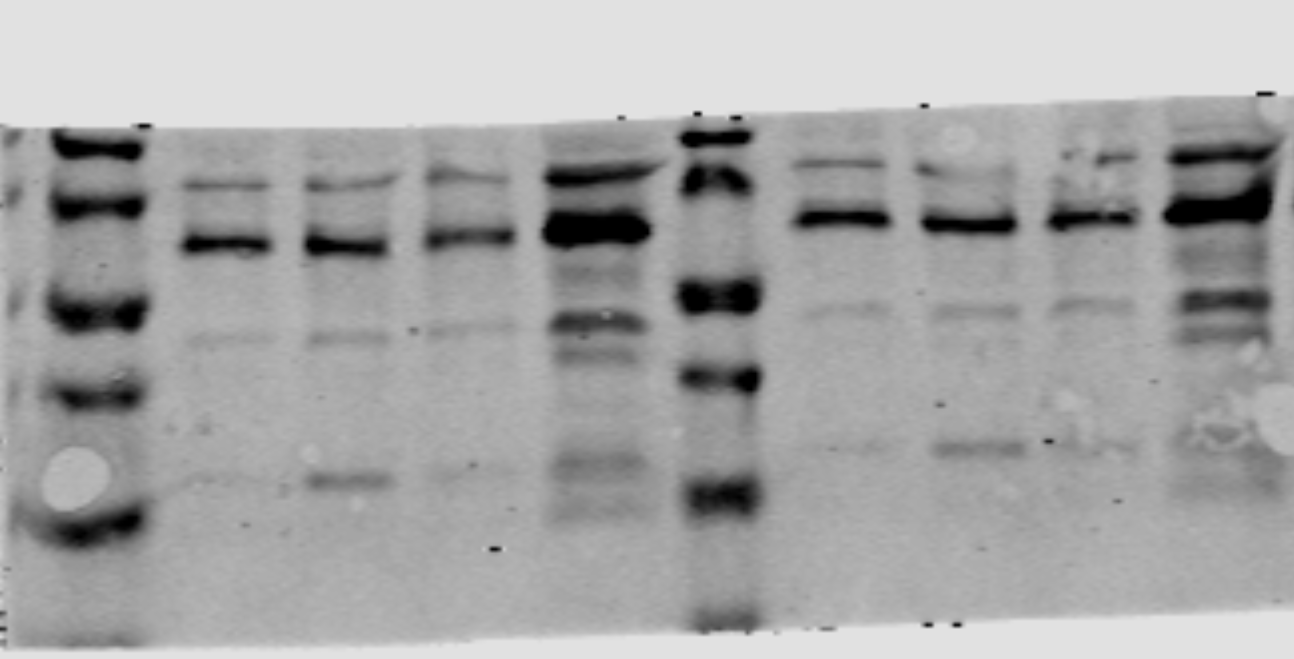

Supplement: Supplementary file 2 [file DataSheet1.zip › Supplementary Information/cell-Western blot/caspase3/caspase3 3.tif]

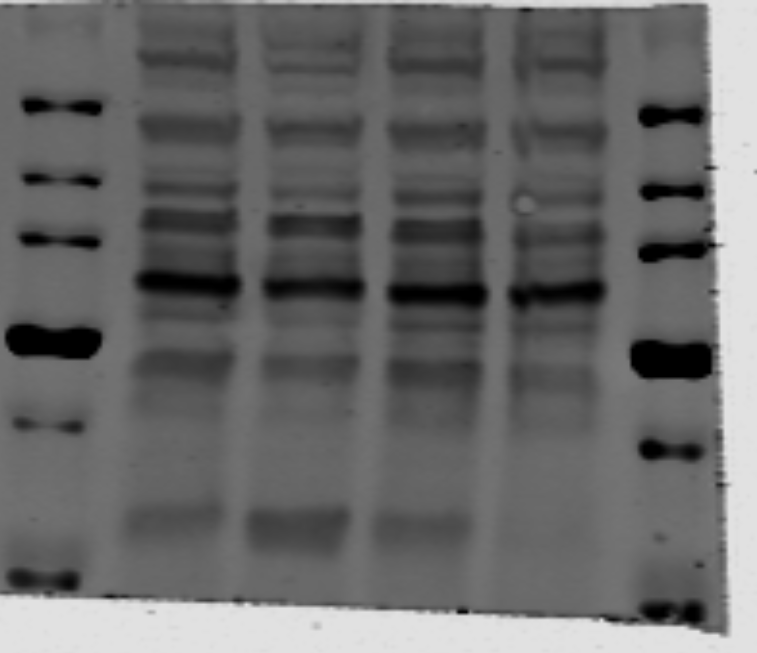

Supplement: Supplementary file 2 [file DataSheet1.zip › Supplementary Information/cell-Western blot/caspase8/caspase8-1.tif]

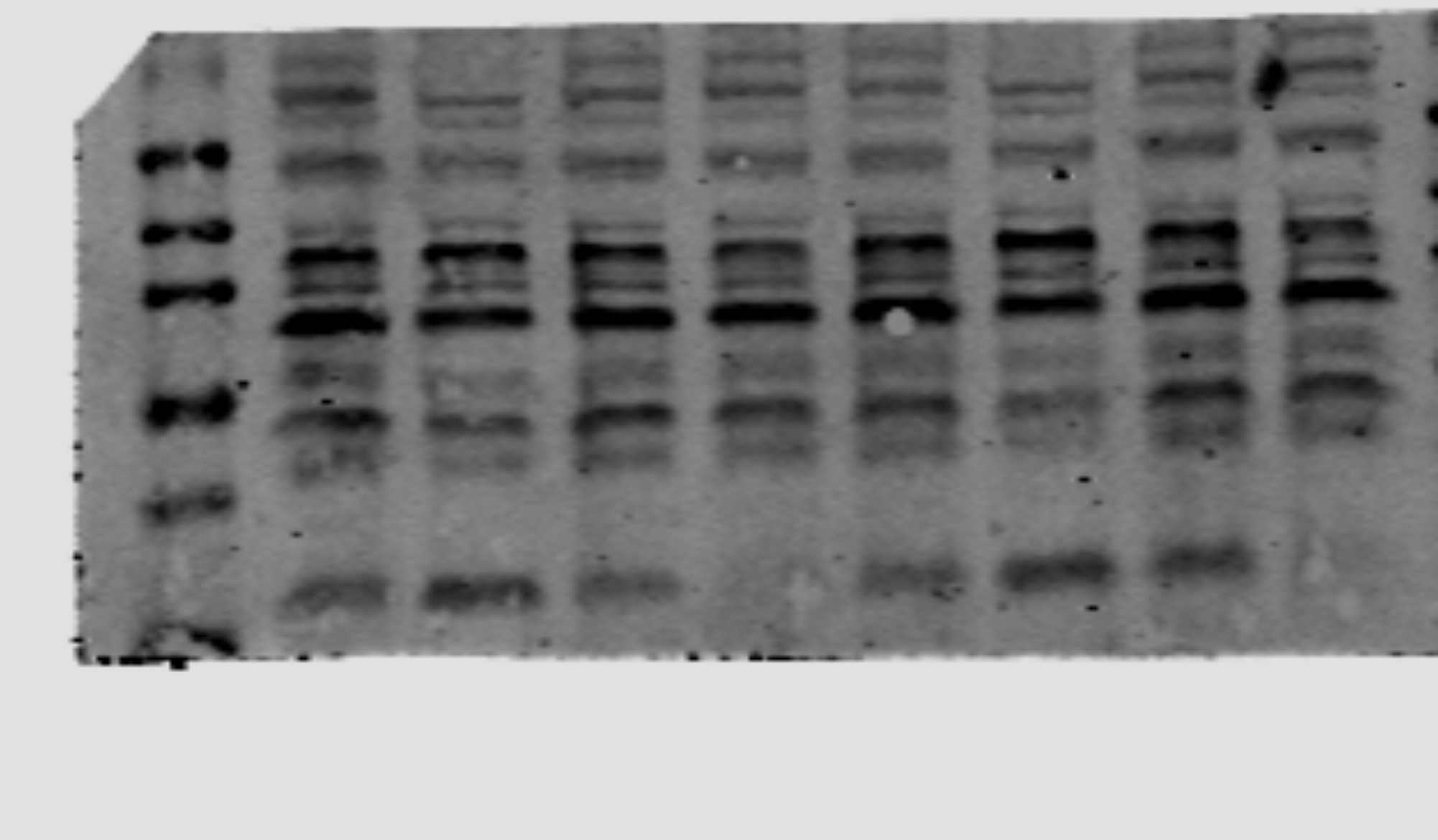

Supplement: Supplementary file 2 [file DataSheet1.zip › Supplementary Information/cell-Western blot/caspase8/caspase8-2.tif]

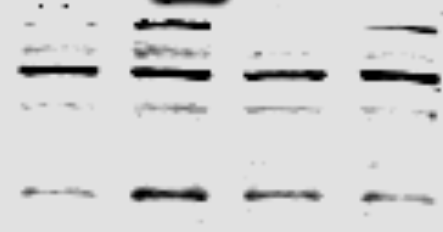

Supplement: Supplementary file 2 [file DataSheet1.zip › Supplementary Information/cell-Western blot/gsdmd/GSDMD-2.tif]

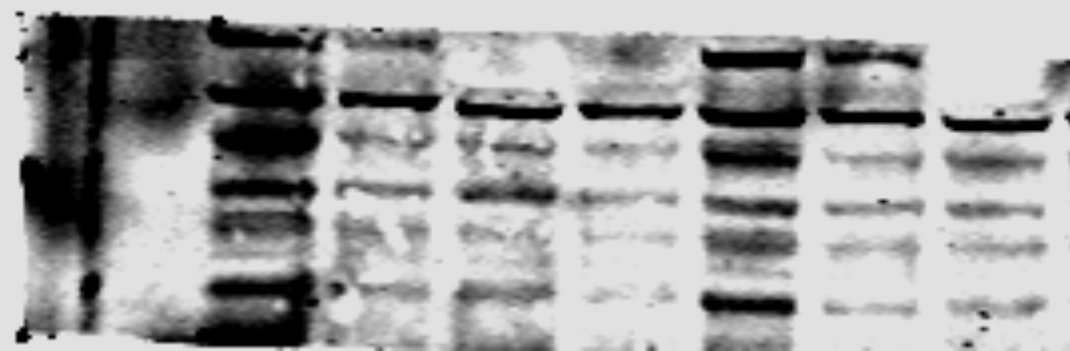

Supplement: Supplementary file 2 [file DataSheet1.zip › Supplementary Information/cell-Western blot/gsdmd/gsdmd.tif]

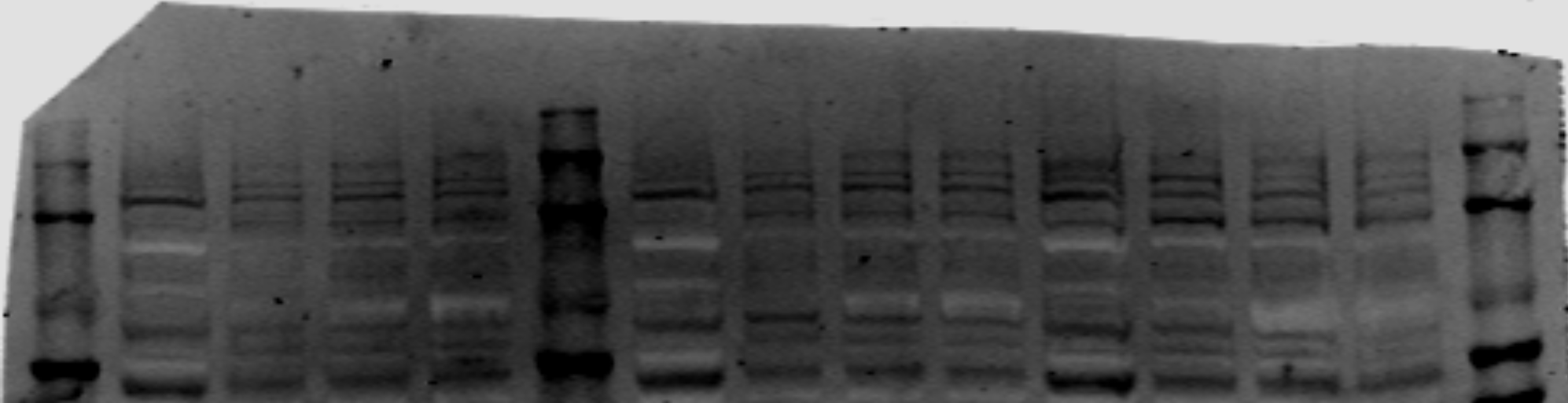

Supplement: Supplementary file 2 [file DataSheet1.zip › Supplementary Information/cell-Western blot/mlkl/MLKL.tif]

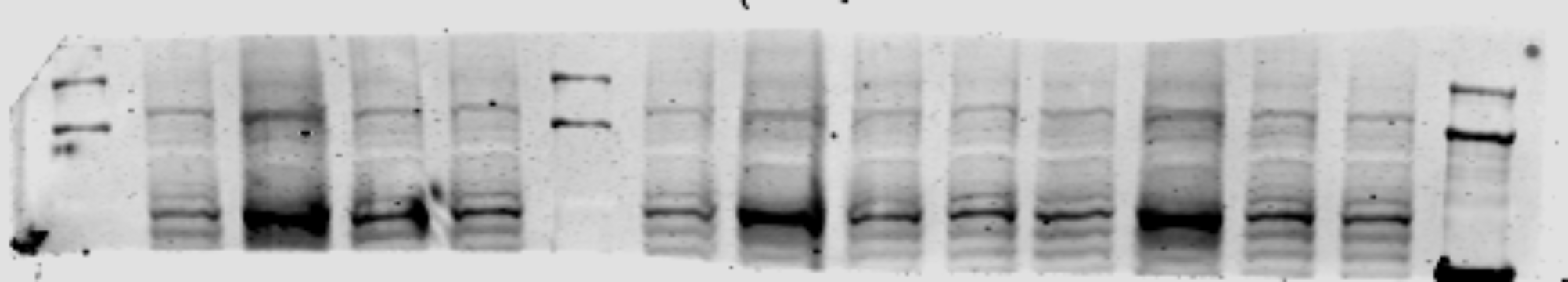

Supplement: Supplementary file 2 [file DataSheet1.zip › Supplementary Information/cell-Western blot/nfkb/nf-kb1.tif]

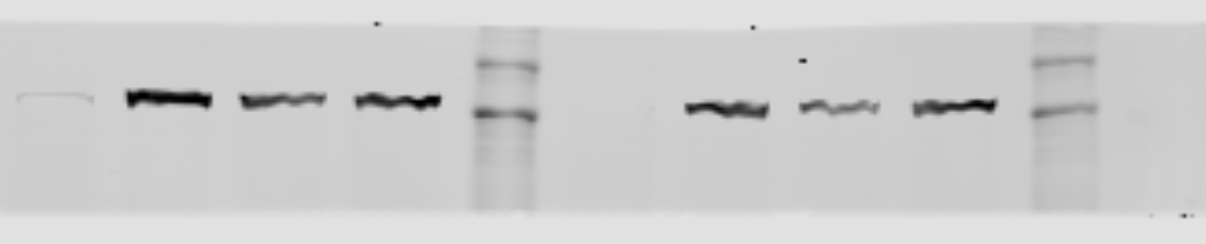

Supplement: Supplementary file 2 [file DataSheet1.zip › Supplementary Information/cell-Western blot/nlrp3/NLRP3-1.tif]

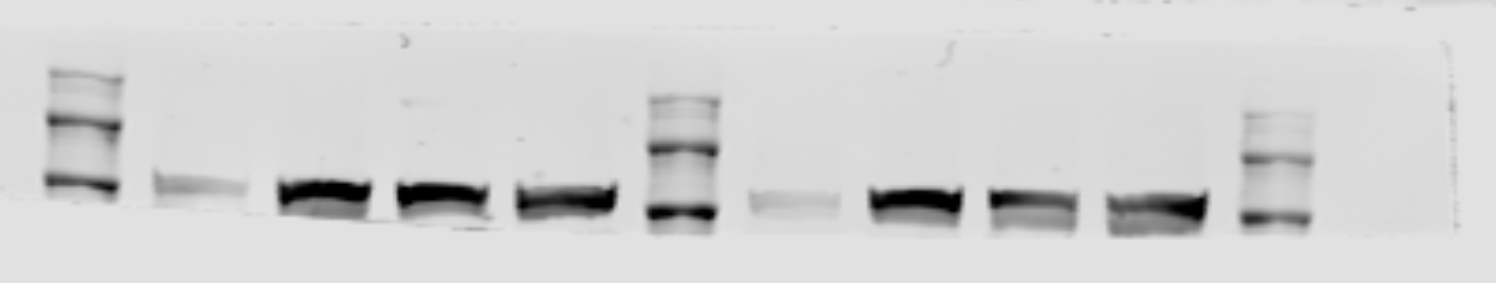

Supplement: Supplementary file 2 [file DataSheet1.zip › Supplementary Information/cell-Western blot/nlrp3/nlrp3-2.tif]

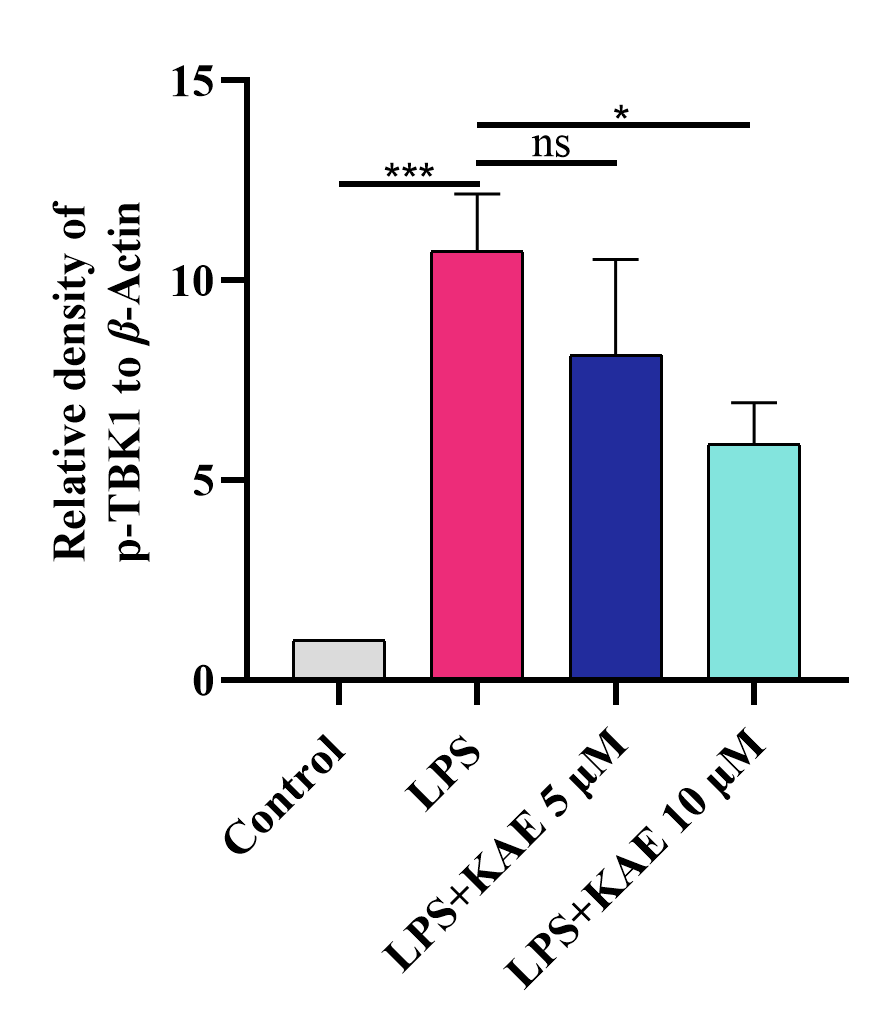

Supplement: Supplementary file 2 [file DataSheet1.zip › Supplementary Information/cell-Western blot/p-TBK1.tif]

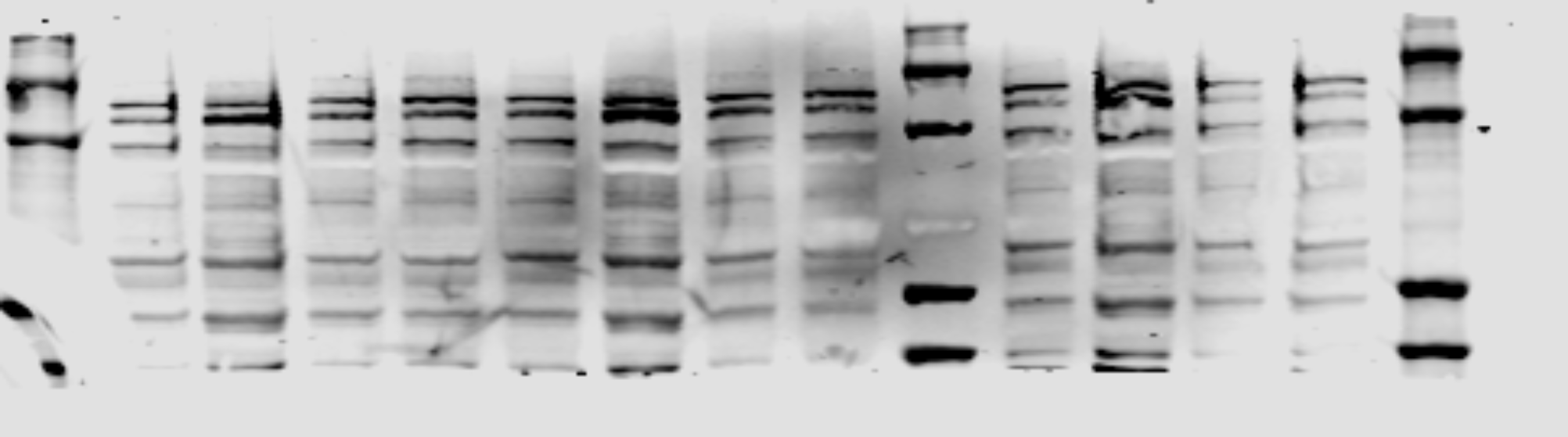

Supplement: Supplementary file 2 [file DataSheet1.zip › Supplementary Information/cell-Western blot/p-mlkl/p-mlk-1.tif]

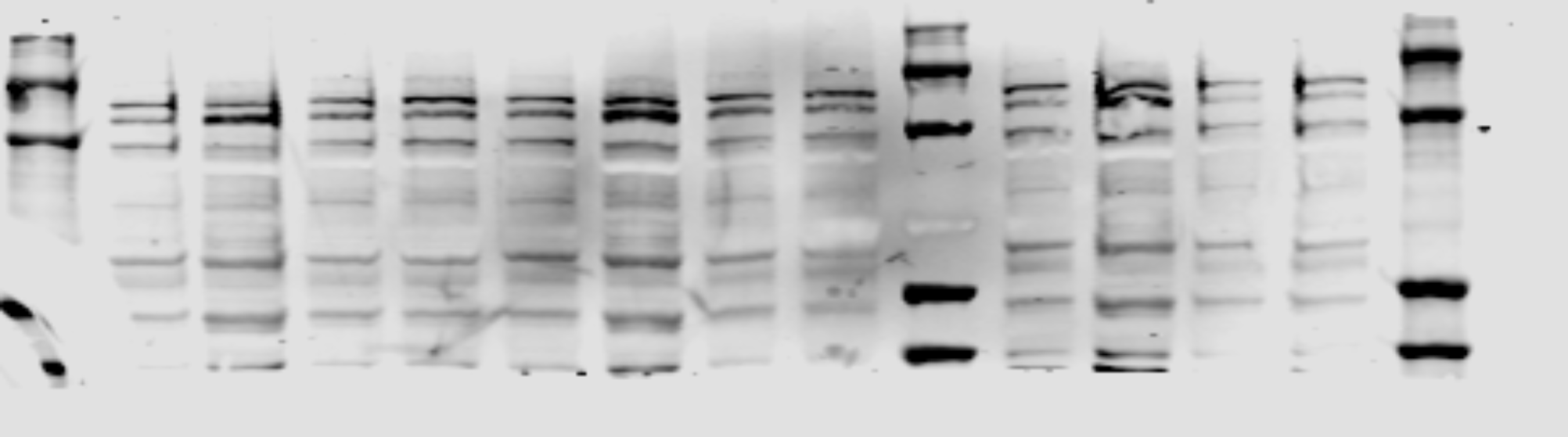

Supplement: Supplementary file 2 [file DataSheet1.zip › Supplementary Information/cell-Western blot/p-mlkl/p-mlkl-2.tif]

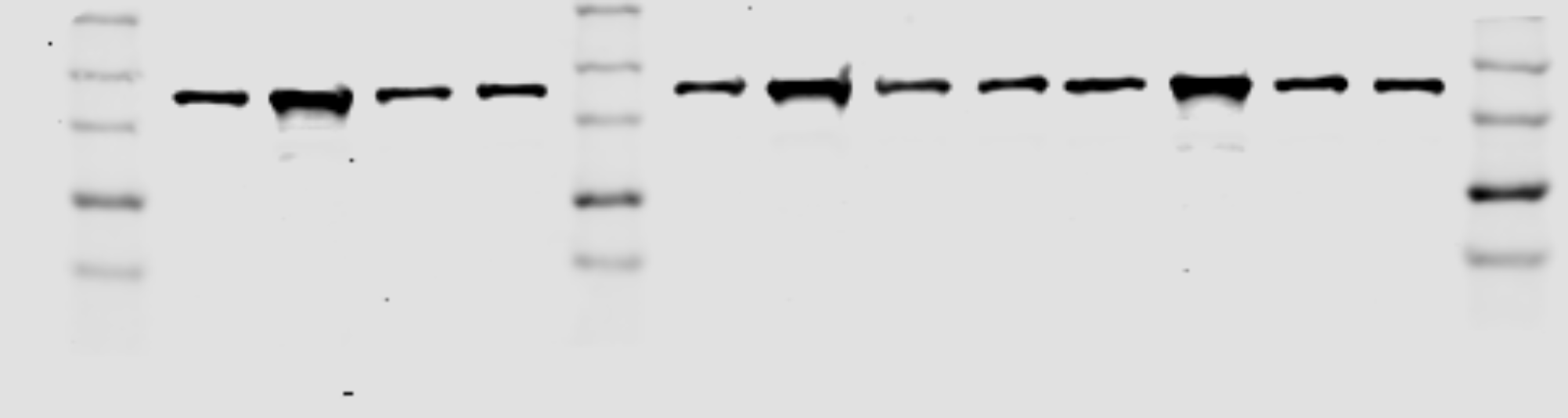

Supplement: Supplementary file 2 [file DataSheet1.zip › Supplementary Information/cell-Western blot/sting/stingkae2225.tif]
